# Multicenter Phase 2 Trial of Sirolimus for Tuberous Sclerosis: Kidney Angiomyolipomas and Other Tumors Regress and VEGF- D Levels Decrease

**DOI:** 10.1371/journal.pone.0023379

**Published:** 2011-09-06

**Authors:** Sandra L. Dabora, David Neal Franz, Stephen Ashwal, Arthur Sagalowsky, Francis J. DiMario, Daniel Miles, Drew Cutler, Darcy Krueger, Raul N. Uppot, Rahmin Rabenou, Susana Camposano, Jan Paolini, Fiona Fennessy, Nancy Lee, Chelsey Woodrum, Judith Manola, Judy Garber, Elizabeth A. Thiele

**Affiliations:** 1 Biogen Idec Hemophilia, Weston, Massachusetts, United States of America; 2 Massachusetts General Hospital, Boston, Massachusetts, United States of America; 3 Dana-Farber Cancer Institute, Boston, Massachusetts, United States of America; 4 University of Cincinnati, Cincinnati, Ohio, United States of America; 5 Loma Linda University, Loma Linda, California, United States of America; 6 University of Texas Southwestern, Dallas, Texas, United States of America; 7 University of Connecticut, Hartford, Connecticut, United States of America; 8 New York University, New York City, New York, United States of America; 9 Brigham and Women's Hospital, Boston, Massachusetts, United States of America; L' Istituto di Biomedicina ed Immunologia Molecolare, Consiglio Nazionale delle Ricerche, Italy

## Abstract

**Background:**

Tuberous sclerosis (TSC) related tumors are characterized by constitutively activated mTOR signaling due to mutations in *TSC1* or *TSC2*.

**Methods:**

We completed a phase 2 multicenter trial to evaluate the efficacy and tolerability of the mTOR inhibitor, sirolimus, for the treatment of kidney angiomyolipomas.

**Results:**

36 adults with TSC or TSC/LAM were enrolled and started on daily sirolimus. The overall response rate was 44.4% (95% confidence intervals [CI] 28 to 61); 16/36 had a partial response. The remainder had stable disease (47.2%, 17/36), or were unevaluable (8.3%, 3/36). The mean decrease in kidney tumor size (sum of the longest diameters [sum LD]) was 29.9% (95% CI, 22 to 37; n = 28 at week 52). Drug related grade 1–2 toxicities that occurred with a frequency of >20% included: stomatitis, hypertriglyceridemia, hypercholesterolemia, bone marrow suppression (anemia, mild neutropenia, leucopenia), proteinuria, and joint pain. There were three drug related grade 3 events: lymphopenia, headache, weight gain. Kidney angiomyolipomas regrew when sirolimus was discontinued but responses tended to persist if treatment was continued after week 52. We observed regression of brain tumors (SEGAs) in 7/11 cases (26% mean decrease in diameter), regression of liver angiomyolipomas in 4/5 cases (32.1% mean decrease in longest diameter), subjective improvement in facial angiofibromas in 57%, and stable lung function in women with TSC/LAM (n = 15). A correlative biomarker study showed that serum VEGF-D levels are elevated at baseline, decrease with sirolimus treatment, and correlate with kidney angiomyolipoma size (Spearman correlation coefficient 0.54, p = 0.001, at baseline).

**Conclusions:**

Sirolimus treatment for 52 weeks induced regression of kidney angiomyolipomas, SEGAs, and liver angiomyolipomas. Serum VEGF-D may be a useful biomarker for monitoring kidney angiomyolipoma size. Future studies are needed to determine benefits and risks of longer duration treatment in adults and children with TSC.

**Trial Registration:**

Clinicaltrials.gov NCT00126672

## Introduction

Tuberous sclerosis (TSC) is a tumor suppressor gene disorder characterized by the development of benign tumors (hamartomas) in multiple organs [1]. It is an autosomal dominant disorder with high penetrance but variable expression, and the majority (60–70%) of newly diagnosed individuals have a spontaneous mutation. There are approximately 50,000 individuals living with TSC in the U.S. and approximately 1 million worldwide. The diagnosis is based on a series of major and minor clinical criteria [2]. The disease causing genes (*TSC1* and *TSC2*) have been identified and the “two-hit” mechanism of disease has been demonstrated in many tumors [3], but haploinsufficiency may explain some aspects of brain involvement [4], [5]. TSC patients can have a number of medical problems including epilepsy, cognitive impairment, behavioral disorders, brain lesions (tubers, subependymal nodules and subependymal giant cell astrocytomas), kidney angiomyolipomas, kidney cysts, early onset kidney cancer, skin tumors (facial angiofibromas), cardiac tumors (rhabdomyomas) and pulmonary abnormalities including lymphangioleiomyomatosis (LAM) [6], [7], [8].

Hamartin and tuberin, the *TSC1* and *TSC2* gene products, form a complex that inhibits mammalian target of rapamycin (mTOR) in a conserved cellular signaling pathway (PI3kinase- Akt-mTOR pathway) that regulates nutrient uptake, growth and protein translation [9], [10], [11]. Rapamycin (also known as sirolimus, Rapamune®) is an mTOR kinase inhibitor that is an approved drug for immunosuppression following kidney transplantation, and rapamycin analogs have recently been approved for the treatment of kidney cancer and pancreatic neuroendocrine tumors [12], [13], [14]. Rapamycin normalizes dysregulated mTOR signaling in cells that lack normal hamartin or tuberin. Furthermore, there is now substantial evidence that inhibiting mTOR kinase in preclinical rodent models of TSC may be useful for treating tumors and brain manifestations of TSC [5], [15], [16], [17], . It is important to note that tumors and brain tissue from rodent models for TSC have similar abnormalities in mTOR signaling (with hyperphosphorylated S6 kinase and ribosomal subunit S6) as brain tumors (subependymal giant cell astrocytomas) [19], kidney tumors (angiomyolipomas) from TSC patients [20], and abnormal lung tissue from LAM patients [21].

Kidney angiomyolipomas are a common and problematic major clinical feature of TSC. These tumors consist of blood vessels, smooth muscle, and fat cells. They occur in approximately 75% of TSC patients over the age of 6–8 years [6], [22], and are a significant cause of morbidity and mortality [23]. Growth of kidney angiomyolipomas associated with TSC is common and has been reported in 47% of TSC patients in a cohort of 60 children (ages 1–18) [22], and in 91% in a cohort of 32 TSC patients (ages <1 year to 36 years) [24]. Kidney angiomyolipomas can also cause pain, bleeding, and varying degrees of renal failure. The current standard of care is to monitor kidney angiomyolipomas with imaging every 1–3 years. If there is evidence of rapid tumor growth, tumor size >4 cm, or symptoms, surgical removal or vascular embolization is recommended. In a recent review of 102 patients with kidney angiomyolipomas followed for a median of 4 years, 25 required intervention [25]. In that series, vascular embolization was the most common intervention recommended, but was often problematic as 6 of 19 (31.5%) patients had complications (post embolization syndrome-3, abscess-1, nonfunctioning kidney-1, refractory hypertension-1). Development of effective medical therapy for these tumors could allow some patients to avoid the risks of one or more invasive procedures.

We implemented this multicenter sirolimus trial to provide efficacy and safety data regarding the treatment of TSC associated angiomyolipomas with sirolimus. In addition to the primary endpoints evaluating safety and kidney angiomyolipoma response, we investigated the utility of mTOR inhibitor treatment for many common clinical features of TSC by collecting secondary endpoint data on liver angiomyolipomas, subependymal giant cell astrocytomas (SEGAs), tubers, subependymal nodules (SENs), seizures, skin lesions (facial angiofibromas, hypomelanotic macules, shagreen patches, forehead plaques), renal cysts, kidney function, and lung function in those individuals with LAM. We also collected data on TSC gene mutations and explored the utility of serum vascular endothelial growth factor D (VEGF-D) as a biomarker for TSC associated kidney angiomyolipomas.

## Methods

### Enrollment and study design

Participants were recruited at 6 clinical sites in the U.S. Five of the clinical sites were comprehensive TSC clinics (Boston, Cincinnati, Loma Linda, Hartford, New York) and one was a Urology clinic (Dallas). The protocol was approved by the appropriate institutional review boards (IRBs) which included: 1) Dana-Farber Cancer Institute IRB; 2) UT Southwestern Medical Center IRB; 3) Connecticut Children's Medical Center IRB; 4) Cincinnati Children's Medical Center IRB; 5) NYU School of Medicine IRB; 6) Loma Linda University IRB. The study was registered with clinicaltrials.gov (ID NCT00126672) and conducted according to institutional and national guidelines. This was an open label, single arm, multicenter study with a Simon two-stage design [26]. Initially 13 patients were enrolled; an additional 23 patients were enrolled after the first partial response was observed in the initial cohort. After written informed consent was obtained, participants were registered, baseline testing was completed, and oral treatment with sirolimus was started. A copy of the original protocol is included in the online supporting information files (Protocol S1).

### Eligibility and Exclusion Criteria

Eligible patients were 18–65 years old with at least one kidney angiomyolipoma ≥2 cm in diameter and a diagnosis of TSC or LAM. Additional inclusion criteria included adequate renal, liver, and bone marrow function (Cr<4.1, SGOT, SGPT, TBili, Alk Phos all<2× normal, hematocrit >30, normal WBC, and platelets >100,000). Patients were excluded if they had unstable seizures (recent changes in seizure pattern or use of anti-epileptic agents), clinically significant bleeding from kidney angiomyolipoma(s), severe LAM (dependent on continuous supplemental oxygen or limited performance status), evidence for accelerating renal dysfunction or acute renal failure, renal cell carcinoma (suspected or known), active infection, recent use of other investigational agent in the 30 days prior to study entry, or prior history of coronary artery disease. Pregnant or nursing women were also excluded.

### Objectives

The primary objectives were the evaluation of kidney angiomyolipoma response and tolerability of sirolimus. The overall response rate was determined using magnetic resonance imaging (MRI) before and after treatment according to the Response Evaluation Criteria in Solid Tumors (RECIST) [27]. According to RECIST, kidney angiomyolipoma size is defined as the sum of the longest diameters (sum LD) of up to five target lesions per kidney (in cm); a partial response is ≥30% decrease in sum LD; progressive disease is ≥20% increase in sum LD; a complete response is complete tumor regression; all other changes are considered stable disease. The percent change in sum LD was calculated using this formula: ((sum LD time 2 - sum LD time 1)/sum LD time 1)*100. NCI Common Toxicity Criteria, version 3.0, were used to assess toxicity. Secondary objectives included evaluation of other TSC lesions (SEGAs, liver angiomyolipomas, tubers, SENs, kidney cysts, skin lesions) before and after 52 weeks of sirolimus treatment, pulmonary function testing, renal function testing, and collection of samples for correlative biomarker analyses. Exploratory objectives included longer term follow up (at 18 months and 24 months) in two subgroups (ON SIROLIMUS AFTER WK 52 and OFF SIROLIMUS AFTER WK 52), and investigation of serum VEGF-D as a biomarker.

### Sirolimus dosing and drug levels

Sirolimus treatment was initiated with a loading dose of 6 mg by mouth on day 1 followed by 2 mg by mouth daily. Sirolimus is FDA approved for use after kidney transplantation and this is the standard dose used in the kidney transplant population. The dose was then adjusted to maintain a target blood level of 3–9 ng/ml for the first 16 weeks. After week 16, the dose of sirolimus was increased to a target level of 9–15 ng/ml unless there was evidence for a partial response or complete response by kidney MRI. There was no change in dose for those with a partial response or complete response at week 16. Trough sirolimus levels were checked every 8–12 weeks (at 24 weeks, 32 weeks, 40 weeks, and 52 weeks) in all patients until the 12-month (week 52) study visit. If the sirolimus dose was below target, the dose was increased by 1–2 mg until the target trough level was achieved. Sirolimus levels were checked every 2–3 weeks while the dose was adjusted.

There was a wide range of sirolimus doses required to maintain target trough levels. At the end of year 1 (week 52), the mean daily dose was 6.7 mg per day. The lowest dose was 1 mg every other day and the highest dose was 24 mg per day. Sirolimus is metabolized by CYP3A4 and at the dose extremes, subjects were taking concomitant drugs that likely had a significant impact on drug metabolism. The participant on the lowest dose was also on verapamil, a CYP3A4 inhibitor, which should reduce the clearance of sirolimus. The participant on the highest dose was on primidone, a CYP3A4 inducer, which should increase the clearance of sirolimus. During our study there were 5 participants that exceeded the upper limit of our target levels (15 ng/ml) at any time. Almost all participants (28/33, 85%) required a one time dose increase per protocol at week 16 because they did not have a partial response.

We had a low threshold to hold sirolimus doses during minor infections or to allow symptomatic mouth ulcers to heal, so many participants had drug held at some point during the course of the study. Per our protocol, if there were more than 10 consecutive missed doses, these were made up at the end of year 1 on study so that 28 participants received ∼52 weeks of sirolimus treatment (range 49–56 weeks). We monitored sirolimus levels every 8–12 weeks in order to avoid toxicity by holding or reducing doses if the target drug level was exceeded. There is substantial evidence indicating a single steady state trough sirolimus level corresponds well to drug exposure because it has been well documented that the 24 hour trough sirolimus level correlates well with the AUC (area under concentration time curve) [28], [29]. Drug level monitoring allowed relatively rapid dose adjustments to maintain target levels and was also useful for verifying that participants were taking sirolimus as directed throughout the study.

### VEGF-D assay

Serum was collected using standard clinical red top serum collection vacutainer tubes with no additive. After clot formation, the tube was spun at low speed, the serum was collected, aliquoted, frozen, and shipped overnight on dry ice to the Dabora Lab at Brigham and Women's Hospital, Boston, MA. VEGF-D levels were measured using ELISA (Quantikine Human VEGF-D Immunoassay kit from R&D Systems, catalog number DVED00). The VEGF-D ELISA was done according the manufacturer's protocol (Quantikine Human VEGF-D Immunoassay kit from R&D Systems) with the following modifications: 1) known concentrations of 125, 250, 500, 1000, 2000, 4000, and 40,000 pg/ml were used to generate the standard curve; 2) we used a correction wavelength of 550 nm instead of 540 nm because 540 nm was not an option on our plate reader (THERMOmax microplate reader, Molecular Devices Corp.); 3) sample concentrations were extrapolated from the standard curve using GraphPad Prism software (version 4.01) using the one-site competition option in order to optimize the fit of the standard curve [30]. All standards were run in quadruplicate (all readings were within 25% of the mean of all 4 readings). All clinical samples were run in duplicate and duplicates deviated by no more than 25% from the average of the two readings. As there was some plate to plate variability in VEGF-D assay results for identical samples, the baseline (week 0) and week 52 samples from each subject reported here were run on the same plate so concentrations for both time points were determined from the same standard curve.

### Statistical considerations, sample size, and statistical analysis

Since this trial was designed in 2003 (before there was any published clinical data available on the efficacy of mTOR inhibitors for TSC related tumors), a two-stage adaptive design was employed because it would allow early stopping if there was no evidence for response in a small cohort of subjects, but continuation if there was some early evidence of efficacy [26]. Our protocol was designed to enroll an initial cohort 13 eligible subjects; if there were no responses observed among the first 13 eligible subjects, the study would have been discontinued and the regimen declared unpromising. However, if one response was observed among the first 13 subjects, then enrollment would continue and an additional 22 eligible patients would be included. The sample size was determined based on the following: if 4 or more responses are observed among 35 total eligible patients, the regimen will be considered worthy of further study. Alternatively, if 3 or fewer responses are observed, the regimen will be declared unpromising. According to power calculations, a study with this design had a 51% probability of stopping early if the true response rate was 5%, 8.4% probability of declaring the treatment effective at the end of the study if the response rate was 5%, and 90% probability of declaring the treatment effective if the response rate was 20%. Note: we enrolled 36 subjects (with IRB approval) because the final two subjects were recruited at approximately the same time.

GraphPad Prism software (version 4.01) was used for all data analysis, with a p-value≤0.05 indicating statistical significance. A paired *t* test or Wilcoxon signed rank test (for non-parametric data) was used for paired quantitative variables. An unpaired *t* test or Mann-Whitney test (for non-parametric data) was used for unpaired quantitative variables. A two-sided Fisher's exact test was used for categorical variables. Spearman correlation was used to investigate the correlation between kidney angiomyolipoma size and VEGF-D levels.

### Protocol Deviations/Violations and Amendments

There were a total of 64 protocol deviations/violations that were reported to the Dana-Farber IRB between March 2007 and August 2009. Deviations/violations occurred in these categories: baseline testing issue-2, baseline testing timing issue-2, consent issue-6, documentation-1, eligibility issue-7, eligibility issue and baseline testing timing-2, enrollment target issue-2, lab test missing-2, lab test timing-7, missing data-1, study conduct issue-3, study drug dispensing issue-1, study drug issue-1, treatment issue-10, visit date issue-13, visit date and leaving study early-3, visit date and missing data-1. These deviations/violations were judged to have minimal impact on risks to the subjects and did not affect overall data quality. Many of the violations/deviations prompted amendments to allow clinically and scientifically acceptable changes that would reduce the number of future deviations/violations. All deviations/violations were submitted to the IRB for review, and our corrective action plan was approved in all cases. We have included the complete list of deviations/violations with the description as reported to the Dana-Farber IRB (Table S13).

During the course of this study we submitted a total of 3 amendments that modified the study design (Amendments 15, 17, and 20). Amendment 15 was submitted in August 2007 and approved in February 2008. It included the following changes in eligibility requirements: the platelet count requirement was changed to >100,000 (from normal), the white blood count (WBC) requirement was changed to absolute neutrophil count (ANC) >1500 (from WBC normal), eligible age was changed to 3–65 years (from 16–65 years), exclusion of those with kidney bleed was changed to exclusion of those with clinically significant kidney bleed; baseline renal function required was changed to eGFR of at least 30 (from creatinine less than 4.1 ). There were also minor changes made in timing of tests or timing of visits including these: baseline kidney MRI should be done within 4 weeks (instead of 2 weeks); sirolimus levels should be done within 2 weeks of target date. A change was also made to allow CT scanning instead of MRI for patients unable to undergo MRI testing. A number of other minor changes were made to improve or clarify study details. Amendment 17 was submitted in February 2008 and approved in March 2008. The change requested was to increase target enrollment from 36 to 76 participants. However, we were unable to obtain adequate funding to increase the enrollment so unable to implement this change in enrollment. Amendment 20 was submitted in January 2009 and approved in June 2009. This change was requested to allow additional sirolimus treatment during months 12–24 if the treating site investigator judged that this was in the best interest of the study participant. Additional details regarding this amendment can be found in the results section. The final protocol version and CONSORT checklist are included in the supporting information (Protocol S2, Checklist S1).

## Results

### Enrollment and Baseline Characteristics

We enrolled 36 adults between December 2005 and April 2008. All participants met diagnostic criteria for TSC [2] and 21 subjects also had clinical evidence for LAM (19 women and 2 men). Baseline characteristics are shown in Table 1. See Table S8 for details of TSC gene mutation testing.

**Table 1:** Baseline characteristics of enrolled subjects.

Characteristic	Number (range)	%	Characteristic	Number (range)	%
Ave. age at study enrollment, years	34 (19–60)		**BRAIN**		
Ave. age when TSC diagnosed, years	13 (0–60)		Subependymal nodules (SENs)* present	30	83%
Diagnosis			Subependymal nodules (average number)	3.4 (0–8)	
TSC	13	36%	Cortical Tubers*	30	83%
TSC & LAM*	23	64%	None	4	11%
Sex			Minimal (0–2)	3	8%
Male	10	28%	Mild (3–5)	3	8%
Female	26	72%	Moderate (6–10)	11	31%
Race			Severe (11+)	13	36%
White	34	94%	Unknown	2	6%
Black	1	3%	Subependymal Giant Cell Astrocytoma*	13	36%
Other	1	3%	Seizures		
ECOG performance status 0	32	89%	chronic	7	19%
ECOG performance status 1	4	11%	prior history	20	56%
			never	8	22%
**RENAL**			unknown	1	3%
Kidney angiomyolipomas*			Cognitive impairment	15	42%
Average sum of the longest diameters (sum LD), cm	21.2 (2.0–51.8)		None	21	58%
Average number of measurable kidney tumors per person	3.8 (1–10)		Mild	10	28%
Angiomyolipoma diameter >4 cm	25	69%	Moderate	4	11%
Angiomyolipoma diameter >10 cm	7	19%	Severe	1	3%
Prior invasive kidney procedures	18	50%	Psychological/Behavioral Issues	18	50%
Nephrectomy	7	19%	ADHD	1	3%
Biopsy	3	8%	Anxiety	5	14%
Vascular emobolization	6	17%	Autism	1	3%
More than one	2	6%	Depression	2	6%
Kidney Cysts**	22	61%	More than one	9	25%
None	14	39%	None	18	50%
0–2 Small Cysts (<2 cm)	5	14%			
>2 Small Cysts (<2 cm)	6	17%	**OTHER**		
>2 Cysts with at least one >2 cm	9	25%	History of cardiac rhabdomyoma*	4	11%
Classic Polycystic Disease	2	6%	Liver angiomyolipoma	15	42%
Chronic renal insufficiency (Cr≥1.5 mg/dl)	6	17%	Retinal hamartoma*	10	28%
Proteinuria	4	11%	Genetics		
Hematuria	4	11%	*TSC2* mutation	14	39%
			*TSC1* mutation	0	0%
**SKIN**			No mutation identified	4	11%
Facial angiofibromas*	35	97%	Mutation unknown (not tested or test inconclusive)	18	50%
None	1	3%			
Macular lesions only on cheek	3	8%			
Papular lesions, <3 mm diameter	22	61%			
Papular lesions, >3 mm diameter	10	28%			
Hypomelanotic macules*	24	67%			
Shagreen patch*	22	61%			
Ungual/subungual fibromas*	20	56%			
Forehead plaque*	14	39%			

*TSC major feature.

**TSC minor feature.

### Regression of kidney tumors during the first 52 weeks

After baseline testing was completed, participants were started on oral sirolimus at a dose of 6 mg on day 1, then 2 mg per day. The sirolimus trough level was measured 1–3 weeks later and the dose was adjusted to an initial target range of 3–9 ng/ml. Kidney angiomyolipoma size was evaluated by MRI at baseline and weeks 16, 32, and 52 during year 1 (CT was used for 1 subject who was unable to undergo MRI). In those patients with a partial response at week 16, drug treatment continued at the same dose. The sirolimus dose was increased for all other patients to achieve a trough target range of 9–15 ng/ml. Sirolimus treatment continued for 52 weeks. Tolerability and drug levels were monitored every 8–12 weeks.

**Figure 1:**
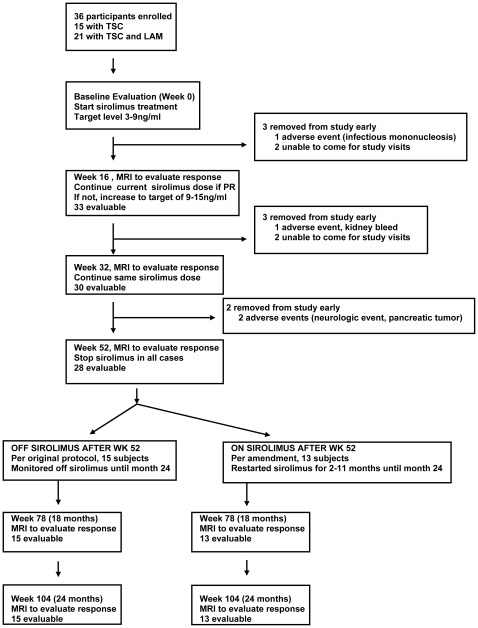
Enrollment Chart.

There were 36 adults enrolled and 28 were evaluable at 12 and 24 months (see Figure 1). There were 8 participants who were removed from the study early. This included 4 who were unable to keep follow-up study appointments, and 4 who were taken off study because of a serious adverse event that was judged unrelated to drug therapy (1-hospitalized with complicated infectious mononucleosis, 1-diagnosed with a pancreatic neuroendocrine tumor, 1-hospitalized with a kidney angiomyolipoma hemorrhage, 1-hospitalized with a neurologic event). All of these patients recovered from these events. All subjects were included in the safety analysis. All but 3 had at least one kidney MRI scan so were evaluable for response of kidney angiomyolipomas to sirolimus treatment.

**Figure 2:**
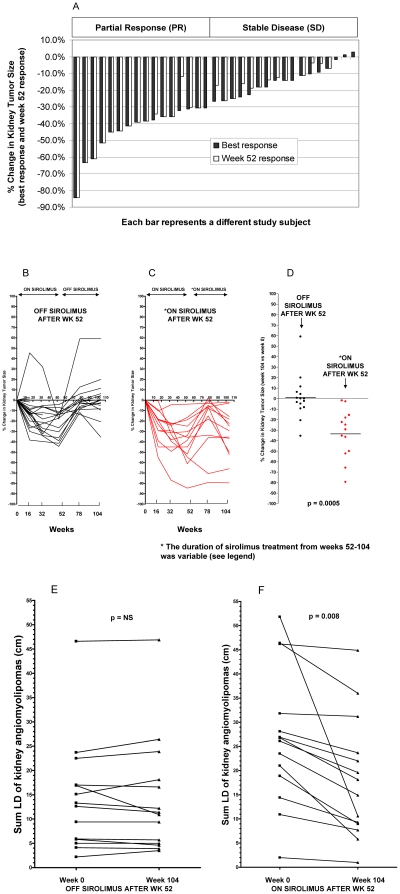
Kidney angiomyolipoma regression with sirolimus treatment. Panel A) percent change in kidney tumor size (sum LD) for individual cases (black bars-best response during year one on study; adjacent white bars-week 52 response for same subject). All subjects were treated with sirolimus from weeks 0 to 52. Panels B and C) percent change in kidney tumor size compared with baseline for kidney tumors at each time point. After week 52, a subset (Panel B) was observed off treatment (black, OFF SIROLIMUS AFTER WK 52 group, n = 15). Another subset (Panel C) received additional study drug treatment after week 52 (red, ON SIROLIMUS AFTER WK 52 group, n = 13). Panel D) percent change in kidney tumor size at 24 months (104 weeks) for indicated groups. Panel E) kidney tumor size at week 0 and week 104 (month 24) for the OFF SIROLIMUS AFTER WK 52 group. Panel F) kidney tumor size at week 0 and week 104 (month 24) for ON SIROLIMUS AFTER WK 52 group.

According to RECIST criteria [27], the overall response rate was 16/36 (44.4%, 95% CI, 28 to 61); all were partial responses. There were 17/36 (47.2%) with stable disease and 3/36 (8.3%) were not evaluable because they were removed from study prior to a follow-up MRI. There were none with progressive disease or a complete response. In those individuals who completed 52 weeks of treatment, there were 15/28 with a partial response (53.6% response rate, 95% CI, 35 to 72%) and 13/28 (46.4%) with stable disease. The mean (± standard deviation) number of kidney angiomyolipomas per patient was 3.8±2.2. Most participants (69%) had at least 1 kidney angiomyolipoma ≥4 cm, and 19% had at least 1 kidney angiomyolipoma ≥10 cm. The mean (± standard deviation) kidney angiomyolipoma size (sum LD) at baseline was 21.2 cm±14.6, and after 52 weeks, decreased to 14.5±11.3 cm. The mean percent decrease in kidney tumor sum LD at week 52 was 29.9% (95% CI, 22–37%, n = 28), see Figure 2. In the 28 that completed 52 weeks of treatment, the majority had their best response at week 52 (13), but in some cases the best response was at week 16 (4) or week 32 (6). The best response was at 24 months in 5 cases (see next section). There were 33 with evaluable disease, but 28 that completed all 52 weeks of treatment. The subject with progressive disease during sirolimus treatment at weeks 16 and 32 had a single small tumor that increased in size from 2.2 cm (baseline) to 3.2 cm (week 16, 45% increase in sum LD). Details regarding timing of response, numbers of tumors, and kidney tumor size are shown in Table S1. In a post-hoc comparison of response rates at different clinical sites, we did note that one site had a better response rate than the others. This type of comparison was not part of our study design and the numbers are small so we are unable draw any firm conclusions except to suggest that future multicenter TSC trials should evaluate differences across sites so that potential biases can be investigated. MRI images of kidney angiomyolipoma regression are shown in Figures S1, S2, S3 .

### Changes in kidney tumor size during months 12–24

Assessing kidney tumor size during months 12–24 was another objective. In the original version of our protocol, this was a secondary objective and the plan was to stop sirolimus at week 52 and evaluate kidney tumor size at 18 and 24 months in all subjects. However, tumor regrowth after stopping sirolimus treatment was observed in a similar single institution study [31] that was published while our trial was ongoing, so we amended our protocol to allow additional sirolimus treatment during months 12–24. This additional sirolimus treatment was offered to those individuals who had a partial response or stable disease if the treating study physician felt it was in the best interest of the participant. There were 14 patients who were eligible for sirolimus treatment during year 2 because the others had completed (or almost completed) all 24 months by the time the amendment was approved. Of these patients, additional treatment was offered to 13 participants. The impact of additional sirolimus treatment during months 12–24 is shown in Figure 2 and Table S1.

Of those in the OFF SIROLIMUS AFTER WK 52 group (n = 15) who received no additional study drug during months 12–24, there was 1 participant with a partial response, 13 with stable disease, and 1 with progressive disease at 24 months. In contrast, of those in the ON SIROLIMUS AFTER WK 52 group (n = 13) who received additional treatment during months 12–24, there were 6 with a partial response, 7 with stable disease, and 0 with progressive disease at 24 months. The difference in the proportion of subjects with a partial response (versus stable disease plus progressive disease) was significant between these two treatment groups (p = 0.029, two-sided Fisher's exact test). The duration of sirolimus treatment in the ON SIROLIMUS AFTER WK 52 group varied because of logistic issues (delays in IRB approval for amendment, time needed to obtain additional study drug supply, and scheduling issues). In all cases, there was a period of time off of sirolimus (mean time off was 24 weeks, range 4–44 weeks) prior to restarting study drug. The average total duration of treatment (including the first 52 weeks) was 78 weeks (range 60–100). In 2 cases, the month 24 visit was delayed (to 26 and 28 months respectively). The target sirolimus trough level for those on drug treatment during months 12–24 was 3–15 ng/ml. The dose and frequency of drug level monitoring was at the discretion of the treating study physician. See additional details in Table S1. On average, tumor regrowth back to baseline was observed in the OFF SIROLIMUS AFTER WK 52 group (n = 15), but not in the ON SIROLIMUS AFTER WK 52 group (n = 13), and there was substantial variation in individual cases. As shown in Figure 2C,D, the percent change at 24 months (104 weeks) in the OFF SIROLIMUS AFTER WK 52 group was 1.1%, but was −33.5% in the ON SIROLUMUS AFTER WK 52 group ( p<0.001, Mann-Whitney test). Consistent with this, if kidney tumor size (sum LD) at week 104 is compared with kidney tumor size at baseline, mean tumor size was similar in the OFF SIROLIMUS AFTER WK 52 (p not significant for week 0 versus week 104, Figure 2E), but significantly smaller in the ON SIROLIMUS AFTER WK 52 group (p = 0.008 for week 0 versus week 104, paired t test, Figure 2F). Because a comparison of two groups with different treatments from months 12–24 was not part of the original study design, all comparative analyses were done post-hoc and these data are considered exploratory.

### Toxicity

Overall, sirolimus was reasonably well tolerated and unexpected toxicities were not observed. Common drug related grade 1–2 events with a frequency of >20% included: stomatitis, hypertriglyceridemia, hypercholesterolemia, bone marrow suppression (anemia, mild neutropenia, leucopenia), proteinuria, and joint pain. Drug related grade 3 events were rare and included:1-lymphopenia, 1-headache, 1-weight gain. These events did not require intervention (headache, weight gain) or were clinically insignificant (lymphopenia). See Table 2 for a summary of common grade 1–2 adverse events and all grade 3 events. Pneumonitis is a known serious toxicity of mTOR inhibitors [32] so study staff was vigilant regarding stopping study drug promptly if respiratory symptoms were reported. There was one case of grade 1 drug related pneumonitis/pulmonary infiltrates observed. See Text S1 and Table S2 for details on serious adverse events unrelated to treatment, 4 participants with tumors unrelated to TSC, and the complete toxicity data. See Tables S9, S10, S11, S12 regarding kidney cysts, renal function, proteinuria and hematuria.

**Table 2:** Summary of toxicity data: all drug related grade 3 events and grade 1–2 events occurring in >10% of subjects.

	ALL EVENTS INCLUDING UNRELATED TOXICITIES				TREATMENT RELATED EVENTS					
All Events (36 patients)	Grade 1	Grade 2	Grade 3	All Grades (1–3)	Grade 1	Grade 2	Grade 3	All Grades (1–3)	Percent*	
Alkaline phosphatase	7	1	0	8	4	0	0	4	11.1%	
ALT, SGPT	4	1	0	5	3	1	0	4	11.1%	
Diarrhea w/o prior colostomy	10	1	0	11	3	1	0	4	11.1%	
Head/headache	7	1	1	9	3	1	1	5	13.9%	#
Hematologic-other	11	0	0	11	4	0	0	4	11.1%	
Hemoglobin	6	2	0	8	6	2	0	8	22.2%	
Hypercholesterolemia	13	3	0	16	11	3	0	14	38.9%	
Hypertriglyceridemia	12	8	0	20	10	8	0	18	50.0%	
Infection Gr0-2 neut, urinary tract	6	2	0	8	4	2	0	6	16.7%	
Infection w/unk ANC sinus	3	2	0	5	3	2	0	5	13.9%	
Infection w/unk ANC upper airway NOS	2	2	0	4	2	2	0	4	11.1%	
Irregular menses	5	1	0	6	5	0	0	5	13.9%	
Joint, pain	10	0	1	11	8	0	0	8	22.2%	
Leukocytes	15	3	0	18	11	3	0	14	38.9%	
Lymphopenia	4	0	1	5	2	0	1	3	8.3%	#
Metabolic/Laboratory-other	8	0	0	8	4	0	0	4	11.1%	
Necrosis, oral	15	6	0	21	15	6	0	21	58.3%	
Neutrophils	10	3	0	13	6	2	0	8	22.2%	
Nose, hemorrhage	6	0	0	6	5	0	0	5	13.9%	
Proteinuria	12	3	0	15	7	3	0	10	27.8%	
Rash: acne/acneiform	3	2	0	5	2	2	0	4	11.1%	
Skin-other	5	0	0	5	4	0	0	4	11.1%	
Weight gain	1	0	1	2	0	0	1	1	2.8%	#
**WORST DEGREE**	**4**	**24**	**8**	**36**	**6**	**26**	**3**	**35**		

# treatment related grade 3 event-headache, lymphopenia, weight gain.

* percent of treatment related adverse events occuring in >10% of those enrolled and/or those with a grade 3 treatment related event.

Most subjects missed some doses of sirolimus during the study for a variety of reasons. In the 28 subjects who completed 52 weeks of treatment, study drug was held per protocol for >10 days in seven subjects for medical reasons including: pneumonia-2, bronchitis-1, surgery-3, edema and herpes zoster-1. Study drug was held for >10 days for issues not specified in the protocol in another three subjects (inadequate birth control-1, symptomatic mouth ulcers-2). Study drug was restarted in most cases and the week 52 visit was adjusted per protocol so missed doses were made up at the end of year 1. Most others (∼15 subjects) missed between 1 and 9 doses of study drug for minor infections or to let mouth ulcers heal.

### Regression of brain tumors and liver angiomyolipomas

**Figure 3:**
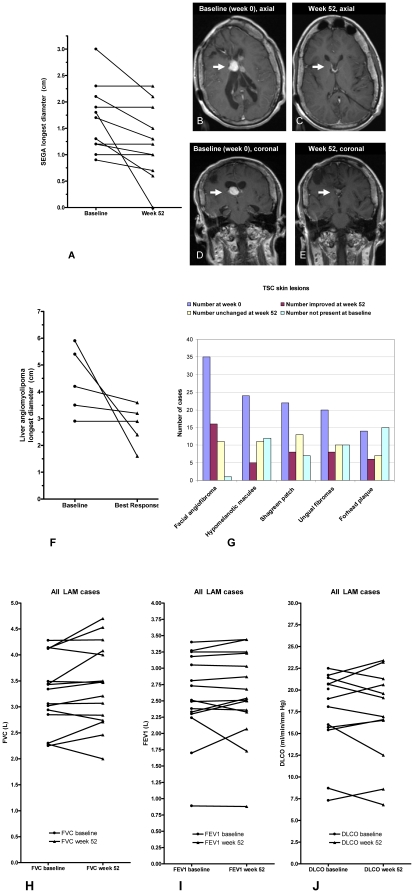
Brain tumors (SEGAs), liver angiomyolipomas, skin lesions, and lung function. Panel A shows the diameter (cm) of brain tumors (SEGAs) at baseline and week 52 in 11 participants. T1 post gadolinium MR images (courtesy of Dr. Nathaniel D. Wycliffe, Department of Radiology, Loma Linda University School of Medicine) in panels B–E show a SEGA near the right foramen of Monroe that measured 1.8 cm maximal diameter at baseline (B,D) with near complete resolution of the lesion after sirolimus treatment (C,E).. Panel F is a graph of liver angiomyolipoma size for all cases (longest diameter in cm) at baseline and at best response. Panel G shows changes in TSC skin lesions with sirolimus treatment. Panels H–J show pulmonary function data (FVC, FEV1, DLCO) at week 0 and week 52 for female participants with TSC/LAM (n = 15). See Tables S6, S7 and Figures S5, S6, S7 for data on LAM subsets.

There were 13 participants with a measurable SEGA (brain tumor) at baseline ranging in size from 0.9–3.0 cm. SEGA measurement data were available after 52 weeks of sirolimus treatment in 11 individuals (Figure 3A–E and Figure S4). Tumor regression was observed in 7 cases and SEGA size was stable in the other 4 cases. The mean SEGA diameter was 1.67±0.64 cm at baseline and 1.24±0.69 cm at week 52 (p = 0.016, Wilcoxon signed rank test). The mean decrease in SEGA diameter was 25.7% (this corresponds to ∼59% decrease in volume assuming the tumor is spherical). There was one dramatic example of a near complete response in a subject who was not a surgical candidate.

Liver angiomyolipomas were present at baseline in 15 subjects (42%) but only 5 cases had measurable disease with a longest diameter of at least 2 cm. Best responses for percent change from baseline diameter were −14.3%, 0.0%, −8.6%, −72.9%, and −64.8% (mean of −32.1%), see Figure 3F. Tumor regression was observed in all cases (n = 4) that completed 52 weeks on study. For more details, see Table S3.

### Skin lesions, lung function, tubers, SENs, and seizures

We collected baseline and week 52 data on facial angiofibromas, shagreen patch, ungual fibromas, and hypomelanotic macules. At 52 weeks, clinical site investigators reported their clinical impression on response using the following four subjective choices: improvement, no change, worse, unable to evaluate. After one year of sirolimus treatment, we observed subjective improvement in facial angiofibromas, shagreen patches, ungual fibromas, and hypomelanotic macules (Figure 3G). These observations indicated improvement in 57% (16/28) of facial angiofibromas, 18% (5/28) of hypomelanotic macules, 29% (8/28) of shagreen patches, 29% (8/28) of ungual fibromas, and 21% (6/28) of forehead plaques. See Table S5 for additional details.

The evaluation of pulmonary function before and after sirolimus treatment was another important objective of this study because LAM is an important cause of early mortality for women with TSC, 10–20% of women with TSC are at risk for developing clinically significant LAM [6], [33], and up to 30% of women with TSC have cystic lung abnormalities which are presumed to be an early stage of LAM [7], [34], [35]. Pulmonary function testing was done in 24 female participants at baseline and 18 of these had follow-up PFTs at week 52. We observed stable lung function (FVC, FEV1, DLCO) in those with TSC/LAM (n = 15, females with LAM), Figure 3H–J. In a subset with moderate LAM (n = 5), there was some improvement in FVC and FEV1: FVC increased by 0.31 L (from 3.50 L to 3.81, p = 0.06, Wilcoxon signed rank test), FEV1 increased by 0.08 L (from 2.38 L to 2.46 L, p value not significant), and DLCO was stable (13.69 ml/min/mm Hg at baseline and 13.91 ml/min/mm Hg at week 52), see Tables S6, S7 and Figures S5, S6, S7 for PFT findings in the subset with LAM.

Brain manifestations were common in study participants. Exploratory data was collected on tubers, subependymal nodules (SENs), and seizures before and after 52 weeks of sirolimus treatment. We did not observe significant changes in tubers, SENs or seizures (see Text S2 for details).

### Serum VEGF-D levels are elevated at baseline, decrease with sirolimus treatment, and correlate with kidney angiomyolipoma size

**Figure 4:**
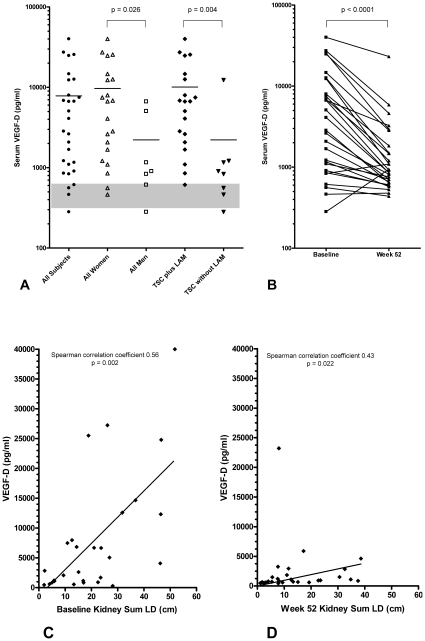
Serum VEGF-D levels are elevated at baseline, decrease with sirolimus treatment, and correlate with kidney angiomyolipoma size. Panel A shows baseline serum VEGF-D levels for all subjects and indicated subgroups (women, men, TSC/LAM and TSC). According to other studies [39], [41], mean serum VEGF-D levels in control female populations are 300–657 ng/ml (indicated by gray box, see additional details in Table S4, mean VEGF-D levels in control males is not available). Panel B shows that serum VEGF-D levels decrease after 52 weeks of sirolimus treatment. Panels C and D show the correlation between kidney tumor size (sum LD in cm) and serum VEGF-D levels (pg/ml) at baseline (Panel C) and week 52 (Panel D).

Vascular endothelial growth factor (VEGF) signaling is an important signal transduction mechanism that regulates angiogenesis and lymphangiogenesis. There are several VEGF isoforms (A–E) that bind dimers of VEGF receptors 1–3, which all have intracellular tyrosine kinase domains and play a role in regulating these important processes [36]. It is known that VEGF-D signaling is particularly important for lymphangiogenesis [37]. Serum VEGF-D levels have been measured in several cohorts of sporadic LAM patients with a variety of comparison groups (summarized in Table S4). Because of the related molecular defect in sporadic LAM and TSC [38] and recent evidence that VEGF-D may be involved in the pathogenesis of sporadic LAM [39], [40], [41], [42], we measured serum VEGF-D levels in our participants. We found that baseline VEGF-D levels were elevated and decreased significantly after 52 weeks of sirolimus treatment (Figure 4). The mean baseline VEGF-D level in our study population (n = 28) was 7,827±10,080 pg/ml (95% CI 3,916–11,740). At week 52, the mean VEGF-D level was 2,215±4,319 pg/ml (95% CI 540–3,890, p<0.0001 using the Wilcoxon signed rank test). The two subjects with an increase in VEGF-D at week 52 had stable disease; they were both males so PFT data is not available. We observed higher VEGF-D levels in women (9,696±10,985 pg/ml) compared with men (2,217±2,546 mg/ml, p = 0.026 using the Mann-Whitney test). We also observed higher VEGF-D levels in TSC/LAM (10,070±10,946 pg/ml) compared with TSC (2,219±4,098 pg/ml, p = 0.004). We looked for and did not find a correlation between VEGF-D levels and PFTs (FVC, FEV1, DLCO) in the subset with TSC/LAM. Although there was no correlation between baseline PFTs (or change in PFTs) and VEGF-D levels, we did observe a correlation between VEGF-D levels and kidney angiomyolipoma size at baseline (Spearman correlation coefficient 0.56. p = 0.0020) and week 52 (Spearman correlation coefficient 0.43, p = 0.022), Figure 4C, D.

## Discussion

The major objectives of this single arm, multicenter, phase 2 study were to evaluate kidney angiomyolipoma response to and tolerability of sirolimus treatment. Overall, we found that sirolimus is relatively safe and may be a useful systemic option for treating patients with problematic multifocal kidney angiomyolipomas and other TSC related tumors. We observed that responses persisted at 24 months in a subset treated with additional sirolimus during year 2 of the study. We also observed regression of liver angiomyolipomas and SEGAs. Furthermore, serum VEGF-D levels were elevated at baseline, decreased with sirolimus treatment, and correlated with kidney angiomyolipoma size (for a comparison of our VEGF-D results to several sporadic LAM studies, see Table S4).

There are two other published kidney angiomyolipoma clinical trial studies so we compared the kidney angiomyolipoma response rate and toxicity data from our study (36 enrolled, 28 completed 1 year of sirolimus treatment) with the two related studies [31], [43]. One is a single institution sirolimus study (25 enrolled, 20 completed 1 year of sirolimus treatment) and the other is a UK multicenter sirolimus trial (16 enrolled, 10 completed 2 years of sirolimus treatment). Both our study and the UK study used RECIST criteria. We observed an overall response rate of 16/36 (44.4%) and the mean decrease in the sum LD for kidney tumors size was 29.9% with 1 year of treatment. In the UK study, the response rate was 8/16 (50%) with 2 years of treatment, and the mean decrease in the sum LD for kidney tumors was 25% at the 1 year time point. The single institution study used volumetric data to evaluate tumor regression and found that the mean reduction in volume was 53.2% with 1 year of treatment. Since RECIST uses a one dimensional measurement (sum LD) and volume is a three dimensional measurement, it is important to note that a 25–29% decrease by RECIST corresponds to a ∼59–64% decrease in volume if we assume tumors are approximately spherical. All three studies showed that kidney tumor regression was observed in all study subjects who completed 1 year of treatment, although in some cases tumor regression did not meet criteria for response by RECIST (which requires ≥30% decrease in sum LD). Kidney tumor regrowth was observed at 24 months in the subset of participants in this study who were followed off therapy after week 52 (n = 15) and in most of the single institution cohort (n = 18, all of whom were followed off therapy after week 52). Kidney tumor responses tended to persist in a subset of our study participants who were treated with additional sirolimus from months 12 to 24 (n = 13), and in all of the UK study participants who completed 24 months on study (n = 10). There were also many similarities regarding toxicity findings across all three studies. Serious drug related events were relatively rare (3 in our study, 6 in the single institution study, 3 in the UK study). However, mild and moderate toxicities were common and included mouth ulcers, hyperlipidemia, infections, proteinuria, and diarrhea. Additionally, our SEGA results are similar to the SEGA regression observed in a recently published single institution study of another mTOR inhibitor (everolimus) that showed a mean SEGA volume decrease of 49% at 12 months and 57% at 24 months) [44].

Progressive LAM is a devastating disorder with a known defect in the mTOR pathway [38], [45]. Both published kidney angiomyolipoma sirolimus treatment trials also collected pulmonary data so we compared our PFT results to those studies. Looking at the data from individual subjects, in our study we found that after 52 weeks of sirolimus treatment, FVC increased by more than 5% in 5/15 (33%) TSC/LAM subjects (3 moderate LAM, 2 mild LAM) and FEV1 increased by more than 5% in 4/15 (27%) TSC/LAM subjects (2 moderate LAM, 2 mild LAM), see Tables S7, S8 and Figures S5, S6, S7. In the UK multicenter study, FVC decreased by 76±52 ml/year and FEV1 decreased by 55±94 ml/year (n = 5 subjects with PFT results over 2 years). The single institution study reported week 52 PFT data on 11 cases with LAM (5 sporadic LAM and 6 with TSC/LAM) and noted a higher frequency of improvement in PFTs. In that study, FVC increased by more than 5% in 8/11 (73%) and FEV1 increased by more than 5% in 5/11 (45%). A major limitation in all of these studies is that the number of patients included is low, there is no untreated control group, pulmonary function testing is effort dependent, and there were differences in the LAM population included in the three studies. Furthermore, it appears that the single institution study may have included subjects with more severe lung disease as the average FEV1 and FVC at baseline were lower in their cohort than in ours and the UK study. Since we observed a tendency for improvement in the more severely affected group, this could be one explanation for the more dramatic findings in the single institution study. More recently, a randomized phase 3 trial of sirolimus for the treatment of LAM showed that the sirolimus group had significant improvement in FEV1 and FVC after 1 year of sirolimus treatment (n = 46 enrolled) compared to the placebo group (n = 43 enrolled). In this phase 3 study, subjects with more severe LAM were enrolled (FEV1 of <70% of predicted was required for study entry) and mean FEV1 remained stable in the sirolimus group (change from baseline to 12 months was 19±24 ml) compared with the placebo group (−134±182 ml, p = <0.001). Additionally, mean FVC showed some improvement in the sirolimus group (97±260 ml) compared with the placebo group (−129±233 ml, p = 0.001) [46]. These numbers are quite similar to our findings that in subjects with LAM (n = 15), the mean FEV1 change from baseline to week 52 was 20 ml and the mean FVC change from baseline to week 52 was 130 ml. A limitation of all LAM studies completed to date is that the treatment period was relatively short (12 months) so longer term studies are now needed. Although several studies now show improved pulmonary function with sirolimus treatment, it is not clear that that long term benefits will outweigh risks, especially considering that sirolimus is an immunosuppressant and pneumonitis is a known and potentially serious toxicity of mTOR inhibitors [32], [47]. Ideally the benefits and risks of using of sirolimus for the treatment of LAM will be further evaluated in longer term studies.

To our knowledge, this is the first clinical trial to report VEGF-D results in a TSC population with kidney angiomyolipomas before and after mTOR inhibitor treatment. The correlation between VEGF-D and kidney tumor size suggests that serum VEGF-D levels may be useful for monitoring kidney angiomyolipoma size over time. Our observations that VEGF-D levels are elevated at baseline and decrease with sirolimus treatment in a population with kidney angiomyolipomas associated with kidney disease are consistent with the VEGF-D results reported in the phase 3 sirolimus trial for women with LAM reported recently [46]. The clinical application of using VEGF-D levels to monitor kidney angiomyolipomas or other features of TSC and/or LAM will need confirmation in future studies. The positive results indicate that the inclusion of VEGF-D and other biomarker studies in future trials may yield new tools for clinical management and contribute to the understanding of disease pathology for TSC and/or LAM.

The numerous clinical features of TSC are well known [6], [33], [48] and well represented in our study participants (Table 1). There are significant unmet medical needs in this population; involvement of multiple organs is common, and many with TSC are living with chronic disabilities. The data reported here indicate that sirolimus treatment is a promising new approach to treatment for problematic kidney angiomyolipomas, liver angiomyolipomas, and SEGAs. Strengths of this study include: it is a multicenter study so is less prone to selection bias than single institution studies, tumor response was observed in multiple organ systems (kidney, brain, liver, skin) and this is the first TSC trial to identify a potentially useful biomarker (VEGF-D) for kidney angiomyolipomas. Limitations of this study include: it is a single arm, open label study, the follow-up duration was only 2 years, pediatric subjects were not included, and skin lesion assessment was subjective. Future trials for TSC related tumors should include longer duration sirolimus studies and pediatric subjects. Because the severity of kidney angiomyolipomas and other manifestations of TSC are variable, randomized trials that include patient reported or quality of life outcomes will have the highest impact regarding guiding the optimal use of mTOR inhibitors for the treatment of TSC. There is preclinical [17] and case report data [49] showing that lower sirolimus doses may be equally effective in treating TSC related kidney tumors. Therefore, trials using lower maintenance doses to see if the response is preserved while minimizing toxicity should also be considered. Brain manifestations (seizures, cognitive impairment, and behavioral problems) are a major source of morbidity for individuals with TSC. The improvement in cognition and seizures with sirolimus in mouse models [5], [16], [18], together with the efficacy and safety data reported here and other TSC mTOR inhibitor trials, indicate that clinical trials in children and adults with more severe manifestations of these features are now justified. Our skin lesion data, together with preclinical and case report data [50], [51], [52] indicate that studies evaluating systemic and/or topical sirolimus for treating TSC skin lesions are worthwhile. Our pulmonary function data suggest that longer term studies of pulmonary outcomes in women with progressive and/or moderate to severe LAM are of interest. As there are now several ongoing TSC and/or LAM trials investigating the utility of RAD001 (a rapamycin analog, also known as everolimus and Afinitor), it would also be useful to pursue comparative effectiveness studies in order to directly compare efficacy, safety, and costs of different mTOR inhibitors.

There are recent preclinical studies using mouse models of TSC related tumors that indicate other approved drugs (such as angiogenesis inhibitors, interferon-gamma, asparaginase) may have potential therapeutic utility as single agents or in combination with mTOR inhibitors [15], [17], [53], [54]. However, these studies show that non-mTOR inhibitors appear to be less effective than single agent mTOR inhibitors. Based on this, we suggest that clinical investigation of non-mTOR inhibitors as single agents or in combination with a mTOR inhibitors should be evaluated as second line therapy for problematic TSC related tumors that are not responding to an mTOR inhibitor. As the response of TSC related tumors in mouse models is consistent with response of TSC related tumors in humans, we anticipate that future preclinical studies have significant potential to identify other promising new medical approaches for the treatment of TSC and/or LAM.

This multicenter study should provide useful efficacy and safety data to clinicians who are considering the option of recommending sirolimus treatment for individuals with TSC and problematic kidney angiomyolipomas or other TSC associated tumors. Although there are alternative treatment options for problematic kidney angiomyolipomas (vascular embolization, nephrectomy, partial nephrectomy), these are all local, invasive procedures with associated risks. Furthermore, the available local interventions are often not suitable for the bilateral, multifocal kidney tumors that are frequently observed associated with TSC [25]. Ultimately it will be important to demonstrate that mTOR inhibitors can improve longer term outcomes in TSC and/or LAM patients with problematic kidney angiomyolipomas. Although there are challenges that slow the rate of clinical research progress for uncommon disorders [55], the quickest path to evaluating the benefits and risks of long term treatment is to pursue evaluation of mTOR inhibitors in larger numbers of affected individuals in the context of clinical studies that include substantial long term follow-up for both efficacy and safety endpoints.

## Supporting Information

Figure S1 **MR images of kidney angiomyolipoma before and after sirolimus treatment.** Axial T1-weighted fat-saturated post contrast MR images demonstrate an angiomyolipoma (arrow) in the upper pole of the left kidney measuring 2.81 cm maximal diameter at baseline, and 1.99 cm maximal diameter at week 52.
(DOC)

Figure S2 **MR images of kidney angiomyolipoma before and after sirolimus treatment.** Post contrast MR images demonstrate an angiomyolipoma (arrow) in the right kidney measuring 6.03 cm maximal diameter at baseline, and 3.91 cm maximal diameter at week 52.
(DOC)

Figure S3 **MR images of kidney angiomyolipoma before and after sirolimus treatment.** Post contrast MR images demonstrate an angiomyolipoma (arrow) in the right kidney measuring 3.62 cm maximal diameter at baseline, and 2.26 cm maximal diameter at week 52.
(DOC)

Figure S4 **MR images of SEGA (brain tumor) before and after sirolimus treatment.** T1 post gadolinium MR images demonstrate a SEGA that measured 2.03 cm at baseline and 1.68 cm at week 52.
(DOC)

Figure S5 **Pulmonary function: FVC before and after sirolimus treatment in LAM subgroups.**
(DOC)

Figure S6 **Pulmonary function: FEV1 before and after sirolimus treatment in LAM subgroups.**
(DOC)

Figure S7 **Pulmonary function: DLCO before and after sirolimus treatment in LAM subgroups.**
(DOC)

Text S1 **Serious adverse events unrelated to study drug and non-TSC related tumors.**
(DOC)

Text S2 **Tubers, SENs, and seizures before and after sirolimus treatment.**
(DOC)

Table S1 **Complete kidney angiomyolipoma response data.**
(DOC)

Table S2 **Complete toxicity data.**
(DOC)

Table S3 **Complete data on response of measurable liver angiomyolipomas.**
(DOC)

Table S4 **Summary of serum VEGF-D levels from this and other studies.** In this table we have summarized data from this study and all published VEGF-D data related to sporadic LAM. When all studies are compared, there are some similar trends. Four studies show that VEGF-D levels in sporadic LAM are greater than those in healthy controls [39], [40], [41], [42]. Our study and two others [41], [42] show that VEGF-D levels in TSC/LAM are greater than those in TSC (without LAM). Both our study and a phase 3 sirolimus LAM study [46] show that, in individuals with TSC and/or LAM, VEGF-D levels decrease with sirolimus treatment. One problem with comparing VEGF-D data across different studies is that results are not reported consistently. As shown in the table, studies have used different descriptive statistics (mean, median, or geometric mean) to report their findings.
(DOC)

Table S5 **TSC skin manifestations before and after sirolimus treatment.** These observations indicate that TSC related skin lesions seem to improve in some individuals after 52 weeks of sirolimus treatment. It is particularly encouraging that improvement was reported for facial angiofibromas in 57% of participants. Although the subjective nature of the assessment is a limitation of this data, these findings are consistent with a recent case report on the efficacy of systemic sirolimus for the treatment of TSC skin lesions [51]. Topical sirolimus is also of therapeutic interest because it has anti-tumor activity in a mouse model for TSC related tumors [52] and there is also a recent case report on the potential utility of topical sirolimus for the treatment of TSC skin lesions [50]. Overall, the exploratory results on TSC skin lesions indicate that additional clinical trials evaluating systemic or topical sirolimus for the treatment of TSC related skin disease could lead to new therapeutic options for these problems.
(DOC)

Table S6 **Complete pulmonary function data before and after sirolimus treatment.**
(DOC)

Table S7 **Summary of pulmonary function before and after sirolimus treatment in women with LAM.** Of the 18 subjects with baseline and week 52 PFT data, 15 had TSC/LAM (10-mild LAM, 5-moderate LAM), and 3 did not. For all 15 with TSC/LAM, on average there was a 4.3% increase in FVC, 0.9% increase in FEV1, and 0.9% decrease in DLCO when week 52 PFT results were compared with baseline data. If this group is divided into mild and moderate LAM subgroups, those in the moderate LAM subgroup (n = 5) had more evidence for improvement in FVC (8.9% increase) and FEV1 (3.4% increase) at week 52. In the group with moderate LAM, FVC increased from 3.50 to 3.81 L (p = 0.06, Wilcoxon signed rank test), FEV1 increased by 0.080 L during this study (from 2.38 L to 2.46 L, p value not significant), and DLCO was stable (13.69 ml/min/mm Hg at baseline and 13.91 ml/min/mm Hg at week 52). This is in contrast to epidemiology studies in which an annual decline in FEV1 of 0.06–0.1 L and decline in DLCO of 0.6–0.9 ml/min/mm Hg has been observed in cohorts with LAM [56], [57]. FVC and FEV1 remained close to baseline in the No LAM and Mild LAM groups, however a 7.0% decrease in DLCO was noted in the No LAM group at 52 weeks.
(DOC)

Table S8 **TSC gene mutations.** TSC gene mutation testing is now commercially available so we collected this data in our study subjects (see Table below). There were 18 cases that underwent mutation testing, 15 that were not tested, and 3 for whom testing status is unknown. In the 18 cases that were tested, 14 had *TSC2* gene mutations, 0 had *TSC1* mutations, and no mutation was identified in 4 cases. The *TSC2* mutation spectrum included 3 missense, 3 in frame deletions, 1 frame shift deletion, 3 nonsense, 1 splice, 1 large deletion, 1 inversion, and 1 unknown mutation type. When we compare this data (14 *TSC2* mutations, 0 *TSC1* mutations, 3 with no mutations identified ) to previously published genotype-phenotype data it appears that there may be a slightly higher frequency of *TSC2* mutations in the participants of this study (14/18, 78%) compared with the TSC populations from two genotype-phenotype studies where the frequency of *TSC2* mutations was 66–70% [6], [33]. There were a number of subjects for whom testing was not done (15/36, 42%), which suggests that mutaton testing is not considered critical and while mutaton testing can be very helpful for genetic counseling purposes, the expense may be a barrier for many, and testing is not considered critical for management decisions in this group.
(DOC)

Table S9 **Kidney cysts before and after sirolimus treatment.** There were 22/36 (61%) participants with renal cysts at study entry. To document the severity of kidney cysts associated with TSC, we graded these on a scale from 0 to 4 as follows: grade 0-no cysts, grade 1-up to 2 small cysts (all <2 cm), grade 2-more than 2 small cysts (all <2 cm), grade 3-more than 2 cysts with at least one >2 cm, grade 4-classic polycystic kidney disease. In our cohort, of the 22 with kidney cysts, grade 3 was most common (n = 9), followed by grade 2 (n = 6), grade 1 (n = 5), and grade 4 (n = 2). At week 52 on study, renal cysts data was available in 27 cases with the following results: grade 0 (n = 11), grade 1 (n = 3), grade 2 (n = 5), grade 3 (n = 7), grade 4 (n = 1). The table below lists the data for the 27 cases where kidney cyst data was available at both study entry and week 52. Although most (19 cases) had no change in kidney cyst grade with sirolimus treatment, there were 6 with a change in kidney cyst grade at week 52. It is interesting to note that kidney cyst grade increased in 3 cases and decreased in 3 cases. Although our data is limited because of the small numbers, overall our findings indicate that sirolimus treatment does not result in major changes to cystic kidney disease associated with TSC.
(DOC)

Table S10 **Kidney function before and after sirolimus treatment.** Almost all those enrolled had good renal function with a normal creatinine and BUN at baseline. There were only 6 with a creatinine ≥1.5 mg/dL at study entry. The average creatinine at study entry was 1.10±0.40 mg/dL (range 0.7–2.6, n = 36). The average blood urea nitrogen (BUN) at study entry was 17.75±10.70 mg/dL (range 8–70, n = 36). Sirolimus was not nephrotoxic in this population. Based on creatinine and BUN data at study entry and week 52, there was no significant difference after 52 weeks of drug treatment. For the 28 participants who completed 52 weeks of treatment, the average creatinine was 1.01±0.27 mg/dL at week 0 and 1.01±0.33 mg/dL at week 52. Similarly, the average BUN was 15.82±5.70 mg/dL at week 0 and 15.48±5.80 mg/dL at week 52 (see details in table below).
(DOC)

Table S11 **Summary of proteinuria data.** At study entry, proteinuria was not common but was noted in 4 subjects (31 with no proteinuria, 1-unknown). Of the 4 with proteinuria, according to urine dipstick testing, there were 2 with trace to 1+ proteinuria, 1 with 2+ proteinuria, and 1 with 3+ proteinuria. We did observe an increased frequency of proteinuria with sirolimus treatment, but in most cases proteinuria was trace to 1+. More severe (3+) proteinuria was less common but was observed in 2/28 (7.1%) subjects at week 52. In one case, the 3+ proteinuria prompted a sirolimus dose reduction and treatment with lisinopril. The data is summarized in the table below. There were 2 cases that had no proteinuria at baseline, but had 2+ or 3+ proteinuria at week 52. There were also 2 cases that had 2+ to 3+ proteinuria at baseline but had improvement at the week 104 visit. At week 52 there were 10 cases with proteinuria, at week 78 there were 6 cases with proteinuria, and at week 104 there were 11 cases with proteinuria. Almost all of these cases had only mild (trace to 1+) proteinuria. Of the 11 cases with trace to 1+ proteinuria at week 104, 7 were taking sirolimus according to the amended version of the protocol. Our data shows that an increased frequency of mild proteinuria may be a side effect of sirolimus treatment in this study population, which is consistent with the known toxicities of sirolimus in the kidney transplant population (Rapamune product information). It is important to note that severe proteinuria was infrequent and in 2 cases proteinuria improved during sirolimus treatment.
(DOC)

Table S12 **Summary of hematuria data.** Hematuria was not common, but was observed in 4 participants at study entry. In all 4 cases, baseline hematuria was minimal (up to 20 RBCs/high powered field (hpf)). As shown in the table below, at each time point there were 3–4 participants with minimal to mild hematuria (up to 10,000 RBCs/hpf). There were no cases of moderate/severe hematuria (>10,000 RBCs/hpf) at any time during this study. Interestingly, minimal to mild hematuria seemed to come and go in different participants over time. There were a total of 14 subjects who had minimal to mild hematuria at one of these time points (but not others) and 1 subject who had mild hematuria at 2 time points. Hematuria was clinically insignificant, remained infrequent, and generally did not persist so does not appear to be related to treatment with sirolimus.
(DOC)

Table S13 **Complete list of protocol deviations/violations.**
(DOC)

Protocol S1 **Trial Protocol.** Original protocol v011805.
(DOC)

Protocol S2 **Final protocol version v013009.**
(DOC)

Checklist S1 **CONSORT** checklist.
(DOC)
