# Supplementary figures and images for: Multicenter Phase 2 Trial of Sirolimus for Tuberous Sclerosis: Kidney Angiomyolipomas and Other Tumors Regress and VEGF- D Levels Decrease
Source: PLoS One. 2011 Sep 6;6(9):e23379. doi: 10.1371/journal.pone.0023379 (PMC3167813; doi:10.1371/journal.pone.0023379)

**
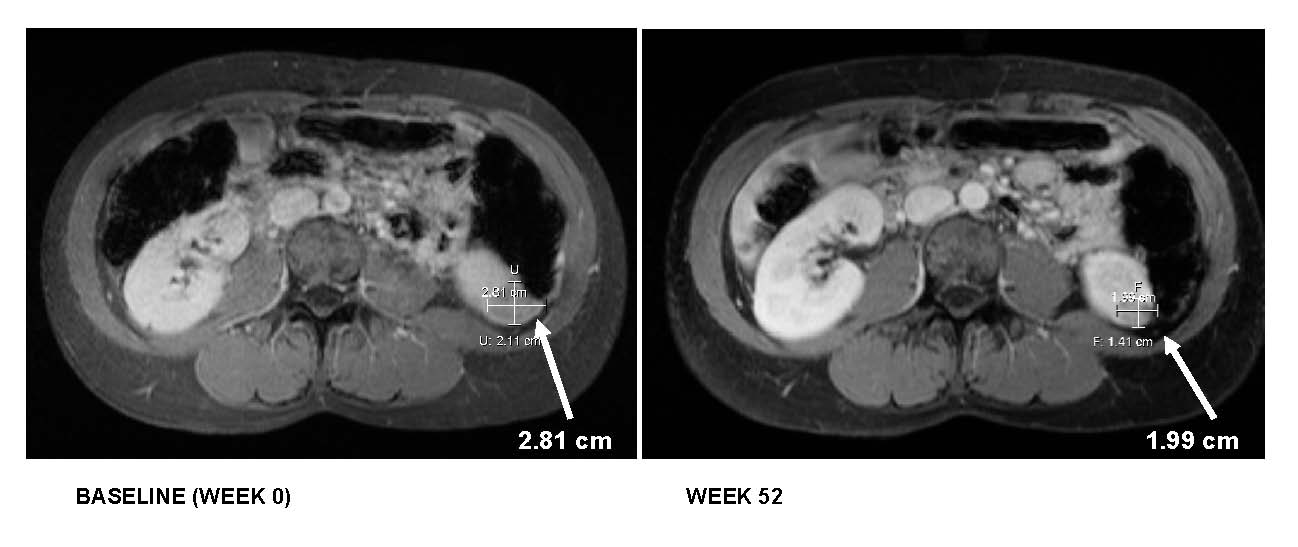
**

Supplement: Figure S1 — MR images of kidney angiomyolipoma before and after sirolimus treatment. Axial T1-weighted fat-saturated post contrast MR images demonstrate an angiomyolipoma (arrow) in the upper pole of the left kidney measuring 2.81 cm maximal diameter at baseline, and 1.99 cm maximal diameter at week 52. (DOC) [file pone.0023379.s001.doc]

**
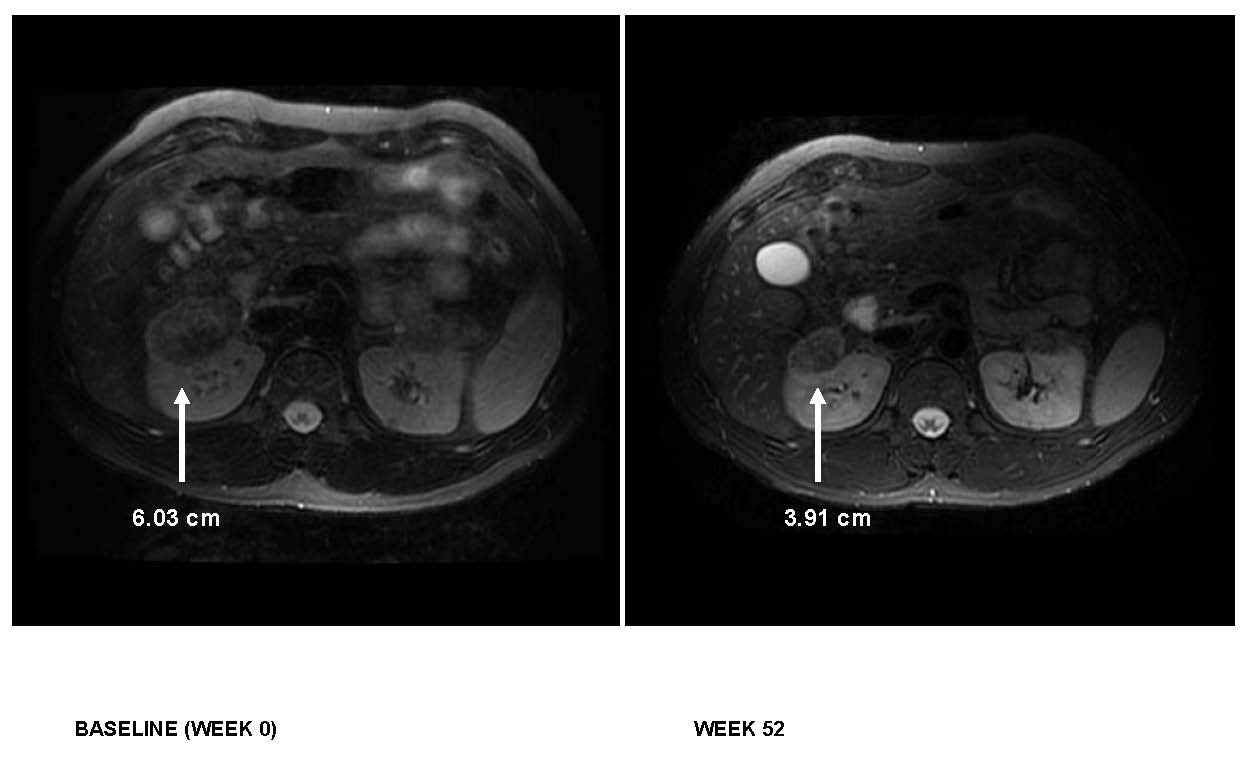
**

Supplement: Figure S2 — MR images of kidney angiomyolipoma before and after sirolimus treatment. Post contrast MR images demonstrate an angiomyolipoma (arrow) in the right kidney measuring 6.03 cm maximal diameter at baseline, and 3.91 cm maximal diameter at week 52. (DOC) [file pone.0023379.s002.doc]

**
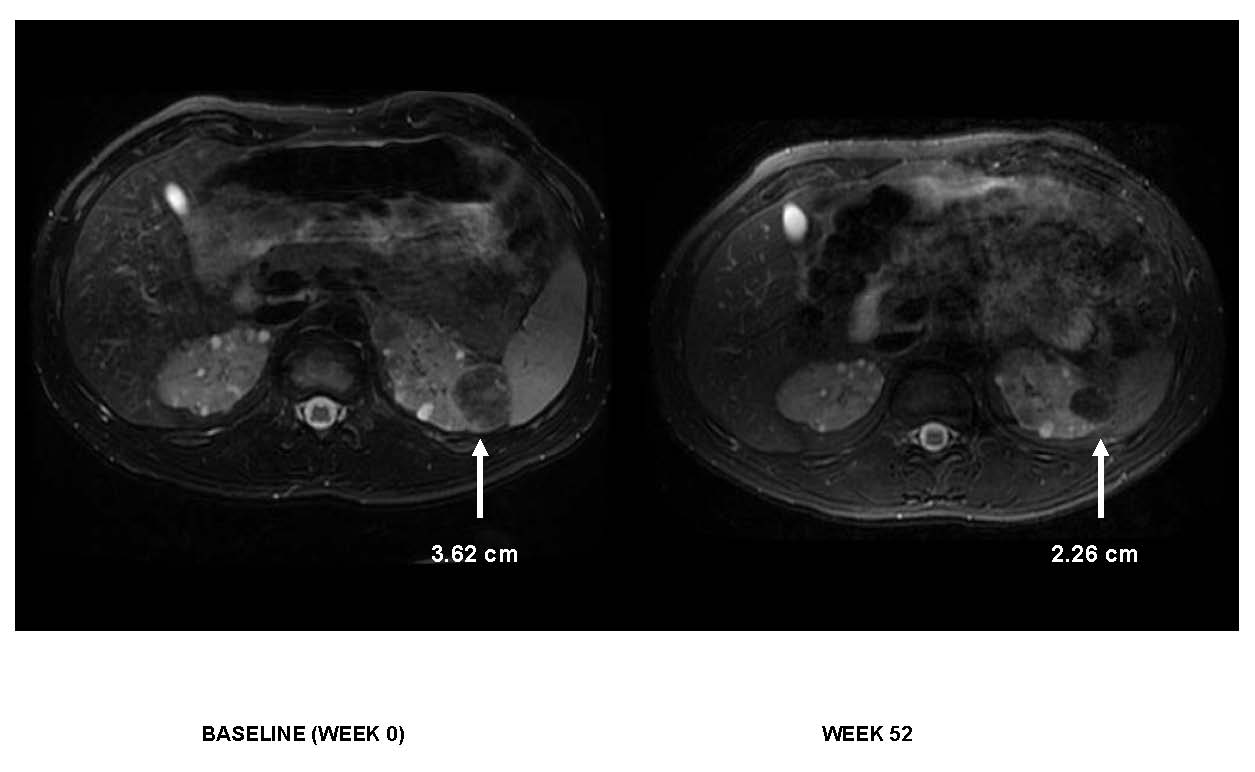
**

Supplement: Figure S3 — MR images of kidney angiomyolipoma before and after sirolimus treatment. Post contrast MR images demonstrate an angiomyolipoma (arrow) in the right kidney measuring 3.62 cm maximal diameter at baseline, and 2.26 cm maximal diameter at week 52. (DOC) [file pone.0023379.s003.doc]

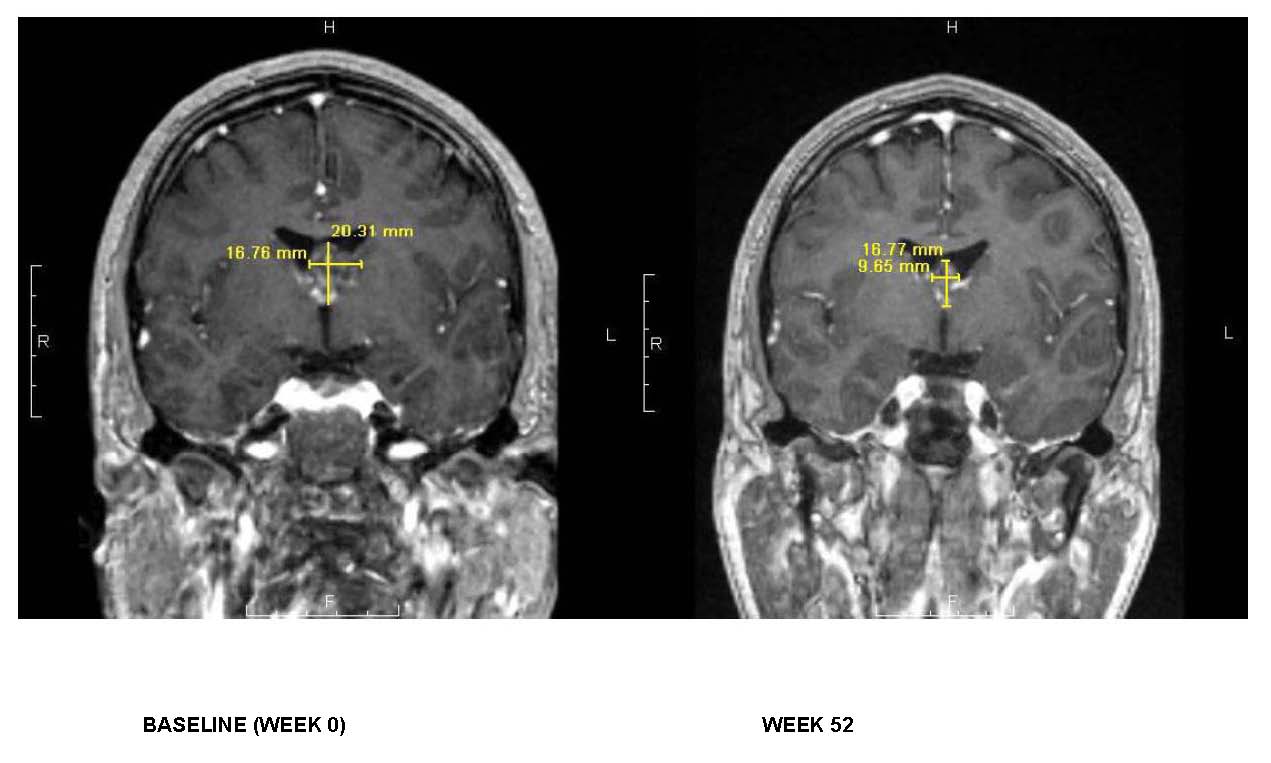

Supplement: Figure S4 — MR images of SEGA (brain tumor) before and after sirolimus treatment. T1 post gadolinium MR images demonstrate a SEGA that measured 2.03 cm at baseline and 1.68 cm at week 52. (DOC) [file pone.0023379.s004.doc]

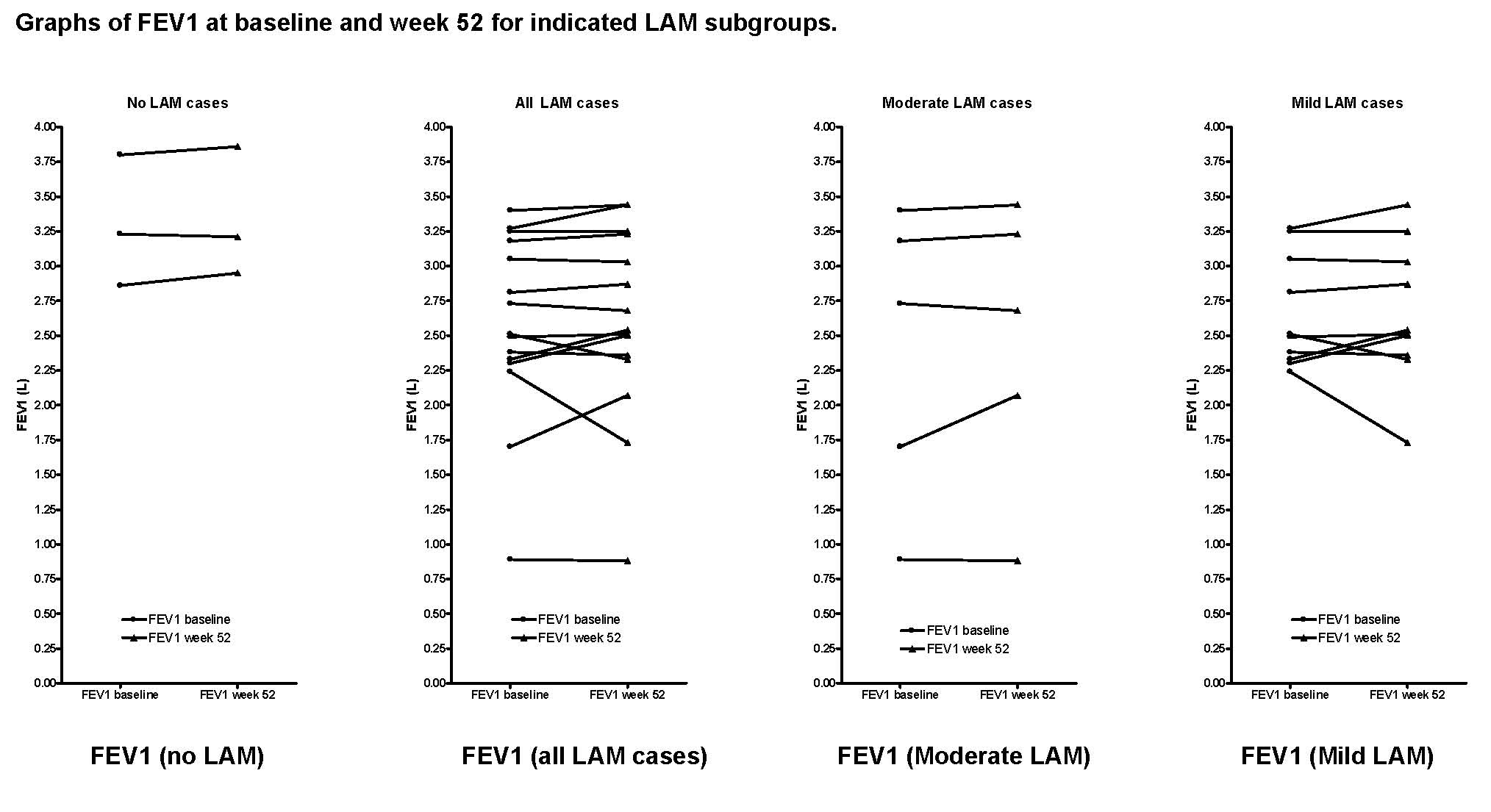

Supplement: Figure S6 — Pulmonary function: FEV1 before and after sirolimus treatment in LAM subgroups. (DOC) [file pone.0023379.s006.doc]

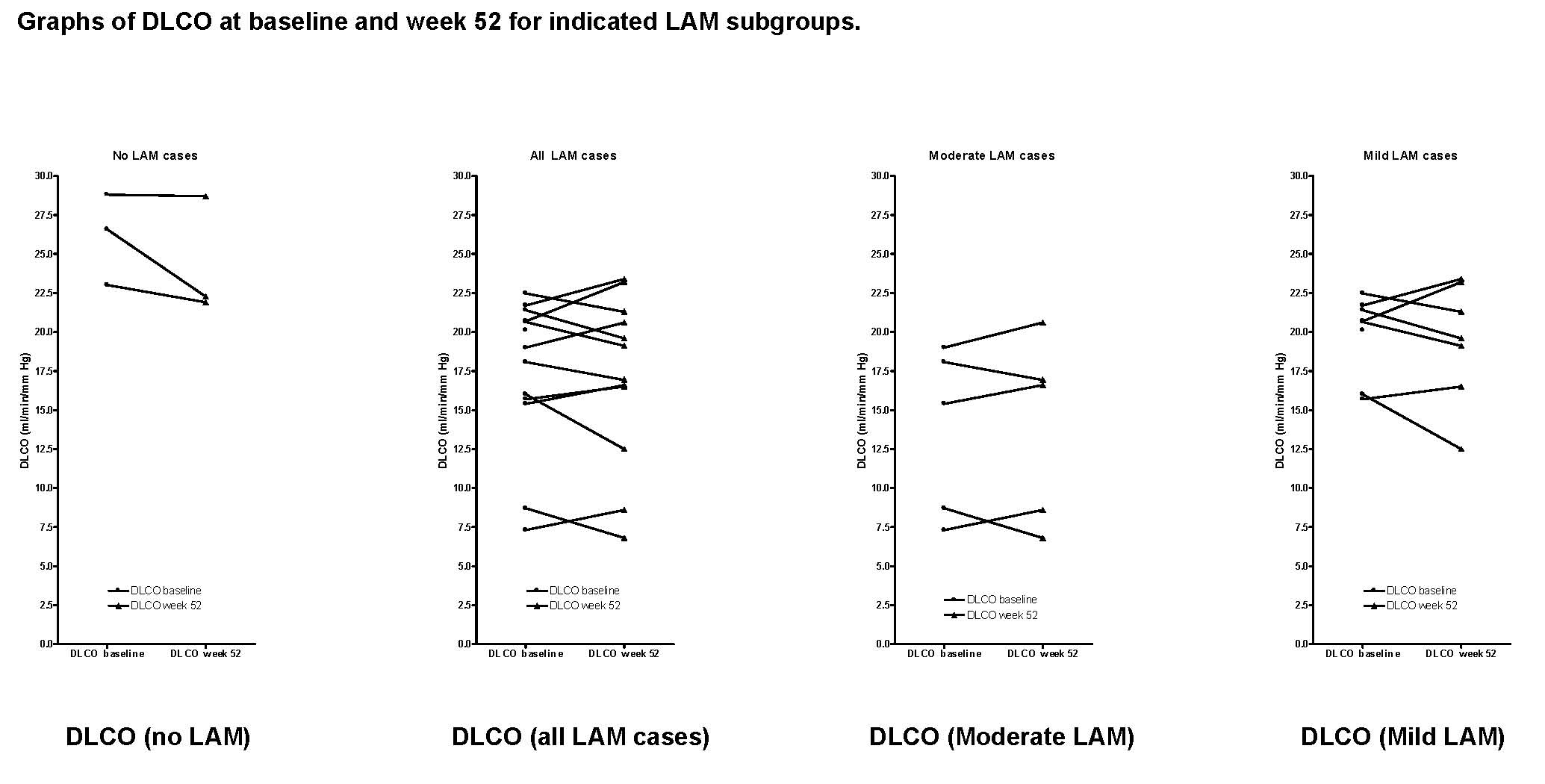

Supplement: Figure S7 — Pulmonary function: DLCO before and after sirolimus treatment in LAM subgroups. (DOC) [file pone.0023379.s007.doc]
