# Supplementary material for: Multicenter Phase 2 Trial of Sirolimus for Tuberous Sclerosis: Kidney Angiomyolipomas and Other Tumors Regress and VEGF- D Levels Decrease
Source: PLoS One. 2011 Sep 6;6(9):e23379. doi: 10.1371/journal.pone.0023379 (PMC3167813; doi:10.1371/journal.pone.0023379)
Supplement: Text S1 — Serious adverse events unrelated to study drug and non-TSC related tumors. (DOC) [file pone.0023379.s008.doc]

**Text S1. Serious adverse events unrelated to study drug and non-TSC related tumors**

**Serious adverse events unrelated to study drug.**

A total of seven serious adverse events were filed during the course of this study. These events included: 1 hospitalization for complicated infectious mononucleosis, 1 hospitalization for a neurologic non-epileptic event/pseudoseizure (this required intubation for airway protection after multiple doses of benzodiazepenes were given), 1 grade 3 seizure, 1 grade 3 kidney hemorrhage, 1 grade 3 lymphopenia (same case as mentioned above), 1 grade 3 pancreatic neuroendocrine tumor, and 1 hospitalization for new onset seizures. Only one of these (lymphopenia) was judged to be related to sirolimus treatment. All of these patients recovered from these events. The pancreatic neuroendocrine tumor was identified at the week 32 visit, but upon review of the baseline scan it was apparent at that time as well. The kidney angiomyolipoma hemorrhage occurred in the setting of a large >15cm tumor at a time when the participant was off sirolimus because of recent oral surgery. The neurologic event occurred in a participant with a prior history of non-epileptic events and was similar to the prior events. As shown in Table S2, there were also several grade 3 events that were judged unrelated to sirolimus treatment (1-abdominal pain, 1-dehydration, 1-limb pain, 1-gait/walking problem, 1-tooth injury, 1-joint pain, 1-soft tissue problem, 1-nausea, 1-reproductive function issue, 2-seizures, 1-vomiting).

**Non-TSC related tumors.**

At study entry or during the course of the study, there were four non-TSC related tumors in our cohort. There were 2 that were present at baseline: 1 subject had a known islet cell tumor (glucagonoma) of the pancreas and 1 subject was diagnosed with a pancreatic neuroendocrine tumor. The subject with the islet cell tumor/glucagonoma completed all 24 months on study and the glucagonoma remained stable to improved during this time. The pancreatic endocrine tumor was diagnosed at ~week 32 on study, but after careful re-review of the baseline MRI, it was also evident at study entry. In this case, study drug was stopped 19 days prior to surgical resection and not restarted because of delays in recovery following surgery. There were 2 participants in which tumors were diagnosed during year 2 on study. One subject was diagnosed with a giant cell tumor of the proximal tibia at 24 months. She developed left knee pain at ~18 months on study and a lytic bone lesion was noted at that time, followed without intervention for several months, and biopsied at ~month 24 on study. Another subject was diagnosed with early stage (node negative) breast cancer at 18 months on study. We are not aware that any of these tumor types are known to be associated with either TSC or sirolimus treatment.
