# Supplementary material for: Multicenter Phase 2 Trial of Sirolimus for Tuberous Sclerosis: Kidney Angiomyolipomas and Other Tumors Regress and VEGF- D Levels Decrease
Source: PLoS One. 2011 Sep 6;6(9):e23379. doi: 10.1371/journal.pone.0023379 (PMC3167813; doi:10.1371/journal.pone.0023379)
Supplement: Text S2 — Tubers, SENs, and seizures before and after sirolimus treatment. (DOC) [file pone.0023379.s009.doc]

**Text S2. Tubers, SENs, and seizures before and after sirolimus treatment**

At baseline, tuber data was available for 34 participants and 30 of these had tubers. Tubers were minimal (1-2 tubers) in 3 cases, mild (3-5 tubers) in 3 cases, moderate (6-10 tubers) in 11 cases, and severe (11 or more) in 13 cases. In most cases (24/26) with baseline and week 52 data, there were no changes noted in tubers with sirolimus treatment. At baseline, SEN data was available for 34 participants and most participants (30/34) had at least 1 SEN. The mean number of SENs at baseline was 3.4 ± 5.7 (range 0 to 8). At week 52, SEN data was available for 27 cases and mean number of SENs was 3.7 ± 5.7 (range 0 to 8). In most cases (26/27) with baseline and week 52 SEN data, there were no changes in SEN number with sirolimus treatment. Regarding seizures, clinical site investigators were asked to document their subjective assessment of changes in seizures after 52 weeks of sirolimus treatment. Data is available for 28 participants as follows: no seizures at baseline-17 cases, no change in seizures-5 cases, seizures worse-1 case, seizures improved-3 cases, unknown-2 cases. This pilot data indicates that dramatic changes in tubers, SENS, or seizures were not evident in this study. As refractory seizures are a major source of morbidity associated with TSC and there were 3 cases with subjective improvement noted (out of 11 cases with seizures at baseline), the impact of sirolimus treatment on seizure outcome should be further investigated in future clinical studies with relevant quantitative endpoints.
