# Supplementary material for: Multicenter Phase 2 Trial of Sirolimus for Tuberous Sclerosis: Kidney Angiomyolipomas and Other Tumors Regress and VEGF- D Levels Decrease
Source: PLoS One. 2011 Sep 6;6(9):e23379. doi: 10.1371/journal.pone.0023379 (PMC3167813; doi:10.1371/journal.pone.0023379)
Supplement: Table S1 — Complete kidney angiomyolipoma response data. (DOC) [file pone.0023379.s010.doc]

**Table S1. Complete kidney angiomyolipoma response data**

|  |  | Number of | Tumor size** | Tumor size |  |  | Tumor size |  |  | Tumor size |  |  | Sirolimus | Tumor size |  |  | Tumor size |  |  |
| --- | --- | --- | --- | --- | --- | --- | --- | --- | --- | --- | --- | --- | --- | --- | --- | --- | --- | --- | --- |
|  | Tumor | Target | Sum LD | Sum LD | % change |  | Sum LD | % change |  | Sum LD | % change |  | after week 52 | Sum LD | % change |  | Sum LD | % change |  |
| Subject # | Site | Lesions | (cm) | (cm) | vs baseline | Response* | (cm) | vs baseline | Response* | (cm) | vs baseline | Response* | (total weeks of | (cm) | vs baseline | Response* | (cm) | vs baseline | Response* |
|  |  |  |  |  |  |  |  |  |  |  |  |  | study drug) |  |  |  |  |  |  |
|  |  |  | Week 0 |  | Week 16 |  |  | Week 32 |  |  | Week 52 |  |  |  | 18 mos/Week 78 |  |  | 24 mos/Week 104 |  |
| 1 | Kidney | 5 | 26.0 | - | - | - | - | - | - | - | - | - | - | - | - | - | - | - | - |
| 2 | Kidney | 2 | 4.9 | 4.4 | -10.20% | SD | 3.4 | -30.6% | PR | - | - | - | - | - | - | - | - | - | - |
| 3 | Kidney | 5 | 23.7 | 21.5 | -9.28% | SD | 22.5 | -5.1% | SD | 22.7 | -4.2% | SD | No (52) | 25.1 | 5.9% | SD | 26.4 | 11.4% | SD |
| 4 | Kidney | 2 | 5.0 | 3.5 | -30.00% | PR | 3.3 | -34.0% | PR | 3.2 | -36.0% | PR | No (52) | 4.7 | -6.0% | SD | 5.0 | 0.0% | SD |
| 5 | Kidney | 5 | 25.5 | 22.4 | -12.16% | SD | 21.9 | -14.1% | SD | - | - | - | - | - | - | - | - | - | - |
| 6 | Kidney | 1 | 2.2 | 3.2 | 45.45% | PD | 2.9 | 31.8% | PD | 1.8 | -18.2% | SD | No (52) | 3.5 | 59.1% | PD | 3.5 | 59.1% | PD |
| 7 | Kidney | 2 | 5.6 | 5.5 | -1.79% | SD | - | - | - | - | - | - | - | - | - | - | - | - | - |
| 8 | Kidney | 1 | 4.1 | 3.1 | -24.39% | SD | 2.9 | -29.3% | SD | 2.5 | -39.0% | PR | No (52) | 4.0 | -2.4% | SD | 3.9 | -4.9% | SD |
| 9 | Kidney | 5 | 16.9 | 14.3 | -15.38% | SD | 11.1 | -34.3% | PR | 9.4 | -44.4% | PR | No (52) | 14.9 | -11.8% | SD | 10.9 | -35.5% | PR |
| 10 | Kidney | 3 | 48.8 | 50.2 | 2.87% | SD | - | - | - | - | - | - | - | - | - | - | - | - | - |
| 11 | Kidney | 4 | 41.6 | 42.1 | 1.20% | SD | - | - | - | - | - | - | - | - | - | - | - | - | - |
| 12 | Kidney | 3 | 17.0 | 12.3 | -27.65% | SD | 13.4 | -21.2% | SD | 12.5 | -26.5% | SD | No (52) | 15.8 | -7.1% | SD | 16.6 | -2.4% | SD |
| 13 | Kidney | 5 | 13.3 | 8.2 | -38.35% | PR | - | - | - | 7.8 | -41.4% | PR | No (52) | 12.1 | -9.0% | SD | 12.2 | -8.3% | SD |
| 14 | Kidney | 3 | 12.6 | 10.8 | -14.29% | SD | 11.8 | -6.3% | SD | 11.0 | -12.7% | SD | No (52) | 12.8 | 1.6% | SD | 11.4 | -9.5% | SD |
| 15 | Kidney | 1 | 5.8 | 5.5 | -5.17% | SD | 5.4 | -6.9% | SD | 5.4 | -6.9% | SD | No (52) | 5.0 | -13.8% | SD | 4.6 | -20.7% | SD |
| 16 | Kidney | 9 | 46.4 | 37.9 | -18.32% | SD | 36.2 | -22.0% | SD | 34.8 | -25.0% | SD | Yes (75) | 44.1 | -5.0% | SD | 36.0 | -22.4% | SD |
| 17 | Kidney | 5 | 46.6 | 34.1 | -26.82% | SD | 36.1 | -22.5% | SD | 38.6 | -17.2% | SD | No (52) | 46.0 | -1.3% | SD | 46.9 | 0.6% | SD |
| 18 | Kidney | 2 | 5.9 | 5.1 | -13.56% | SD | 4.3 | -27.1% | SD | 4.1 | -30.5% | PR | No (52) | 5.7 | -3.4% | SD | 5.7 | -3.4% | SD |
| 19 | Kidney | 5 | 28.1 | 25.5 | -9.25% | SD | 21.3 | -24.2% | SD | 23.5 | -16.4% | SD | Yes (69) | 27.7 | -1.4% | SD | 23.7 | -15.7% | SD |
| 20 | Kidney | 5 | 22.5 | 19.6 | -12.89% | SD | 19.8 | -12.0% | SD | 19.3 | -14.2% | SD | No (52) | 22.5 | 0.0% | SD | 23.9 | 6.2% | SD |
| 21 | Kidney | 3 | 15.1 | 13.3 | -11.92% | SD | 10.2 | -32.5% | PR | 13.3 | -11.9% | SD | No (52) | 17.0 | 12.6% | SD | 18.1 | 19.9% | SD |
| 22 | Kidney | 3 | 9.4 | 8.5 | -9.57% | SD | 7.7 | -18.1% | SD | 8.1 | -13.8% | SD | No (52) | 8.6 | -8.5% | SD | 9.4 | 0.0% | SD |
| 23 | Kidney | 3 | 12.7 | - | - | - | - | - | - | - | - | - | - | - | - | - | - | - | - |
| 24 | Kidney | 10 | 46.2 | 37.7 | -18.40% | SD | 35.6 | -22.9% | SD | 37.5 | -18.8% | SD | Yes (60) | 43.2 | -6.5% | SD | 44.9 | -2.8% | SD |
| 25 | Kidney | 8 | 23.5 | 16.6 | -29.36% | SD | 16.6 | -29.4% | SD | 15.1 | -35.7% | PR | Yes (88) | 15.6 | -33.6% | PR | 14.9 | -36.6% | PR |
| 26 | Kidney | 1 | 2.0 | 1.1 | -45.00% | PR | 1.1 | -45.0% | PR | 1.1 | -45.0% | PR | Yes (78) | 1.7 | -15.0% | SD | 1.0 | -50.0% | PR |
| 27 | Kidney | 4 | 13.8 | - | - | - | - | - | - | - | - | - | - | - | - | - | - | - | - |
| 28 | Kidney | 7 | 36.8 | 33.9 | -7.88% | SD | 34.5 | -6.2% | SD | 32.6 | -11.4% | SD | No (52) | 36.1 | -1.9% | SD | 38.4 | 4.3% | SD |
| 29 | Kidney | 4 | 26.1 | 20.0 | -23.37% | SD | 16.2 | -37.9% | PR | 17.2 | -34.1% | PR | Yes (78) | 26.7 | 2.3% | SD | 19.6 | -24.9% | SD |
| 30 | Kidney | 3 | 10.9 | 7.7 | -29.36% | SD | 7.5 | -31.2% | PR | 7.6 | -30.3% | PR | Yes (84) | 8.9 | -18.3% | SD | 7.7 | -29.4% | SD |
| 31 | Kidney | 4 | 26.9 | 21.7 | -19.33% | SD | 16.3 | -39.4% | PR | 13.0 | -51.7% | PR | Yes (74) | 24.8 | -7.8% | SD | 22.0 | -18.2% | SD |
| 32 | Kidney | 2 | 14.4 | 11.7 | -18.75% | SD | 10.9 | -24.3% | SD | 5.6 | -61.1% | PR | Yes (69) | 9.2 | -36.1% | PR | 9.3 | -35.4% | PR |
| 33 | Kidney | 5 | 31.8 | 28.5 | -10.38% | SD | 30.4 | -4.4% | SD | 30.6 | -3.8% | SD | Yes (84) | 30.8 | -3.1% | SD | 31.2 | -1.9% | SD |
| 34 | Kidney | 4 | 21.0 | 16.9 | -19.52% | SD | 11.4 | -45.7% | PR | 7.7 | -63.3% | PR | Yes (75) | 5.0 | -76.2% | PR | 5.8 | -72.4% | PR |
| 35 | Kidney | 3 | 18.9 | 11.5 | -39.15% | PR | 9.6 | -49.2% | PR | 11.6 | -38.6% | PR | Yes (78) | 17.7 | -6.3% | SD | 9.0 | -52.4% | PR |
| 36 | Kidney | 1 | 51.8 | 23.2 | -55.21% | PR | 11.9 | -77.0% | PR | 8.0 | -84.6% | PR | Yes (100) | 10.8 | -79.2% | PR | 10.6 | -79.5% | PR |
|  |  |  |  |  |  |  |  |  |  |  |  |  |  |  |  |  |  |  |  |
| ALL SUBJECTS, mean | | 3.8 | 21.2 | 17.6 | -16.9% |  | 15.0 | -24.9% |  | 14.5 | -29.9% |  |  |  |  |  |  |  |  |
| ± standard deviation | | ± 2.2 | ± 14.6 | ± 12.8 | ± 17.2% |  | ± 10.9 | ± 19.2% |  | ± 11.3 | ± 19.6% |  |  |  |  |  |  |  |  |
| OFF SIROLIMUS AFTER WK 52, mean | | | 15.8 |  |  |  |  |  |  |  |  |  |  | 15.6 | 0.9% |  | 15.8 | 1.1% |  |
| ± standard deviation | | | ± 12.5 |  |  |  |  |  |  |  |  |  |  | ± 12.3 | ± 17.5% |  | ± 13.1 | ± 20.6% |  |
| ON SIROLIMUS AFTER WK 52, mean | | | 26.8 |  |  |  |  |  |  |  |  |  |  | 20.5 | -22.0% |  | 18.1 | -34.0% |  |
| ± standard deviation | | | ± 14.6 |  |  |  |  |  |  |  |  |  |  | ± 13.7 | ± 27.3% |  | ± 13.0 | ± 24.1% |  |
| * Response was determined by comparing sum LD to baseline (PR-partial response, SD-stable disease, PD-progressive disease) | | | | | | | | | | | |  |  |  |  |  |  |  |  |
| ** Larger kidney angiomyolipomas (≥4cm) are at higher risk of bleeding. We reviewed our data for these higher risk lesions and found that 25/36 (69%) of subjects had at least 1 kidney tumor that was ≥4cm. In those that completed 52 weeks of treatment, 5 of these cases had a reduction to <4cm in all kidney angiomyolipomas. Considering individual lesions, there were a total of 65 kidney angiomyolipomas ≥4cm and 28 (43%) of these decreased to <4cm during sirolimus treatment. | | | | | | | | | | | | | | | | | | | |
