# Supplementary material for: Multicenter Phase 2 Trial of Sirolimus for Tuberous Sclerosis: Kidney Angiomyolipomas and Other Tumors Regress and VEGF- D Levels Decrease
Source: PLoS One. 2011 Sep 6;6(9):e23379. doi: 10.1371/journal.pone.0023379 (PMC3167813; doi:10.1371/journal.pone.0023379)
Supplement: Table S2 — Complete toxicity data. (DOC) [file pone.0023379.s011.doc]

**Table S2. Complete toxicity data**

|  |  | **ALL EVENTS INCLUDING UNRELATED TOXICITIES** | | | | **TREATMENT RELATED EVENTS** | | | |  |
| --- | --- | --- | --- | --- | --- | --- | --- | --- | --- | --- |
|  | **All Events (36 patients)** | **Grade 1** | **Grade 2** | **Grade 3** | **All Grades (1-3)** | **Grade 1** | **Grade 2** | **Grade 3** | **All Grades (1-3)** |  |
|  | Abdomen, pain | 1 | 1 | 1 | 3 |  |  |  |  |  |
|  | Alkaline phosphatase | 7 | 1 | 0 | 8 | 4 | 0 | 0 | 4 | * |
|  | Allergic rhinitis | 0 | 1 | 0 | 1 |  |  |  |  |  |
|  | ALT, SGPT | 4 | 1 | 0 | 5 | 3 | 1 | 0 | 4 | * |
|  | Anxiety | 0 | 1 | 0 | 1 |  |  |  |  |  |
|  | AST, SGOT | 3 | 0 | 0 | 3 | 1 | 0 | 0 | 1 |  |
|  | Back, pain | 1 | 0 | 0 | 1 |  |  |  |  |  |
|  | Breast, pain | 1 | 0 | 0 | 1 |  |  |  |  |  |
|  | Buttock, pain | 1 | 0 | 0 | 1 |  |  |  |  |  |
|  | Chest wall, pain | 1 | 1 | 0 | 2 |  |  |  |  |  |
|  | Cognitive disturbance | 1 | 0 | 0 | 1 |  |  |  |  |  |
|  | Cough | 5 | 0 | 0 | 5 | 1 | 0 | 0 | 1 |  |
|  | Creatinine | 4 | 0 | 0 | 4 | 2 | 0 | 0 | 2 |  |
|  | Dehydration | 0 | 0 | 1 | 1 |  |  |  |  |  |
|  | Dental/teeth/peridontal, pain | 1 | 0 | 0 | 1 |  |  |  |  |  |
|  | Diarrhea w/o prior colostomy | 10 | 1 | 0 | 11 | 3 | 1 | 0 | 4 | * |
|  | Distention/bloating, abdominal | 1 | 0 | 0 | 1 |  |  |  |  |  |
|  | Dizziness | 1 | 3 | 0 | 4 |  |  |  |  |  |
|  | Dry skin | 1 | 0 | 0 | 1 |  |  |  |  |  |
|  | Dyspepsia | 2 | 0 | 0 | 2 | 2 | 0 | 0 | 2 |  |
|  | Dyspnea | 0 | 2 | 0 | 2 |  |  |  |  |  |
|  | Edema limb | 0 | 1 | 0 | 1 | 0 | 1 | 0 | 1 |  |
|  | Edema trunk/genital | 1 | 0 | 0 | 1 |  |  |  |  |  |
|  | Edema visceral | 1 | 0 | 0 | 1 |  |  |  |  |  |
|  | External ear, pain | 0 | 1 | 0 | 1 |  |  |  |  |  |
|  | Extrapyramidal movement | 1 | 0 | 0 | 1 |  |  |  |  |  |
|  | Extremity-limb, pain | 2 | 0 | 1 | 3 |  |  |  |  |  |
|  | Extremity-lower (gait/walking) | 0 | 0 | 1 | 1 | 1 | 0 | 0 | 1 |  |
|  | Fatigue | 0 | 2 | 0 | 2 | 0 | 1 | 0 | 1 |  |
|  | Fever w/o neutropenia | 0 | 1 | 0 | 1 |  |  |  |  |  |
|  | Fracture | 1 | 0 | 0 | 1 |  |  |  |  |  |
|  | GI-other | 1 | 0 | 0 | 1 |  |  |  |  |  |
|  | Glomerular filtration rate | 1 | 0 | 0 | 1 | 1 | 0 | 0 | 1 |  |
|  | Head/headache | 7 | 1 | 1 | 9 | 3 | 1 | 1 | 5 | *# |
|  | Hematologic-other | 11 | 0 | 0 | 11 | 4 | 0 | 0 | 4 | * |
|  | Hemoglobin | 6 | 2 | 0 | 8 | 6 | 2 | 0 | 8 | ** |
|  | Hemorrhage-other | 1 | 0 | 0 | 1 |  |  |  |  |  |
|  | Hepatic-other | 0 | 1 | 0 | 1 |  |  |  |  |  |
|  | Hypercholesterolemia | 13 | 3 | 0 | 16 | 11 | 3 | 0 | 14 | ** |
|  | Hyperglycemia | 4 | 0 | 0 | 4 | 1 | 0 | 0 | 1 |  |
|  | Hyperkalemia | 2 | 1 | 0 | 3 |  |  |  |  |  |
|  | Hypertension | 0 | 1 | 0 | 1 | 0 | 1 | 0 | 1 |  |
|  | Hypertriglyceridemia | 12 | 8 | 0 | 20 | 10 | 8 | 0 | 18 | ** |
|  | Hypoalbuminemia | 2 | 0 | 0 | 2 | 1 | 0 | 0 | 1 |  |
|  | Hypocalcemia | 2 | 0 | 0 | 2 |  |  |  |  |  |
|  | Hypokalemia | 2 | 0 | 0 | 2 | 2 | 0 | 0 | 2 |  |
|  | Incontinence urinary | 1 | 0 | 0 | 1 |  |  |  |  |  |
|  | Infection Gr0-2 neut, bronchus | 0 | 1 | 0 | 1 | 0 | 1 | 0 | 1 |  |
|  | Infection Gr0-2 neut, larynx | 0 | 1 | 0 | 1 | 0 | 1 | 0 | 1 |  |
|  | Infection Gr0-2 neut, lung | 0 | 1 | 0 | 1 | 0 | 1 | 0 | 1 |  |
|  | Infection Gr0-2 neut, paranasal | 1 | 1 | 0 | 2 | 0 | 1 | 0 | 1 |  |
|  | Infection Gr0-2 neut, pharynx | 0 | 1 | 0 | 1 | 0 | 1 | 0 | 1 |  |
|  | Infection Gr0-2 neut, sinus | 1 | 1 | 0 | 2 | 1 | 1 | 0 | 2 |  |
|  | Infection Gr0-2 neut, skin | 0 | 2 | 0 | 2 | 0 | 1 | 0 | 1 |  |
|  | Infection Gr0-2 neut, soft tissue | 0 | 0 | 0 | 0 |  |  |  |  |  |
|  | Infection Gr0-2 neut, urinary tract | 6 | 2 | 0 | 8 | 4 | 2 | 0 | 6 | * |
|  | Infection w/ gr3-4 neut, upper airway | 1 | 0 | 0 | 1 |  |  |  |  |  |
|  | Infection w/ unk ANC lip/perioral | 1 | 0 | 0 | 1 | 1 | 0 | 0 | 1 |  |
|  | Infection w/ unk ANC pharynx | 1 | 0 | 0 | 1 | 1 | 0 | 0 | 1 |  |
|  | Infection w/ unk ANC sinus | 3 | 2 | 0 | 5 | 3 | 2 | 0 | 5 | * |
|  | Infection w/ unk ANC skin (cellulitis) | 2 | 0 | 0 | 2 | 1 | 0 | 0 | 1 |  |
|  | Infection w/ unk ANC upper airway NOS | 2 | 2 | 0 | 4 | 2 | 2 | 0 | 4 | * |
|  | Infection w/ unk ANC urinary tract NOS | 0 | 1 | 0 | 1 |  |  |  |  |  |
|  | Infection-other | 1 | 0 | 0 | 1 | 1 | 0 | 0 | 1 |  |
|  | Intra-op injury Teeth | 0 | 0 | 1 | 1 |  |  |  |  |  |
|  | Irregular menses | 5 | 1 | 0 | 6 | 5 | 0 | 0 | 5 | * |
|  | Joint, pain | 10 | 0 | 1 | 11 | 8 | 0 | 0 | 8 | ** |
|  | Kidney, pain | 0 | 2 | 0 | 2 |  |  |  |  |  |
|  | Larynx, pain | 1 | 0 | 0 | 1 |  |  |  |  |  |
|  | Leukocytes | 15 | 3 | 0 | 18 | 11 | 3 | 0 | 14 | ** |
|  | Lymphatics-other | 1 | 0 | 0 | 1 |  |  |  |  |  |
|  | Lymphopenia | 4 | 0 | 1 | 5 | 2 | 0 | 1 | 3 | # |
|  | Metabolic/Laboratory-other | 8 | 0 | 0 | 8 | 4 | 0 | 0 | 4 | * |
|  | Muco/stomatitis (symptom) oral cavity | 1 | 0 | 0 | 1 |  |  |  |  |  |
|  | Muco/stomatitis by exam, oral cavity | 2 | 0 | 0 | 2 |  |  |  |  |  |
|  | Musculoskeletal/soft tissue-other | 1 | 0 | 1 | 2 |  |  |  |  |  |
|  | Nasal cavity/paranasal sinus reaction | 1 | 0 | 0 | 1 | 1 | 0 | 0 | 1 |  |
|  | Nausea | 3 | 0 | 1 | 4 | 1 | 0 | 0 | 1 |  |
|  | Necrosis, oral | 15 | 6 | 0 | 21 | 15 | 6 | 0 | 21 | *** |
|  | Neurologic-other | 4 | 0 | 1 | 5 | 2 | 0 | 0 | 2 |  |
|  | Neuropathy-sensory | 0 | 1 | 0 | 1 |  |  |  |  |  |
|  | Neutrophils | 10 | 3 | 0 | 13 | 6 | 2 | 0 | 8 | ** |
|  | Nose, hemorrhage | 6 | 0 | 0 | 6 | 5 | 0 | 0 | 5 | * |
|  | Pelvic, pain | 1 | 0 | 0 | 1 |  |  |  |  |  |
|  | Photosensitivity | 1 | 0 | 0 | 1 |  |  |  |  |  |
|  | Platelets | 5 | 0 | 0 | 5 | 2 | 0 | 0 | 2 |  |
|  | Pneumonitis/pulmonary infiltrates | 1 | 0 | 0 | 1 | 1 | 0 | 0 | 1 |  |
|  | Proteinuria | 12 | 3 | 0 | 15 | 7 | 3 | 0 | 10 | ** |
|  | Pruritus/itching | 1 | 0 | 0 | 1 | 1 | 0 | 0 | 1 |  |
|  | Pulmonary/Upper Respiratory-other | 3 | 1 | 0 | 4 | 1 | 1 | 0 | 2 |  |
|  | Rash: acne/acneiform | 3 | 2 | 0 | 5 | 2 | 2 | 0 | 4 | * |
|  | Rash/desquamation | 4 | 1 | 0 | 5 | 2 | 0 | 0 | 2 |  |
|  | Renal/GU-other | 4 | 1 | 0 | 5 | 0 | 1 | 0 | 1 |  |
|  | Seizure | 2 | 4 | 2 | 8 |  |  |  | 0 |  |
|  | Sexual/Reproductive function-Other | 1 | 0 | 1 | 2 | 1 | 0 | 0 | 1 |  |
|  | Skin-other | 5 | 0 | 0 | 5 | 4 | 0 | 0 | 4 | * |
|  | Stomach, pain | 1 | 0 | 0 | 1 | 1 | 0 | 0 | 1 |  |
|  | Sweating | 1 | 0 | 0 | 1 | 1 | 0 | 0 | 1 |  |
|  | Throat/pharynx/larynx, pain | 0 | 3 | 0 | 3 | 0 | 1 | 0 | 1 |  |
|  | Vascular-Other (Specify) | 1 | 0 | 0 | 1 |  |  |  | 0 |  |
|  | Vomiting | 3 | 1 | 1 | 5 |  |  |  | 0 |  |
|  | Weight gain | 1 | 0 | 1 | 2 | 0 | 0 | 1 | 1 | # |
|  | Weight loss | 0 | 1 | 0 | 1 |  |  |  |  |  |
|  | Wound - non-infectious | 0 | 1 | 0 | 1 |  |  |  |  |  |
|  | **WORST DEGREE** | **4** | **24** | **8** | **36** | **6** | **26** | **3** | **35** |  |
|  |  |  |  |  |  |  |  |  |  |  |
| # | treatment related grade 3 event-headache, lymphopenia, weight gain | | |  |  |  |  |  |  |  |
| *** | treatment related occuring in >50% (19 or more subjects)-oral necrosis | | |  |  |  |  |  |  |  |
| ** | treatment related occuring in 20-50% (8-18 subjects)-hemoglobin, hypertriglyceridemia, hypercholesterolemia, leukocytes/neutrophils, proteinuria, joint pain | | | | | | | | |  |
| * | treatment related occuring in 10-20% (4-7 subjects)-alk phos, SGPT, diarrhea, headache, hematologic, UTI, sinus infection, pulmonary or upper resp. infection, irregular menses, metabolic/lab abnormalities, nose bleed, rash or other skin problems | | | | | | | | |  |
