# Supplementary material for: Multicenter Phase 2 Trial of Sirolimus for Tuberous Sclerosis: Kidney Angiomyolipomas and Other Tumors Regress and VEGF- D Levels Decrease
Source: PLoS One. 2011 Sep 6;6(9):e23379. doi: 10.1371/journal.pone.0023379 (PMC3167813; doi:10.1371/journal.pone.0023379)
Supplement: Table S3 — Complete data on response of measurable liver angiomyolipomas. (DOC) [file pone.0023379.s012.doc]

**Table S3. Complete data on response of measurable liver angiomyolipomas**

|  |  | Number of | Tumor size | Tumor size |  |  | Tumor size |  |  | Tumor size |  |  | Sirolimus | Tumor size |  |  | Tumor size |  |  |
| --- | --- | --- | --- | --- | --- | --- | --- | --- | --- | --- | --- | --- | --- | --- | --- | --- | --- | --- | --- |
|  | Tumor | Target | Sum LD | Sum LD | % change |  | Sum LD | % change |  | Sum LD | % change |  | after week 52 | Sum LD | % change |  | Sum LD | % change |  |
| Subject # | Site | Lesions | (cm) | (cm) | vs baseline | Response* | (cm) | vs baseline | Response* | (cm) | vs baseline | Response* | (total weeks of | (cm) | vs baseline | Response* | (cm) | vs baseline | Response* |
|  |  |  |  |  |  |  |  |  |  |  |  |  | study drug) |  |  |  |  |  |  |
|  |  |  | Week 0 |  | Week 16 |  |  | Week 32 |  |  | Week 52 |  |  |  | 18 mos/  Week 78 |  |  | 24 mos/  Week 104 |  |
| 6 | Liver | 1 | 4.2 | 3.9 | -7.1% |  | 3.6 | -14.3% |  | 3.6 | -14.3% |  |  | 4.8 | 14.3% |  | 4.7 | 11.9% |  |
| 6 | Retroperitoneal | 1 | 3.4 | 2.6 | -23.5% |  | 2.7 | -20.6% |  | 3.7 | 8.8% |  |  | 4.4 | 29.4% |  | 3.5 | 2.9% |  |
| 6 | All sites | 3 | 9.8 | 9.7 | -1.0% | SD | 9.2 | -6.1% | SD | 9.1 | -7.1% | SD | No (52) | 12.7 | 29.6% | PD | 11.7 | 19.4% | SD |
| 11 | Liver | 1 | 2.9 | 2.9 | 0.0% |  | - | - |  | - | - |  |  | - | - |  | - | - |  |
| 11 | All Sites |  | 44.5 | 45.0 | 1.1% | SD | - | - | - | - | - | - | No (< 32) | - | - | - | - | - | - |
| 18 | Liver | 1 | 3.5 | 3.5 | 0.0% |  | 3.2 | -8.6% |  | 3.4 | -2.9% |  |  | 3.2 | -8.6% |  | 3.6 | 2.9% |  |
| 18 | All sites | 3 | 9.4 | 8.6 | -8.5% | SD | 7.5 | -20.2% | SD | 7.5 | -20.2% | SD | No (52) | 8.9 | -5.3% | SD | 9.3 | -1.1% | SD |
| 35 | Liver | 1 | 5.9 | 3.0 | -49.2% |  | 3.8 | -35.6% |  | 3.4 | -42.4% |  |  | 3.5 | -40.7% |  | 1.6 | -72.9% |  |
| 35 | All Sites | 3 | 24.8 | 14.5 | -41.5% | PR | 13.4 | -46.0% | PR | 15.0 | -39.5% | PR | Yes (78) | 21.2 | -14.5% | SD | 10.6 | -57.3% | PR |
| 36 | Liver | 1 | 5.4 | 2.2 | -59.3% |  | 1.9 | -64.8% |  | 8.2 | 51.9% |  |  | 2.3 | -57.4% |  | 2.4 | -55.6% |  |
| 36 | All sites | 2 | 57.2 | 25.4 | -55.6% | PR | 13.8 | -75.9% | PR | 16.2 | -71.7% | PR | Yes (100) | 13.1 | -77.1% | PR | 13.0 | -77.3% | PR |
| * Response was determined by comparing sum LD to baseline | | | | | |  |  |  |  |  |  |  |  |  |  |  |  |  |  |
| PR-partial response, SD-stable disease, PD-progressive disease | | | | | |  |  |  |  |  |  |  |  |  |  |  |  |  |  |
