# Supplementary material for: Multicenter Phase 2 Trial of Sirolimus for Tuberous Sclerosis: Kidney Angiomyolipomas and Other Tumors Regress and VEGF- D Levels Decrease
Source: PLoS One. 2011 Sep 6;6(9):e23379. doi: 10.1371/journal.pone.0023379 (PMC3167813; doi:10.1371/journal.pone.0023379)
Supplement: Table S4 — Summary of serum VEGF-D levels from this and other studies. In this table we have summarized data from this study and all published VEGF-D data related to sporadic LAM. When all studies are compared, there are some similar trends. Four studies show that VEGF-D levels in sporadic LAM are greater than those in healthy controls [39], [40], [41], [42]. Our study and two others [41], [42] show that VEGF-D levels in TSC/LAM are greater than those in TSC (without LAM). Both our study and a phase 3 sirolimus LAM study [46] show that, in individuals with TSC and/or LAM, VEGF-D levels decrease with sirolimus treatment. One problem with comparing VEGF-D data across different studies is that results are not reported consistently. As shown in the table, studies have used different descriptive statistics (mean, median, or geometric mean) to report their findings. (DOC) [file pone.0023379.s013.doc]

| **Table S4. Summary of serum VEGF-D levels from this and other studies** | | | | | | |  |  |
| --- | --- | --- | --- | --- | --- | --- | --- | --- |
| **Group** | **N** | **VEGF-D (pg/ml)** | **VEGF-D (pg/ml)** | **VEGF-D (pg/ml)** | **Range** | **95% CI** | **95% CI** | **Reference** |
|  |  | **mean ± SD** | **median** | **geometric mean** |  | **(for mean)** | **(for geometric mean)** |  |
| **TSC (and TSC/LAM)** | **28** | **7827 ± 10085** | **3462** | **3388** | **282-40000** | **3916-11737** | **1966-5838** |  |
| **Females (with TSC or TSC/LAM)** | **21** | **9696 ± 10985** | **6689** | **4707** | **463-40000** | **4696-14697** | **2548-8695** | **This study,**  **baseline data** |
| **Males (with TSC or TSC/LAM)** | **7** | **2217 ± 2546** | **906** | **1263** | **282-6663** | **-137-4572** | **441-3613** |
| **TSC/LAM** | **20** | **10070 ± 10946** | **6676** | **5529** | **613-40000** | **4947-15192** | **3150-9703** |  |
| **TSC without LAM** | **8** | **2219 ± 4098** | **871** | **996** | **282-12328** | **-1206-5645** | **387-2562** |  |
| **Normal controls (all female)** | **24** |  |  | **296** |  |  | **263-333** | **Seyama et al., 2006** |
| **LAM** | **44** |  |  | **1069** |  |  | **809-1413** |
| **Normal controls (86% female)** | **29** | **~300** |  |  | **~100-800** |  |  |  |
| **Sporadic LAM** | **38** | **2000** |  |  | **250-10000** |  |  | **Young et al., 2008** |
| **TSC/LAM** | **17** | **6804** |  |  | **~1000-24000** | **3826-9781** |  |
| **TSC Only (Females)** | **12** | **491** |  |  | **~300-1200** | **291-691** |  |  |
| **TSC Only (Males)** | **14** | **~1000** |  |  | **~200-2500** |  |  |  |
|  |  | **mean ± SE** |  |  |  |  |  |  |
| **Sporadic LAM (all)** | **111** | **1869 ± 145** |  |  |  |  |  |  |
| **Healthy (all female)** | **40** | **657 ± 43** |  |  |  |  |  | **Glasgow et al., 2009** |
| **Sporadic LAM + LN** | **77** | **2273 ± 173** |  |  |  |  |  |
| **Sporadic LAM - LN** | **34** | **945 ± 186** |  |  |  |  |  |  |
| **Sporadic LAM + AMLs** | **40** | **1445 ± 209** |  |  |  |  |  |  |
| **Sporadic LAM - AMLs** | **71** | **2108 ± 186** |  |  |  |  |  |  |
| **Sporadic LAM** | **56** |  | **1175** |  |  |  |  |  |
| **Other cystic lung disease** | **44** |  | **281** |  |  |  |  | **Young et al., 2010** |
| **Healthy (all female)** | **40** |  | **~400** |  |  |  |  |
| **TSC/LAM** | **28** |  | **~4000** |  |  |  |  |  |
| **TSC** | **17** |  | **~400** |  |  |  |  |  |
| **LAM (91% sporadic, 9% with TSC)** | **89** | **2029 ± 2342** |  |  |  |  |  | **McCormack et al., 2011** |
| **SD-standard deviation** |  |  |  |  |  |  |  |  |
| **SE-standard error** |  |  |  |  |  |  |  |  |
| **LN-lymph nodes** |  |  |  |  |  |  |  |  |
