# Supplementary material for: Multicenter Phase 2 Trial of Sirolimus for Tuberous Sclerosis: Kidney Angiomyolipomas and Other Tumors Regress and VEGF- D Levels Decrease
Source: PLoS One. 2011 Sep 6;6(9):e23379. doi: 10.1371/journal.pone.0023379 (PMC3167813; doi:10.1371/journal.pone.0023379)
Supplement: Table S5 — TSC skin manifestations before and after sirolimus treatment. These observations indicate that TSC related skin lesions seem to improve in some individuals after 52 weeks of sirolimus treatment. It is particularly encouraging that improvement was reported for facial angiofibromas in 57% of participants. Although the subjective nature of the assessment is a limitation of this data, these findings are consistent with a recent case report on the efficacy of systemic sirolimus for the treatment of TSC skin lesions [51]. Topical sirolimus is also of therapeutic interest because it has anti-tumor activity in a mouse model for TSC related tumors [52] and there is also a recent case report on the potential utility of topical sirolimus for the treatment of TSC skin lesions [50]. Overall, the exploratory results on TSC skin lesions indicate that additional clinical trials evaluating systemic or topical sirolimus for the treatment of TSC related skin disease could lead to new therapeutic options for these problems. (DOC) [file pone.0023379.s014.doc]

| **Table S5. TSC skin manifestations before and after sirolimus treatment** | | | | |
| --- | --- | --- | --- | --- |
|  |  |  |  |  |
|  | Number (%) | Number (%) | Number (%) | Number (%) |
|  | With Skin Lesions | Improved | Unchanged | Not present at baseline  (or unknown) |
|  | On Study | Week 52 | Week 52 | Week 52 |
| Facial angiofibroma | 35/36 (97) | 16/28 (57) | 11/28 (39) | 1/28 (4)* |
| Hypomelanotic macules | 24/36 (67) | 5/28 (18) | 11/28 (39) | 12/28 (43)** |
| Shagreen patch | 22/36 (61) | 8/28 (29) | 13/28 (46) | 7/28 (25) |
| Ungual fibromas | 20/36 (56) | 8/28 (29) | 10/28 (36) | 10/28 (36) |
| Forehead plaque | 14/26 (39) | 6/28 (21) | 7/28 (25) | 15/28 (54) |
| *facial angiofibroma data unknown for 1 case | | |  |  |
| **hypomelanotic macule data unknown for 2 cases | | |  |  |
