# Supplementary material for: Multicenter Phase 2 Trial of Sirolimus for Tuberous Sclerosis: Kidney Angiomyolipomas and Other Tumors Regress and VEGF- D Levels Decrease
Source: PLoS One. 2011 Sep 6;6(9):e23379. doi: 10.1371/journal.pone.0023379 (PMC3167813; doi:10.1371/journal.pone.0023379)
Supplement: Table S6 — Complete pulmonary function data before and after sirolimus treatment. (DOC) [file pone.0023379.s015.doc]

Table S6. Complete pulmonary function data before and after sirolimus treatment

| Case number | LAM Severity* | FVC (L) | | | FVC |  | FEV1 (L) | | | FEV1 |  | DLCO (ml/min/mm Hg) | | | DLCO |
| --- | --- | --- | --- | --- | --- | --- | --- | --- | --- | --- | --- | --- | --- | --- | --- |
| (all female) |  | Baseline | Week 52 | change | %change |  | Baseline | Week 52 | change | %change |  | Baseline | Week 52 | change | %change |
| 1 | Mild | 3.03 |  |  |  |  | 2.62 |  |  |  |  | 15.28 |  |  |  |
| 4 | Mild | 2.94 | 2.74 | -0.2 | -6.8% |  | 2.51 | 2.33 | -0.18 | -7.2% |  | 15.69 | 16.51 | 0.82 | 5.2% |
| 6 | Moderate | 4.28 | 4.29 | 0.01 | 0.2% |  | 3.4 | 3.44 | 0.04 | 1.2% |  | 18.07 | 16.94 | -1.13 | -6.3% |
| 10 | Mild | 3.6 |  |  |  |  | 3.17 |  |  |  |  | 26.9 |  |  |  |
| 11 | Moderate | 2.19 |  |  |  |  | 1.85 |  |  |  |  | 11.81 |  |  |  |
| 14 | Mild | 2.85 | 2.84 | -0.01 | -0.4% |  | 2.49 | 2.51 | 0.02 | 0.8% |  | 20.11 | not avail |  |  |
| 15 | Absent | 4.08 | 4 | -0.08 | -2.0% |  | 3.8 | 3.86 | 0.06 | 1.6% |  | 26.58 | 22.28 | -4.3 | -16.2% |
| 16 | Absent | 3.7 | 3.68 | -0.02 | -0.5% |  | 3.23 | 3.21 | -0.02 | -0.6% |  | 28.8 | 28.7 | -0.1 | -0.3% |
| 17 | Mild | 3.34 | 3.48 | 0.14 | 4.2% |  | 2.81 | 2.87 | 0.06 | 2.1% |  | 20.69 | 23.19 | 2.5 | 12.1% |
| 18 | Mild | 2.3 | 2.71 | 0.41 | 17.8% |  | 2.3 | 2.5 | 0.2 | 8.7% |  | not avail | not avail |  |  |
| 21 | Mild | 3.06 | 3.07 | 0.01 | 0.3% |  | 2.38 | 2.36 | -0.02 | -0.8% |  | 20.65 | 19.11 | -1.54 | -7.5% |
| 22 | Mild | 4.14 | 4 | -0.14 | -3.4% |  | 3.25 | 3.25 | 0 | 0.0% |  | 22.48 | 21.3 | -1.18 | -5.2% |
| 23 | Absent | 2.19 |  |  |  |  | 2.08 |  |  |  |  |  |  |  |  |
| 24 | Mild | 3.49 | 3.47 | -0.02 | -0.6% |  | 3.05 | 3.03 | -0.02 | -0.7% |  | not avail | not avail |  |  |
| 25 | Moderate | 3.15 |  |  |  |  | 2.53 |  |  |  |  | 18.8 |  |  |  |
| 26 | Absent | 3.3 | 3.44 | 0.14 | 4.2% |  | 2.86 | 2.95 | 0.09 | 3.1% |  | 23 | 21.9 | -1.1 | -4.8% |
| 27 | Mild | 3.59 |  |  |  |  | 3.13 |  |  |  |  | 20.9 |  |  |  |
| 28 | Moderate | 3.43 | 3.5 | 0.07 | 2.0% |  | 2.73 | 2.68 | -0.05 | -1.8% |  | 18.99 | 20.61 | 1.62 | 8.5% |
| 29 | Moderate | 3.44 | 4.08 | 0.64 | 18.6% |  | 1.7 | 2.07 | 0.37 | 21.8% |  | 7.3 | 8.6 | 1.3 | 17.8% |
| 30 | Mild | 2.28 | 2 | -0.28 | -12.3% |  | 2.24 | 1.73 | -0.51 | -22.8% |  | 16 | 12.5 | -3.5 | -21.9% |
| 32 | Mild | 4.13 | 4.53 | 0.4 | 9.7% |  | 3.27 | 3.44 | 0.17 | 5.2% |  | 21.7 | 23.4 | 1.7 | 7.8% |
| 33 | Mild | 3.02 | 3.21 | 0.19 | 6.3% |  | 2.33 | 2.54 | 0.21 | 9.0% |  | 21.4 | 19.6 | -1.8 | -8.4% |
| 34 | Moderate | 2.25 | 2.46 | 0.21 | 9.3% |  | 0.89 | 0.88 | -0.01 | -1.1% |  | 8.7 | 6.8 | -1.9 | -21.8% |
| 35 | Moderate | 4.12 | 4.7 | 0.58 | 14.1% |  | 3.18 | 3.23 | 0.05 | 1.6% |  | 15.4 | 16.6 | 1.2 | 7.8% |
|  | *according to data reported with week 52 pulmonary function testing | | | | | | | |  |  |  |  |  |  |  |
