# Supplementary material for: Multicenter Phase 2 Trial of Sirolimus for Tuberous Sclerosis: Kidney Angiomyolipomas and Other Tumors Regress and VEGF- D Levels Decrease
Source: PLoS One. 2011 Sep 6;6(9):e23379. doi: 10.1371/journal.pone.0023379 (PMC3167813; doi:10.1371/journal.pone.0023379)
Supplement: Table S7 — Summary of pulmonary function before and after sirolimus treatment in women with LAM. Of the 18 subjects with baseline and week 52 PFT data, 15 had TSC/LAM (10-mild LAM, 5-moderate LAM), and 3 did not. For all 15 with TSC/LAM, on average there was a 4.3% increase in FVC, 0.9% increase in FEV1, and 0.9% decrease in DLCO when week 52 PFT results were compared with baseline data. If this group is divided into mild and moderate LAM subgroups, those in the moderate LAM subgroup (n = 5) had more evidence for improvement in FVC (8.9% increase) and FEV1 (3.4% increase) at week 52. In the group with moderate LAM, FVC increased from 3.50 to 3.81 L (p = 0.06, Wilcoxon signed rank test), FEV1 increased by 0.080 L during this study (from 2.38 L to 2.46 L, p value not significant), and DLCO was stable (13.69 ml/min/mm Hg at baseline and 13.91 ml/min/mm Hg at week 52). This is in contrast to epidemiology studies in which an annual decline in FEV1 of 0.06–0.1 L and decline in DLCO of 0.6–0.9 ml/min/mm Hg has been observed in cohorts with LAM [56], [57]. FVC and FEV1 remained close to baseline in the No LAM and Mild LAM groups, however a 7.0% decrease in DLCO was noted in the No LAM group at 52 weeks. (DOC) [file pone.0023379.s016.doc]

Table S7. Summary of pulmonary function before and after sirolimus treatment in women with LAM

| **LAM Severity** | **FVC (L)** | **FVC (L)** | **FVC (L)** | **FVC** |  | **FEV1 (L)** | **FEV1 (L)** | **FEV1 (L)** | **FEV1** |  | **DLCO (ml/min/mm Hg)***** | | | **DLCO** |  |
| --- | --- | --- | --- | --- | --- | --- | --- | --- | --- | --- | --- | --- | --- | --- | --- |
| **(n)** | **Week 0** | **Week 52** | **change** | **%change** | **Number** | **Week 0** | **Week 52** | **change** | **%change** | **Number** | **Week 0** | **Week 52** | **change** | **%change** | **Number** |
| Absent (n=3) | 3.69 | 3.71 | 0.01 | 0.5% | 3 | 3.30 | 3.34 | 0.04 | 1.3% | 3 | 26.13 | 24.29 | -1.84 | -7.0% | 3 |
| Mild or Moderate (n=15) | 3.27 | 3.41 | 0.13 | 4.3% | 15 | 2.57 | 2.59 | 0.02 | 0.9% | 15 | 17.26 | 17.10 | -0.16 | -0.9% | 12 |
| Mild (n=10) | 3.16 | 3.21 | 0.05 | 1.6% | 10 | 2.66 | 2.66 | 0.00 | 0.0% | 10 | 19.80 | 19.37 | -0.43 | -2.2% | 7 |
| Moderate (n=5) | 3.50** | 3.81** | 0.31 | 8.9% | 5 | 2.38 | 2.46 | 0.08 | 3.4% | 5 | 13.69 | 13.91 | 0.22 | 1.6% | 5 |
| *according to data reported with week 52 pulmonary function testing | | | | | |  |  |  |  |  |  |  |  |  |  |
| **p = 0.06 |  |  |  |  |  |  |  |  |  |  |  |  |  |  |  |
| ***DLCO data not available at week 52 in 3 cases | | | | |  |  |  |  |  |  |  |  |  |  |  |
