# Supplementary material for: Multicenter Phase 2 Trial of Sirolimus for Tuberous Sclerosis: Kidney Angiomyolipomas and Other Tumors Regress and VEGF- D Levels Decrease
Source: PLoS One. 2011 Sep 6;6(9):e23379. doi: 10.1371/journal.pone.0023379 (PMC3167813; doi:10.1371/journal.pone.0023379)
Supplement: Table S8 — TSC gene mutations. TSC gene mutation testing is now commercially available so we collected this data in our study subjects (see Table below). There were 18 cases that underwent mutation testing, 15 that were not tested, and 3 for whom testing status is unknown. In the 18 cases that were tested, 14 had TSC2 gene mutations, 0 had TSC1 mutations, and no mutation was identified in 4 cases. The TSC2 mutation spectrum included 3 missense, 3 in frame deletions, 1 frame shift deletion, 3 nonsense, 1 splice, 1 large deletion, 1 inversion, and 1 unknown mutation type. When we compare this data (14 TSC2 mutations, 0 TSC1 mutations, 3 with no mutations identified ) to previously published genotype-phenotype data it appears that there may be a slightly higher frequency of TSC2 mutations in the participants of this study (14/18, 78%) compared with the TSC populations from two genotype-phenotype studies where the frequency of TSC2 mutations was 66–70% [6], [33]. There were a number of subjects for whom testing was not done (15/36, 42%), which suggests that mutaton testing is not considered critical and while mutaton testing can be very helpful for genetic counseling purposes, the expense may be a barrier for many, and testing is not considered critical for management decisions in this group. (DOC) [file pone.0023379.s017.doc]

Table S8. TSC gene mutations

| Subject # | Gene | Exon/intron | Mutation | Mutation Type |
| --- | --- | --- | --- | --- |
| 1 | *TSC2* | unknown | unknown | unknown |
| 2 | Test ordered but no mutation identified |  |  |  |
| 3 | *TSC2* | Exon 5 | 487-488 del TT | deletion, frameshift |
| 4 | Test ordered but no mutation identified |  |  |  |
| 5 | *TSC2* | Exon 38 | 5056 C>T, Gln 1686>X | nonsense |
| 6 | *TSC2* | Exon 23 | 2713 C>T, 905Arg>Trp | missense |
| 7 | Test ordered but no mutation identified |  |  |  |
| 8 | *TSC2* | Exon 13 | 1372 C>T, 458 Arg>X | nonsense |
| 9 | *TSC2* | Exon 10 | 1093-1095 del ATC, 365del | deletion, in frame |
| 10 | Test ordered but no mutation identified |  |  |  |
| 11 | *TSC2* | Exon 40 | 5227-5244 18 BP deletion | deletion, in frame |
| 12 | Test not ordered |  |  |  |
| 13 | *TSC2* | Exons 1-41 (all) | large deletion NHL1-TSC2-PKD1 | large deletion |
| 14 | *TSC2* | Exon 37 | 4852-4860 9 BP deletion | deletion, in frame |
| 15 | *TSC2* | Exon 33 | 4289 G>A, 1420 Trp>X | nonsense, mosaic |
| 16 | Test not ordered |  |  |  |
| 17 | *TSC2* | Intron 38 | 5068+1 G>A | splice |
| 18 | *TSC2* | Exon 39 | 5126 C>T, 1709 Pro>Leu | missense |
| 19 | Test not ordered |  |  |  |
| 20 | Test not ordered |  |  |  |
| 21 | *TSC2* | unknown | inversion | inversion |
| 22 | *TSC2* | Exon 16 | 1831 C>T, 611 Arg>Trp | missense |
| 23 | Test not ordered |  |  |  |
| 24 | Test not ordered |  |  |  |
| 25 | Test not ordered |  |  |  |
| 26 | Unknown | unknown | unknown | unknown |
| 27 | Test not ordered |  |  |  |
| 28 | Unknown | unknown | unknown | unknown |
| 29 | Test not ordered |  |  |  |
| 30 | Test not ordered |  |  |  |
| 31 | Test not ordered |  |  |  |
| 32 | Test not ordered |  |  |  |
| 33 | Test not ordered |  |  |  |
| 34 | Test not ordered |  |  |  |
| 35 | Unknown | unknown | unknown | unknown |
| 36 | Test not ordered |  |  |  |
