# Supplementary material for: Multicenter Phase 2 Trial of Sirolimus for Tuberous Sclerosis: Kidney Angiomyolipomas and Other Tumors Regress and VEGF- D Levels Decrease
Source: PLoS One. 2011 Sep 6;6(9):e23379. doi: 10.1371/journal.pone.0023379 (PMC3167813; doi:10.1371/journal.pone.0023379)
Supplement: Table S9 — Kidney cysts before and after sirolimus treatment. There were 22/36 (61%) participants with renal cysts at study entry. To document the severity of kidney cysts associated with TSC, we graded these on a scale from 0 to 4 as follows: grade 0-no cysts, grade 1-up to 2 small cysts (all <2 cm), grade 2-more than 2 small cysts (all <2 cm), grade 3-more than 2 cysts with at least one >2 cm, grade 4-classic polycystic kidney disease. In our cohort, of the 22 with kidney cysts, grade 3 was most common (n = 9), followed by grade 2 (n = 6), grade 1 (n = 5), and grade 4 (n = 2). At week 52 on study, renal cysts data was available in 27 cases with the following results: grade 0 (n = 11), grade 1 (n = 3), grade 2 (n = 5), grade 3 (n = 7), grade 4 (n = 1). The table below lists the data for the 27 cases where kidney cyst data was available at both study entry and week 52. Although most (19 cases) had no change in kidney cyst grade with sirolimus treatment, there were 6 with a change in kidney cyst grade at week 52. It is interesting to note that kidney cyst grade increased in 3 cases and decreased in 3 cases. Although our data is limited because of the small numbers, overall our findings indicate that sirolimus treatment does not result in major changes to cystic kidney disease associated with TSC. (DOC) [file pone.0023379.s018.doc]

| **Table S9. Kidney cysts before and after sirolimus treatment** | | | | |
| --- | --- | --- | --- | --- |
|  | Kidney cyst grade | Kidney cyst grade |  | Kidney tumor response |
| Case | week 0 | week 52 | Change | at week 52 |
|  |  |  |  |  |
| 2 | 2 | 2 | no |  |
| 3 | 2 | 2 | no |  |
| 4 | 0 | 0 | no |  |
| 6 | 3 | 3 | no |  |
| 8 | 0 | 1 | yes, 0 to 1 | PR |
| 9 | 2 | 2 | no |  |
| 13 | 2 | 0 | yes, 2 to 0 | PR |
| 14 | 0 | 0 | no |  |
| 15 | 0 | 0 | no |  |
| 16 | 3 | 3 | no |  |
| 17 | 0 | 0 | no |  |
| 18 | 0 | 3 | yes, 0 to 3 | PR |
| 19 | 3 | 3 | no |  |
| 20 | 0 | 0 | no |  |
| 21 | 1 | 2 | yes, 1 to 2 | SD |
| 22 | 2 | 2 | no |  |
| 24 | 3 | 3 | no |  |
| 25 | 3 | 3 | no |  |
| 26 | 3 | 1 | yes, 3 to 1 | PR |
| 28 | 0 | 0 | no |  |
| 29 | 0 | 0 | no |  |
| 30 | 3 | 3 | no |  |
| 31 | 1 | 0 | no |  |
| 33 | 4 | 4 | no |  |
| 34 | 1 | 1 | no |  |
| 35 | 0 | 0 | no |  |
| 36 | 1 | 0 | yes, 1 to 0 | PR |
