# Supplementary material for: Multicenter Phase 2 Trial of Sirolimus for Tuberous Sclerosis: Kidney Angiomyolipomas and Other Tumors Regress and VEGF- D Levels Decrease
Source: PLoS One. 2011 Sep 6;6(9):e23379. doi: 10.1371/journal.pone.0023379 (PMC3167813; doi:10.1371/journal.pone.0023379)
Supplement: Table S10 — Kidney function before and after sirolimus treatment. Almost all those enrolled had good renal function with a normal creatinine and BUN at baseline. There were only 6 with a creatinine ≥1.5 mg/dL at study entry. The average creatinine at study entry was 1.10±0.40 mg/dL (range 0.7–2.6, n = 36). The average blood urea nitrogen (BUN) at study entry was 17.75±10.70 mg/dL (range 8–70, n = 36). Sirolimus was not nephrotoxic in this population. Based on creatinine and BUN data at study entry and week 52, there was no significant difference after 52 weeks of drug treatment. For the 28 participants who completed 52 weeks of treatment, the average creatinine was 1.01±0.27 mg/dL at week 0 and 1.01±0.33 mg/dL at week 52. Similarly, the average BUN was 15.82±5.70 mg/dL at week 0 and 15.48±5.80 mg/dL at week 52 (see details in table below). (DOC) [file pone.0023379.s019.doc]

| **Table S10. Kidney function before and after sirolimus treatment** | | | | |
| --- | --- | --- | --- | --- |
|  |  |  |  |  |
|  | Creatinine | Creatinine | BUN | BUN |
|  | (mg/dL) | (mg/dL) | (mg/dL) | (mg/dL) |
| Case # | Week 0 | Week 52 | Week 0 | Week 52 |
|  |  |  |  |  |
| 3 | 0.9 | 0.9 | 20 | 15 |
| 4 | 1.0 | 0.9 | 12 | 12 |
| 6 | 1.6 | 1.5 | 17 | 17 |
| 8 | 0.8 | 0.9 | 19 | 16 |
| 9 | 1.1 | 0.8 | 14 | 13 |
| 12 | 1.0 | 1.1 | 12 | 12 |
| 13 | 0.7 | 0.7 | 11 | 12 |
| 14 | 0.9 | 1.1 | 19 | 30 |
| 15 | 0.8 | 1.0 | 9 | 13 |
| 16 | 0.8 | 0.6 | 18 | 12 |
| 17 | 1.3 | 1.3 | 24 | 16 |
| 18 | 0.7 | 0.8 | 13 | 13 |
| 19 | 1.0 | 1.0 | 13 | 19 |
| 20 | 1.0 | 0.9 | 15 | 13 |
| 21 | 1.0 | 1.2 | 8 | 11 |
| 22 | 1.1 | 0.9 | 18 | 18 |
| 24 | 0.8 | 0.7 | 8 | 10 |
| 25 | 0.6 | 0.5 | 10 | 9 |
| 26 | 0.9 | 0.9 | 10 | 8 |
| 28 | 1.5 | 1.4 | 25 | 21 |
| 29 | 1.1 | 1.3 | 27 | 19 |
| 30 | 0.7 | 0.6 | 10 | 15 |
| 31 | 1.2 | 1.2 | 16 | 15 |
| 32 | 0.9 | 0.9 | 14 | 13 |
| 33 | 0.9 | 0.7 | 11 | 11 |
| 34 | 1.3 | 1.9 | 23 | 22 |
| 35 | 1.7 | 1.7 | 21 | 34 |
| 36 | 1.0 | 1.0* | 26 | 15 |
|  |  |  |  |  |
| Average, n=28 | 1.01 | 0.98 | 15.82 | 15.48 |
| SD | 0.27 | 0.38 | 5.70 | 5.80 |
|  |  |  |  |  |
| *Week 52 data not available so this is the average of week 32 and 78 | | | | |
| SD-standard deviation | |  |  |  |
