# Supplementary material for: Multicenter Phase 2 Trial of Sirolimus for Tuberous Sclerosis: Kidney Angiomyolipomas and Other Tumors Regress and VEGF- D Levels Decrease
Source: PLoS One. 2011 Sep 6;6(9):e23379. doi: 10.1371/journal.pone.0023379 (PMC3167813; doi:10.1371/journal.pone.0023379)
Supplement: Table S11 — Summary of proteinuria data. At study entry, proteinuria was not common but was noted in 4 subjects (31 with no proteinuria, 1-unknown). Of the 4 with proteinuria, according to urine dipstick testing, there were 2 with trace to 1+ proteinuria, 1 with 2+ proteinuria, and 1 with 3+ proteinuria. We did observe an increased frequency of proteinuria with sirolimus treatment, but in most cases proteinuria was trace to 1+. More severe (3+) proteinuria was less common but was observed in 2/28 (7.1%) subjects at week 52. In one case, the 3+ proteinuria prompted a sirolimus dose reduction and treatment with lisinopril. The data is summarized in the table below. There were 2 cases that had no proteinuria at baseline, but had 2+ or 3+ proteinuria at week 52. There were also 2 cases that had 2+ to 3+ proteinuria at baseline but had improvement at the week 104 visit. At week 52 there were 10 cases with proteinuria, at week 78 there were 6 cases with proteinuria, and at week 104 there were 11 cases with proteinuria. Almost all of these cases had only mild (trace to 1+) proteinuria. Of the 11 cases with trace to 1+ proteinuria at week 104, 7 were taking sirolimus according to the amended version of the protocol. Our data shows that an increased frequency of mild proteinuria may be a side effect of sirolimus treatment in this study population, which is consistent with the known toxicities of sirolimus in the kidney transplant population (Rapamune product information). It is important to note that severe proteinuria was infrequent and in 2 cases proteinuria improved during sirolimus treatment. (DOC) [file pone.0023379.s020.doc]

| **Table S11. Summary of proteinuria data** | | | | | | |  |  |
| --- | --- | --- | --- | --- | --- | --- | --- | --- |
|  |  |  |  |  |  |  |  |  |
| Proteinuria Severity | Baseline (n=36) | | Week 52 (n=28) | | Week 78 (n=28) | | Week 104 (n=28) | |
|  | n | % | n | % | n | % | n | % |
|  |  |  |  |  |  |  |  |  |
| None | 31 | 86.1% | 16 | 57.1% | 17 | 60.7% | 15 | 53.6% |
| Trace or 1+ | 2 | 5.6% | 7 | 25.0% | 6 | 21.4% | 11 | 39.3% |
| 2+ | 1 | 2.8% | 1 | 3.6% | 0 | 0.0% | 0 | 0.0% |
| 3+ | 1 | 2.8% | 2 | 7.1% | 0 | 0.0% | 0 | 0.0% |
| Unknown | 1 | 2.8% | 2 | 7.1% | 5 | 17.9% | 2 | 7.1% |
|  |  |  |  |  |  |  |  |  |
