# Supplementary material for: Multicenter Phase 2 Trial of Sirolimus for Tuberous Sclerosis: Kidney Angiomyolipomas and Other Tumors Regress and VEGF- D Levels Decrease
Source: PLoS One. 2011 Sep 6;6(9):e23379. doi: 10.1371/journal.pone.0023379 (PMC3167813; doi:10.1371/journal.pone.0023379)
Supplement: Table S12 — Summary of hematuria data. Hematuria was not common, but was observed in 4 participants at study entry. In all 4 cases, baseline hematuria was minimal (up to 20 RBCs/high powered field (hpf)). As shown in the table below, at each time point there were 3–4 participants with minimal to mild hematuria (up to 10,000 RBCs/hpf). There were no cases of moderate/severe hematuria (>10,000 RBCs/hpf) at any time during this study. Interestingly, minimal to mild hematuria seemed to come and go in different participants over time. There were a total of 14 subjects who had minimal to mild hematuria at one of these time points (but not others) and 1 subject who had mild hematuria at 2 time points. Hematuria was clinically insignificant, remained infrequent, and generally did not persist so does not appear to be related to treatment with sirolimus. (DOC) [file pone.0023379.s021.doc]

| **Table S12. Summary of Hematuria Data** | | | | | | | | |
| --- | --- | --- | --- | --- | --- | --- | --- | --- |
|  |  |  |  |  |  |  |  |  |
|  |  |  |  |  |  |  |  |  |
|  | Baseline (n=36) | | Week 52 (n=28) | | Week 78 (n=28) | | Week 104 (n=28) | |
|  | n | % | n | % | n | % | n | % |
|  |  |  |  |  |  |  |  |  |
| Negative for blood or No RBCs/hpf | 31 | 86.1% | 23 | 82.1% | 20 | 71.4% | 22 | 78.6% |
| Minimal (up to 20 RBCs/hpf) | 4 | 11.1% | 2 | 7.1% | 2 | 7.1% | 3 | 10.7% |
| Mild (20 to 10,000 RBCs/hpf) | 0 | 0.0% | 2 | 7.1% | 1 | 3.6% | 1 | 3.6% |
| Moderate/Severe (>10,000 RBCs/hpf) | 0 | 0.0% | 0 | 0.0% | 0 | 0.0% | 0 | 0.0% |
| Unknown | 1 | 2.8% | 1 | 3.6% | 5 | 17.9% | 2 | 7.1% |
|  |  |  |  |  |  |  |  |  |
|  |  |  |  |  |  |  |  |  |
