# Supplementary material for: Multicenter Phase 2 Trial of Sirolimus for Tuberous Sclerosis: Kidney Angiomyolipomas and Other Tumors Regress and VEGF- D Levels Decrease
Source: PLoS One. 2011 Sep 6;6(9):e23379. doi: 10.1371/journal.pone.0023379 (PMC3167813; doi:10.1371/journal.pone.0023379)
Supplement: Table S13 — Complete list of protocol deviations/violations. (DOC) [file pone.0023379.s022.doc]

**Table S13. Complete list of protocol deviations/violations**

Note: All of these deviations/violations were submitted to the Dana-Farber IRB and the IRB approved our corrective action plan in all cases.

| **Number** | **Date Reported to IRB** | **Description of deviation/violation reported to the Dana-Farber IRB** | **Category** |
| --- | --- | --- | --- |
| 1 | 3/1/2007 | The subject signed the specimen consent on 11/15/06, an invalid study form (no expiration date) was signed the same day. All baseline study related procedures were done on 11/15/06. The subject was asked to come back and signed a valid consent form on 11/17/06. | consent issue |
| 2 | 3/1/2007 | Invalid consent (missing approval and expiration dates) for rapamycin administration was signed and registered at the QACT on 10/31/06, rapamycin administration was started on 11/10/06. Valid consent was signed and sent to the QACT on 11/28/06. | consent issue |
| 3 | 3/1/2007 | Valid consent for collection of blood, urine and tissue specimens was taken on 10/31/06, questions (1), (2) and (3) were left blank. | consent issue |
| 4 | 3/1/2007 | The specimen consent and study forms were signed by the subject and co-signed by study nurse, who was not identified by the PI as someone who will be obtaining consent. The site PI was present during the consent procedures and did not sign the forms. | consent issue |
| 5 | 3/1/2007 | Eligibilty criteria states that platelets must be within normal limits. The subject had an elevated platelet count (486 th/cmm, normal range 150-350 th/cmm). Rapamycin can lower the platelet count, high values are not a risk factor regarding rapamycin treatment. | eligibility issue |
| 6 | 3/1/2007 | Eligibility criteria "no renal cell cancer (suspected or known)" was skipped in the checklist. This was an oversight as the patient does not have known or suspected renal cell cancer. The researcher went back to the radiology report to make sure that there was no mention of renal cell cancer and then forgot to go back and check it on the list. | eligibility issue |
| 7 | 3/1/2007 | The patient was registered at the QACT and started on rapamycin without having a chest Xray and a EKG. | baseline testing issue |
| 8 | 3/1/2007 | The patient was registered at the QACT and started on rapamycin without having a chest Xray and a EKG. | baseline testing issue |
| 9 | 3/1/2007 | The week 17 labs were done on week 19, the week 24 study procedures were completed on week 26. The visit was late to accommodate timing issues that the patient had. | visit date issue |
| 10 | 3/1/2007 | Week 8 procedures were done on week 9, week 16 procedures were done on week18. The protocol was altered to accommodate timing issues that the patient had. | visit date issue |
| 11 | 3/1/2007 | Week 1 labs were drawn at week 2, week 2 labs were drawn at week 4. The protocol was altered because the team tried to accommodate timing issues that the patient had. | visit date issue |
| 12 | 3/1/2007 | week 8 procedures were done on week 9, week 16 procedures were done on week17 and week 24 procedures were done on week 27. The protocol was altered to accommodate timing issues that the patient had. | visit date issue |
| 13 | 3/1/2007 | Week 1 labs were drawn at week 2. The protocol was altered because the team tried to accommodate timing issues that the patient had. | visit date issue |
| 14 | 3/1/2007 | During exit interview on 2/14/07 after DF/HCC internal audit, it was noted that study drug compliance was not documented for all of the audited subjects. | study drug issue |
| 15 | 4/24/2007 | At week 16 of the study, MRIs are performed to determine disease status (stable disease, progressive disease, partial response, complete response, etc.). If the patient is not a responder or progressive disease, target drug level is increased to 9-15ng/mL. Because the patient did not show up for her scheduled week 16 visit, there was a delay in increasing the patient's dose. | treatment issue |
| 16 | 5/9/2007 | Patients are scheduled to come in the 24^th^ week of their study participation for drug level testing and other tests. The patient had oral surgery (unrelated to study) so was off drug for 21 days. Week 24 visit was scheduled for 3 days before she was to resume drug after surgery recovery. The visit was postponed to make up the 21 days of missed doses per protocol. | visit date issue |
| 17 | 6/19/2007 | The treatment schedule continues until week 52 but this patient will be removed from the study on account of hemorrhaging in her kidney angiomyolipoma (AML); the patient will also have her exit examinations and tests delayed one to two weeks because of the limited availability of MRI and CT scan appointment times. There is also a very large AML in the patient's other kidney. | visit date issue, leaving study early |
| 18 | 6/19/2007 | The patient has been off study drug since 4/23/2007 and remained off study drug until after another possible surgery because of a massive kidney angiomyolipoma on the right and a deviated septum. She had stopped rapamycin on April 23, 2007 because she needed oral surgery and since rapamycin is known to impair wound healing, rapamycin treatment should have been held for 2 weeks. She had not restarted the study medicine so was off rapamycin for ~ 2 months prior to acute onset right side pain on 6/19/2007. An AE form was submitted on 6/22/2007. | visit date issue, leaving study early |
| 19 | 6/21/2007 | The patient's week 32 blood, urine, and serum samples will be late or missing because the patient did not show up for her week 32 appointment. At week 32 visits, urine, serum, and blood is collected from patients. | visit date issue, missing data |
| 20 | 7/13/2007 | The patient was taken off drug at week 50 instead of at week 52 per protocol because the patient developed cellulitis followed by cough and a pulmonary infiltrate on chest CT scan was observed. | treatment issue |
| 21 | 7/13/2007 | The patient's drug dose increase will be delayed because the patient's elevated blood pressure may be influenced by the drug. If a patient's drug level is below target level, a dose increase will follow to adjust the drug level until target level is reached. | treatment issue |
| 22 | 7/16/2007 | The patient was removed from study because the patient has not been in contact with study staff despite numerous attempts at contacting patient. Patient drug levels are monitored with regular blood sample collection and doses are adjusted according to drug levels. Because of communication and compliance issues, this patient cannot be followed by study staff. | visit date issues, subject removed from study |
| 23 | 3/6/2007 | A unique subject identifier was not on each page of the consent form. | consent issue |
| 24 | 3/6/2007 | The consent process was not well documented. | consent issue |
| 25 | 3/6/2007 | Physical exam issue, neurologic exam was not documented. | study conduct issue |
| 26 | 3/6/2007 | Toxicities not reported in case report forms | documentation |
| 27 | 4/2/2007 | A 16 year old male with tuberous sclerosis and his parents have discussed this trial with the site PI and all agree this is a good opportunity for this patient. He will turn 17 in September 2007 so he misses the age requirement by 1.5 years and is otherwise eligible for the study. If he cannot be enrolled, he will be treated with off-label and off-study sirolimus in the near future instead so the opportunity to collect research data will be lost. If we obtain IRB approval for this deviation, both the patient and his parents will sign the consent form. | eligibility issue |
| 28 | 4/17/2007 | An adult with tuberous sclerosis would like to enroll in this study and he meets all eligibility criteria except for mild thrombocytopenia. His platelet count is 115,000. Because the thrombocytopenia is mild and stable, we would like to request IRB approval for enrolling this patient despite this deviation from the eligibility requirements. | eligibility issue |
| 29 | 4/26/2007 | Regarding the adult with tuberous sclerosis and mild thrombocytopenia (platelet count of 115,000): we have now received IRB approval for enrolling him but because getting IRB approval on the thrombocytopenia caused a slight delay between baseline testing and enrollment, we are now several days past the required 2 week window on the baseline renal MRI. We are now requesting IRB approval to enroll this subject with a baseline kidney MRI done more than 14 days prior to enrollment. We anticipate that we will be able to complete the enrollment and start study treatment for this participant within 30-40days of the baseline MRI and other baseline lab tests. | baseline testing timing issues |
| 30 | 6/4/2007 | An adult with tuberous sclerosis and mild leukopenia would like to enroll in this study. She meets all eligibility criteria except for mild leukopenia. Her WBC count is 3.8 (on 6/1/07) with an ANC of >2,500. The low WBC is stable (it was 3.5 in July 2006). Because the WBC count is mild and stable, we would like to request IRB approval for enrolling her despite this deviation from the eligibility requirements. | eligibility issue |
| 31 | 7/30/2007 | If target drug level is not maintained at current dosage, the patient's drug dose should be increased 25-50%. Drug levels should be checked 7-14 days after dose increases. Because of the patient's renal issues and a surgery on periungal fibromas on 6/15/07, there was a delay in the dose increase. | treatment issue |
| 32 | 6/19/2007 | An adult with tuberous sclerosis who would like to enroll in this study but some of her eligibility testing was done 33 days prior to study entry. Some of her baseline tests (chest CT, chest x-ray and blood tests) were done on June 13, 2007 and she is scheduled to start treatment on July 16, 2007 because of clinic scheduling issues. We are requesting IRB approval for enrolling her with some baseline tests done 33 days (instead of 30 days) prior to study entry. | baseline testing timing issues |
| 33 | 8/1/2007 | After a loading dose on the day of enrollment, the patient should have been on 2 mg PO QD with dose adjustments according to protocol (section 5.6.2). The patient did not start study drug until the day of her first week visit when her registration has her starting on 6/13/2007. Subsequently, her start date has been changed to 6/21/07. Please note that her kidney MRI is now 21 days prior to enrollment instead of 14 days. | treatment issue |
| 34 | 7/3/2007 | An adult with tuberous sclerosis who would like to enroll in this study at Loma Linda University (LLU) but she has mild leukopenia. She meets all eligibility criteria except for mild leukopenia. Her WBC count was 4.49 on 6/15/07 with an ANC of 2,600 (on 6/15/07) and 3.93 with an ANC of 2,000 (6/25/07). The lower limit of normal at the LLU lab is 4.8 (please note the DFCI lower limit of normal for WBC count is 3.8) . Because the low WBC count is mild and stable, we would like to request IRB approval for enrolling this subject despite this minor deviation from the eligibility requirements. If the IRB decision on this is obtained after July 15, 2007 the following applies: because obtaining IRB approval for the leukopenia could delay her enrollment by 1-2 weeks, we would also like IRB approval to enroll her if her baseline kidney MRI is more than 14 days prior to enrollment. We anticipate that we will be able to complete the enrollment and start study treatment for this participant within 30 days of the baseline kidney MRI and within 60 days of other baseline lab tests (chest CT, chest x-ray and some blood tests). | eligibility issue and baseline testing timing issue |
| 35 | 7/17/2007 | We are submitting this deviation form to let the IRB committee know that a subject was enrolled with some evidence on her MRI on 6-1-07 of a small hemorrhage within the left kidney. This has been present since Dec. 2006 and has been asymptomatic. | eligibility issue |
| 36 | 8/1/2007 | The patient was unable to come into the study site for a follow-up visit after a dose increase within 7-14 days after dose increase per protocol. The patient was suffering from gastroenteritis, keeping her at home, so that there was a delay in the week 1 visit. | visit date issue |
| 37 | 8/7/2007 | An adult with tuberous sclerosis would like to enroll in this study but she has mild leukopenia. She meets all eligibility criteria except for mild leukopenia. On 8/2/07 her WBC was 3.5 ( with a normal ANC of 2,527). We would like to request IRB approval for enrolling GM despite this deviation from the eligibility requirements. Additionally, a protocol amendment to change the normal WBC requirement to ANC > 1500 has just been submitted (on 8/6/07) for IRB approval . | eligibility issue |
| 38 | 9/14/2007 | There are multiple eligibility issues regarding an adult with tuberous sclerosis who would like to enroll in this study. Her history and medical records were reviewed in August 2007 and at that time she met all eligibility criteria except for mild leukopenia so we previously asked for IRB approval to enroll her with this. On 8/2/07 her WBC was 3.5 (low) and ANC 2,527 (normal). She came for her enrollment visit on 9/13/07. Although her WBC is stable at 3.9, there were some additional minor eligibility deviations identified so we would now like IRB approval for the following: 1. the radiology department was concerned about doing a kidney MRI because of possible metal in previous embolization material so a CT scan was done (we have conditional IRB approval for this on amendment submitted 8/7/07); 2. Her repeat SGPT on 9-13-07 was 2.01 times higher than normal (eligibility requirement is < 2x normal); 3. Her platelet count was 355 (upper limit of normal is 350, this issue is also included in the amendment submitted on 8/7/07). If the IRB decision on these issues is obtained after September 26, 2007 the following applies: because obtaining IRB approval for these issues could delay her enrollment by 1-2 weeks, we would also like IRB approval to enroll her if her baseline kidney MRI is more than 14 days prior to enrollment. We anticipate that we will be able to complete the enrollment and start study treatment for this participant within 30 days of the baseline kidney CT and within 60 days of other baseline lab tests (chest CT, chest x-ray and some blood tests). This is consistent with revised protocol submitted on 8/7/07 with conditional IRB approval. | eligibility issue and baseline testing timing issue |
| 39 | 10/22/2007 | In an effort to recruit the final participants for this trial, clinical sites have identified an additional 6 interested participants and are starting to keep waiting lists. Since our target is to enroll 35 and we have already enrolled 30, this is 1 more than needed. We are submitting this deviation form to request IRB approval to change our target enrollment from 35 to 35-41. We have observed 4 partial responses so far, toxicity has been acceptable, and 6 participants have been removed from study early (because of compliance or medical issues unrelated to study drug). Making this change would allow sites to enroll all individuals recruited in this final stage of enrollment. Based on responses and toxicity so far, we believe the potential benefit justifies the potential risks. Since 6 participants have been removed from study early, this minor change in target enrollment will help us get closer to our original target of having 35 complete the study. | enrollment target issue |
| 40 | 11/7/2007 | We have changed the start date for this patient from 12-26-06 to 2-26-07 because this patient missed most of the first 2 months of treatment because study drug was held for antibiotic treatment and to sort out birth control issues. If patients miss rapamycin doses because of surgery or treatment with macrolide antibiotics (erythromycin, clarithromycin and telithromycin), additional days of rapamycin treatment will be added so total duration of rapamycin treatment is 12 months (365 days). In this case, however, since the patient had just started the study drug before being taken off drug for antibiotic treatment and birth control issues, Drs. Dabora and Franz decided that changing the start date to the date the patient restarted drug would be the optimal way to deal with this case. | visit date issue |
| 41 | 11/7/2007 | If target drug level is not maintained at current dosage, the patient's drug dose should be increased 25-50%. Drug levels should be checked 7-14 days after dose increases. Because of the patient's renal issues (significant proteinuria), lower than normal blood counts on 8/10/2007 and 8/23/2007, mild toxicity including mouth ulcers and anemia, and unavailability until 8/29/2007, there was a delay in the dose increase while waiting for test results and renal consults on safety. She has had a partial response at week 16 with rapamycin levels below 3ng/ml. Because of the toxicity issues and her response at a low dose, we plan to continue her on a low dose of rapamycin with levels less than 3ng/ml (typical levels for her have been 1.8-2.5 ng/ml). | treatment issue |
| 42 | 11/16/2007 | In an effort to recruit the final participants for this trial, clinical sites have identified an additional 4 interested participants and are starting to keep waiting lists. Since our target is to enroll 35 and we have already enrolled 32, this is 1 more than needed. We are submitting this deviation form to request IRB approval to change our target enrollment from 35 to 36. We have observed 6 partial responses so far, toxicity has been acceptable, and 6 participants have been removed from study early (because of compliance or medical issues unrelated to study drug). | enrollment target issue |
| 43 | 1/3/2008 | The patient's recommended drug dose increase was delayed because the patient's elevated blood pressure may be influenced by the drug so the clinical site wanted to consult the patient's renal doctor regarding this. If a patient's drug level is below target level, a dose increase of 25-50% will follow to adjust the drug level until target level is reached. Instead of increasing the patient's dose from 7mg per day to 9mg per day, the site increased the patient's dose to 8mg per day for four days until the renal doctor was consulted at which time, the patient's dose was increased to 9mg per day as originally recommended by Dr. Dabora per protocol | treatment issue |
| 44 | 1/3/2008 | The protocol requires a B-HCG test to be done in female patients of reproductive potential every 8-12 weeks until rapamycin is discontinued. This patient was originally scheduled to have a B-HCG test done at her week 16 visit but because the clinical lab did not collect urine at her visit, the test was not done and was delayed for another ~two weeks when the patient came in for drug level test following her dose increase. | lab test timing issue |
| 45 | 1/8/2008 | At the start of drug treatment, patients are given a loading dose of 6mg of Rapamune (rapamycin). A subject was dispensed SIX 2mg tablets of the medication instead of THREE. Despite this, the patient took the correct loading dose of THREE 2mg tablets totaling 6mg of the medication. This was a drug dispensing error and all involved (lead site staff at BWH, staff at MGH and MGH pharmacy) were notified and drug logs have been corrected. This issue has previously been reported to the DFCI IRB on a minor deviation log. | study drug dispensing issue |
| 46 | 1/12/2009 | The patient completed 52 week of treatment; however, the patient is restarting rapamycin at 15 months of being on the study due to kidney angiomyolipoma regrowth. The protocol states that treatment should last for 52 weeks with follow up off of study drug during the months 12-24. The study team has considered potential benefits and risks of restarting rapamycin and feels that restarting treatment is in this patient's best interest. This change will not affect collection of primary endpoint data and will have only a minor impact on collection of secondary endpoint data. We will submit an amendment on this issue. | study conduct issue |
| 47 | 2/1/2008 | If a patient is stable disease or progressive disease at week 16, the study drug target level gets increased to 9-15ng/mL from the starting target of 3-9ng/mL. If a patient achieves a 30% or greater reduction in target lesion size, the patient is considered a partial responder and would not have target drug levels increased. At her week 16 visit, this patient had a 29.4% decrease in target lesion size, just shy of the 30% decrease needed for patients to be categorized as a partial responder. Due to this acceptable response and concerns over the potential for toxicity, Dr. Cutler preferred to keep this patient at her current dose, which keeps her drug levels below the higher drug target levels. | treatment issue |
| 48 | 2/5/2008 | The patient's week 1 blood sample for rapamycin level testing was not collected and sent until week 7 due to scheduling issues. The protocol requires periodic rapamycin levels including at week 1. | visit date issue |
| 49 | 2/5/2008 | The patient's week 16 blood sample for rapamycin level testing was not collected and sent until week 19 due to scheduling issues. The patient's week 40 blood sample for rapamycin level testing was collected and sent early at week 37 due to scheduling issues. The protocol requires periodic rapamycin levels including at week 16 and week 40. | visit date issue |
| 50 | 2/5/2008 | The patient's week 16 blood sample for rapamycin level testing was not collected and sent until week 20 due to scheduling issues. The patient's week 24 blood sample for rapamycin level testing was not collected and sent until week 28 due to scheduling issues. The patient's week 32 blood sample for rapamycin level testing was not collected and sent until week 35 due to scheduling issues. The patient's week 40 visit was dropped due to scheduling issues. This dropped visit will not have any clinical effects since the patient is responding to treatment. The protocol requires periodic rapamycin levels including at week 16, week 24, week 32, and week 40. | visit date issue |
| 51 | 2/5/2008 | The patient's week 8 blood sample for rapamycin level testing was collected and sent early at week 5 due to scheduling issues. The patient's week 24 blood sample for rapamycin level testing was not collected and sent until week 27 due to scheduling issues. The patient's week 32 visit was dropped due to scheduling issues. This dropped visit will not have any clinical effects since the patient is responding to treatment. The protocol requires periodic rapamycin levels including at week 8, week 24, and week 32, however, this patient's erratic work schedule often times conflicts with study schedule. | visit date issue |
| 52 | 2/5/2008 | The patient's week 32 blood sample for rapamycin level testing was not collected and sent until week 35 due to scheduling issues. The protocol requires periodic rapamycin levels including at week 32. | visit date issue |
| 53 | 3/5/2008 | The protocol states that if a patient's trough drug level is above target levels, then the dose would be lowered. Patient NC is currently at the starting dose of 2mg QD but her trough drug level was slightly above that of the target level on 11/6/07 (week 8) at this dose. Because this patient does not have any toxicities, we did not decrease her dose. She had a partial response at week 16 and as of 2/27/08 (week 24), her trough drug level has since come down to within her target range. | treatment issue |
| 54 | 5/16/2008 | The patient's week 16 blood sample for rapamycin level testing was not collected because the patient was off drug at week 16 due to pneumonia. This is a deviation because the protocol requires periodic rapamycin levels including at week 16. Although the week 16 evaluation is missing, the drug level at week 24 was on target, and there were no significant toxicities at week 24. | lab test missing |
| 55 | 5/28/2008 | The patient's week 24 urine B-HCG was not ordered so this data is missing from her 4/4/08 study visit lab form. This participant is 52 years old and reports no menses for four years so is likely post menopausal. She also reports that she is not sexually active. Because pregnancy is unlikely in this participant, the study team plans to order a pregnancy test at her next study visit. The protocol requires a urine B-HCG in females with reproductive potential at baseline and weeks 8, 16, 24, 32, 40, and 52. | lab test missing |
| 56 | 7/17/2008 | Pulmonary function tests including assessment of DLCO, FEV1, and FVC are done in female study patients at baseline and at week 52. Because this patient did not have lymphangioleiomyomatosis or any pulmonary symptoms, pulmonary function testing was not ordered on this patient so this data is missing. | missing data |
| 57 | 8/26/2008 | The patient's week 32 blood sample for rapamycin level testing was not collected and sent until week 36 due to scheduling issues. The protocol requires periodic rapamycin levels including at week 32. | lab test timing issue |
| 58 | 8/26/2008 | The patient's week 32 blood sample for rapamycin level testing was not collected and sent until week 43 due to scheduling issues. The protocol requires periodic rapamycin levels including at week 32. | lab test timing issue |
| 59 | 8/26/2008 | The patient's week 40 blood sample for rapamycin level testing was not collected and sent until week 44 due to scheduling issues. The protocol requires periodic rapamycin levels including at week 40. | lab test timing issue |
| 60 | 10/30/2008 | The patient's week 32 blood sample for rapamycin level testing was not collected and sent until week 37 because the patient was off drug at week 32 due to anemia. The protocol requires periodic rapamycin levels including at week 32. | lab test timing issue |
| 61 | 11/6/2008 | The patient's week 40 blood sample for rapamycin level testing was not collected and sent until week 48 due to scheduling issues. The protocol requires periodic rapamycin levels including at week 40. | lab test timing issue |
| 62 | 11/6/2008 | The patient's week 52 blood sample for rapamycin level testing was not collected and sent until week 55 due to scheduling issues. The protocol requires periodic rapamycin levels including at week 52. | lab test timing issue |
| 63 | 1/12/2009 | The patient completed 52 week of treatment; however, the patient is restarting rapamycin at 15 months of being on the study due to kidney angiomyolipoma regrowth. The protocol states that treatment should last for 52 weeks with follow up off of study drug during the months 12-24. The study team has considered potential benefits and risks of restarting rapamycin and feels that restarting treatment is in this patient's best interest. This change will not affect collection of primary endpoint data and will have only a minor impact on collection of secondary endpoint data. We plan to submit an amendment on this issue. | study conduct issue |
| 64 | 8/4/2009 | All of these patients completed 52 weeks of treatment per the original version of this protocol. We submitted amendment 20 (submitted to IRB on 2/18/09, approved on 3/25/09, activated on 6/16/09) to allow additional drug treatment between months 12-24 on study because of the risk for tumor regrowth when rapamycin is stopped. This change was made based on published data (Bissler et al., 2008) and our own observations on this study and Wyeth has agreed to provide additional free study drug per this amendment (final agreement approved 12/5/08). This deviation form is being submitted because these patients were re-started on rapamycin after week 52 per amendment 20 prior to final approval and activation of that change. The drug re-start dates occurred between 12/11/2008 and April 2009. This was done because the amendment process was underway and the treating clinical site PI felt it was in the best interests of each participant. An amendment on this issue was approved by the IRB on 3/25/09 (submitted 2/2009 and activated 6/2009). | treatment issue |
