# Supplementary material for: Multicenter Phase 2 Trial of Sirolimus for Tuberous Sclerosis: Kidney Angiomyolipomas and Other Tumors Regress and VEGF- D Levels Decrease
Source: PLoS One. 2011 Sep 6;6(9):e23379. doi: 10.1371/journal.pone.0023379 (PMC3167813; doi:10.1371/journal.pone.0023379)
Supplement: Protocol S1 — Trial Protocol. Original protocol v011805. (DOC) [file pone.0023379.s023.doc]

## SECTION 1 – Protocol Summary

**PRINCIPAL/OVERALL INVESTIGATOR:**

Sandra L. Dabora, M.D., Ph.D. Brigham and Women’s Hospital

**CO-INVESTIGATORS:**

Judy Garber, M.D., M.P.H. Dana Farber Cancer Institute

David Kwiatkowski, M.D., Ph.D. Brigham and Women’s Hospital

**CLINICAL SITE PRINCIPAL INVESTIGATORS:**

John Bissler, M.D. Cincinnati Children’s Medical Center

Peter Crino, M.D., Ph.D. UPENN Medical Center

Francis DiMario, M.D. Connecticut Children’s Medical Center

David Ewalt, M.D. Univ. of Texas Southwestern

David Franz, M.D. Cincinnati Children’s Medical Center

Frank McCormack, M.D. Univ. of Cincinnati Medical Center

Daniel Miles, M.D. New York University Medical Center

Arthur Sagalowsky, M.D. Univ. of Texas Southwestern

Elizabeth Thiele M.D., Ph.D. Massachusetts General Hospital

**STUDY TITLE:** A phase II multi-center study of rapamycin for treating kidney angiomyolipomas in TSC or LAM patients

**FUNDING:** National Institutes of Health (NCI) and the Tuberous Sclerosis Alliance.

**SPECIFIC AIMS**:

**Specific Aim 1:** Determine the efficacy of rapamycin (also known as sirolimus and Rapamune®;Wyeth-Ayerst) for the treatment of kidney angiomyolipomas in patients with tuberous sclerosis (TSC) or lymphangioleiomyomatosis (LAM).

**Specific Aim 2:** Determine the toxicity of using rapamycin in this patient population.

**Specific Aim 3:** Collect data on the long term follow up of kidney angiomyolipomas for 12 months after treatment with rapamycin is discontinued.

**Specific Aim 4:** Observe changes that occur in other TSC lesions (tubers, subependymal giant cell astrocytomas, facial angiofibromas, kidney cysts) and pulmonary disease in LAM patients. Changes in renal and pulmonary function will also be monitored. These observations will provide data for future clinical trials for this patient population

**Specific Aim 5**: Collect blood DNA, serum and urine samples periodically for correlative genotype analysis and biomarker studies.

**BACKGROUND AND SIGNIFICANCE:**

Kidney angiomyolipomas are renal tumors that occur frequently in TSC and LAM patients. These tumors can cause significant morbidity and mortality, and effective systemic treatment is not currently available. There is recent evidence in cell culture experiments, animal models, and pathologic specimens from TSC kidney angiomyolipomas, demonstrating that the TSC genes (TSC1 and TSC2) normally function to inhibit mTOR in a conserved signaling pathway involved in nutrient uptake, cell growth and protein translation. Rapamycin has a similar function and may be able to replace the signaling defect that occurs in TSC and LAM due to mutations in the TSC1 or TSC2 gene [1-6]. There is also data in rat and mouse models for TSC kidney disease that rapamycin and analogs can normalize the abnormal mTOR signaling and decrease severity of kidney tumors. These data suggest that rapamycin may be a useful therapeutic agent for the treatment of TSC lesions such as kidney angiomyolipomas.

**RECRUITMENT:**

Patients will be recruited from six TSC specialty clinics in the U.S. Those who meet eligibility criteria for this study will be informed of the study and entered if they wish to participate. Patients will not be directly compensated however they will not be billed for medical care related to this study that is not considered standard of care. We will advertise for participants through the TS Alliance newsletter only if enrollment is slower than expected.

**RESEARCH DESIGN AND METHODS:**

This study is a single arm phase II multi-center study and a two-stage design will be employed. Initially 13 patients will be enrolled and if there is at least 1 response among the first 13 patients, then an additional 22 patients will be enrolled. Both males and females will be included. Eligibility criteria are: diagnosis of TSC or LAM, ages 18-65, at least one measurable kidney angiomyolipoma larger than 2 cm in diameter, good performance status, informed consent, not pregnant or breast feeding, adequate renal, pulmonary and liver function. We will exclude individuals with unstable seizures, active infections, coronary artery disease, evidence for renal cell cancer and/or impending bleeding from kidney angiomyolipomas. Patients who have undergone vascular ablation within six months for kidney angiomyolipomas will also be excluded. Individuals who meet the eligibility criteria and wish to participate will undergo baseline evaluation including physical exam, routine baseline blood work and a baseline MRI of kidneys. They will be registered as a participant in this study and then started on outpatient treatment with rapamycin. They will be monitored periodically by physical exam and blood work to assess safety of the treatment. They will undergo kidney MRI evaluation at 16 weeks, 32 weeks and 52 weeks to assess response of kidney angiomyolipoma(s) to treatment. The primary endpoint will be the response of kidney angiomyolipomas to treatment according to standard criteria (RECIST criteria, details in section 5.7.2 of protocol, [7]) used for assessing response in solid tumors. Only patients with an overall complete response (CR) or partial response (PR) will be considered responders, those with stable disease will not. The standard of care for individuals with angiomyolipomas between 2-5 cm is observation without intervention with kidney imaging every 1-3 years [8-10]. At some centers angiomyolipomas larger than 5 cm are treated with vascular embolization but this procedure is not widely available. Patients with bleeding kidney angiomyolipomas can be treated with vascular embolization or surgery and these individuals will be excluded from this study.

**RISKS AND DISCOMFORTS:**

Risks of participating in this study include the minimal risks of additional kidney MRI scans required for carefully assessing tumor size. Risks of treatment with rapamycin include elevated cholesterol, anemia and thrombocytopenia as well as increased risk of infection. Participants will also undergo additional blood testing and urine sample collection, which is of minimal risk, but there is some discomfort associated with blood collection. There is a risk of disappointment if the treatment is not effective or marginally effective.

**SECTION 3: BODY OF PROTOCOL**

**1.0 INTRODUCTION**

**1.1 Overview**

Kidney angiomyolipomas are tumors consisting of blood vessels, smooth muscle cells and fat cells. These tumors are common in patients with tuberous sclerosis (TSC) and are a significant cause of morbidity and mortality in adult patients with TSC. Kidney angiomyolipomas occur in approximately 75% of TSC patients over the age of 8 years old [9] and there is currently no effective medical therapy for these tumors. Kidney angiomyolipomas often grow and can cause pain, bleeding and/or renal failure. Although surgical resection or vascular embolization is often recommended when kidney angiomyolipomas are large (>5cm) or symptomatic, these treatments are not optimal because they are invasive and can contribute to further impairment of renal function in patients who may: 1) have other kidney angiomyolipomas; 2) have renal cysts (these occur in 30% of TSC patients); 3) develop angiomyolipomas or renal cysts in the future. The standard of care is to observe kidney angiomyolipomas with imaging every 1-3 years and if there is evidence of rapid growth, suspicion of renal cell cancer, symptoms such as pain or bleeding, or evidence of impending bleed, surgical removal or vascular embolization is recommended [8, 9, 11, 12]. Among clinicians who care for adult patients with TSC, there is general agreement that development of medical approaches to treating kidney angiomyolipomas would be useful because management of adult TSC patients with renal disease is often a difficult problem.

Kidney angiomyolipomas are also common in patients with the related pulmonary disorder, lymphangioleiomyomatosis (LAM), occurring in 50% of LAM patients [13]. LAM is characterized by the proliferation of smooth muscle cells in the lungs. TSC gene mutations have been found in the lungs and kidney angiomyolipomas of LAM patients so the molecular defect in mTOR signaling in LAM is similar to that observed in TSC related tumors (see section 1.3) [2, 14, 15]. Pulmonary LAM is a progressive disorder that leads to end-stage lung disease and the only effective treatment is lung transplantation, so there is a significant need for effective systemic therapy for this disease.

**1.2 Review of morbidity and mortality associated with kidney angiomyolipomas.**

Kidney angiomyolipomas cause significant morbidity and mortality in TSC patients.

Although these are generally slow growing tumors, they can cause bleeding, pain and renal failure. The risk of hemorrhage is a particularly difficult problem because, although this can often be successfully managed using either nephron sparing surgery or vascular embolization, there are also reports of shock, death or need for nephrectomy in the setting of bleeding kidney angiomyolipomas [10, 16-24]. There is evidence that kidney angiomyolipomas and other renal manifestations of TSC are an important cause of mortality in TSC patients. Shepherd et al. (1991) investigated the causes of death in a group of 355 TSC patients seen at least once at the Mayo Clinic. In this group there were 40 deaths attributable to TSC. Of these, kidney pathology (renal failure, bleeding kidney angiomyolipoma, or renal cell carcinoma) was the cause of death in 31% (11/40) of all patients and in 35% of adult patients >19 years old [25]. This study also showed a decreased life expectancy in the TSC population compared to the normal population.

Two recent studies of kidney angiomyolipomas in cohorts of TSC patients showed that that most kidney angiomyolipomas grow and there was no evidence of spontaneous regression. In a prospective study of kidney lesions in 60 children with TSC ages 1-18, kidney angiomyolipomas occurred in 75% of cases. Growth of kidney angiomyolipomas was common and occurred in 47% of cases (median follow-up was 10.5 years). Spontaneous regression was not observed [9]. In a retrospective review of renal imaging (using ultrasound, CT, or MRI) on 59 TSC patients (ages 3 days to 36 yrs, mean age 11.4 years), kidney angiomyolipomas occurred in 80% and renal cysts occurred in 47% of patients. In this study, there were a total of 80 follow-up renal imaging studies. This included 18 studies with no angiomyolipomas, 30 with unchanged kidney angiomyolipomas, and 32 with angiomyolipomas that increased in size and/or number. Of these, there was an increase in both size and number of kidney angiomyolipomas in 28/32, and increase in size only in 1/32, and and increase in number in 3/32. No cases of spontaneous regression were reported [15]. Observations on tumor growth and lack of tumor regression were consistent between these two studies.

There are three recent reviews in the urology and nephrology literature that summarize additional clinical features associated with kidney angiomyolipomas. Nelson et al. (2002) reviewed the surgical literature from 1986-2001 to summarize clinical findings from 336 cases of kidney angiomyolipomas. Of this group, 19% had TSC and 59% were symptomatic. Patients with TSC were younger, more likely to have multiple tumors, and more likely to hemorrhage than patients with sporadic kidney angiomyolipomas. This review also included a summary of 76 patients who underwent embolization for treatment of angiomyolipomas. Of these 76, 17% had recurrent hemorrhage, 14% required repeat embolization, and 16% underwent surgery [16]. Lendvay and Marshall (2003) reviewed clinical manifestations of TSC reported in 75 articles on TSC identified using Medline to search “ tuberous sclerosis”. They report that renal pathology is significant and includes kidney angiomyolipomas, renal cysts, renal cell carcinoma, oncocytoma, perirenal cysts, and polycystic kidney disease. Renal disease is the primary cause of TSC related death in patients older than 30 years and kidney angiomyolipomas are the most common renal manifestation of TSC. Two-thirds of angiomyolipomas grow over time and 60% are asymptomatic. Morbidity of hemorrhage from kidney angiomyolipomas is significant as symptoms of shock were observed in 10-20% of cases in some reports [10]. In a review by two nephrologists with an interest in TSC kidney disease, Bissler and Kingswood (2004) estimate that there are 10 million people world wide with kidney angiomyolipomas and an estimated 1 million of these also have TSC. They note that although the disease can be benign, it can also be associated with life-threatening hemorrhage. Estimates of the risk of hemorrhage are variable with 3%, 8-18%, 25-50% reported in the literature. Bleeding risk is associated with large size (>4-5 cm) and/or aneurysm formation. Renal failure can occur and about 1% of TSC patients develop end-stage renal disease [12, 26, 27]. Regarding management, these authors comment that in the case of pain, hemorrhage or suspicion of malignancy, nephron sparing surgery is a useful treatment. This is preferred over nephrectomy because angiomyolipomas are usually benign and renal function should be maximized. Unfortunately, even nephron sparing surgery is associated with hemorrhage requiring nephrectomy and loss of some adjacent normal kidney. Embolization is also useful in cases of symptoms from a single identifiable angiomyolipoma. A common complication of embolization is post embolization syndrome (PES) resulting in fever and/or pain after the procedure. Although PES can be significant, this complication may be reduced by treating with a course of prednisone [11]. Embolization is less useful for more diffuse disease, treating multiple lesions, or in cases where it is difficult to identify the source of bleeding. Because of the invasive nature of these treatment options, the current recommendation for asymptomatic angiomyolipomas that are <5cm is to observe without treatment.

There are several key reasons why effective therapy for kidney angiomyolipomas would be beneficial: 1) Kidney angiomyolipomas are common in TSC and LAM. Patients often have multiple and/or bilateral tumors; 2) Although many are asymptomatic, many tumors grow, patients are at risk for developing new angiomyolipomas, and there is a risk for bleeding and/or renal impairment; 3) Hemorrhage from kidney angiomyolipomas is problematic because bleeding is unpredictable and can be life threatening; 4) Although there are invasive treatment options such as nephron sparing surgery, nephrectomy and vascular embolization for kidney angiomyolipomas in specific clinical situations, all of these have risks of complications. In addition, because they are local therapies, they only treat target angiomyolipomas; 5) Treatment is not currently recommended for small and asymptomatic lesions because partial nephrectomy and embolization are invasive and involve some risks. Furthermore, because these are local treatments, these treatment interventions will not prevent the development of new angiomyolipomas.

In TSC patients, it is not possible to predict which angiomyolipomas will grow, form aneurysms, hemorrhage, or cause other symptoms. In addition, patients who develop an acute problem away from a tertiary center may not get medical care where nephron sparing partial nephrectomy or embolization is available. LAM patients are eager for systemic therapy because of the progressive nature of this disease. Because TSC mutations have been found in both the lungs and kidney angiomyolipomas from LAM patients, effective treatment of kidney angiomyolipomas will have significant implications for developing treatments for the devastating pulmonary manifestations of LAM. Given these issues, there is significant interest in the development of effective systemic therapy of kidney angiomyolipomas in TSC and LAM patients. If a well-tolerated medical therapy were available that effectively treated this tumor, it is likely that both TSC and LAM patients would consider this a desirable treatment option. It would also be an important step towards effective therapy for other manifestations of TSC and pulmonary manifestations of LAM.

**1.3 Background: preclinical data demonstrate potential efficacy of rapamycin for the treatment of kidney angiomyolipomas**

There has been recent progress in the understanding of the molecular defect that occurs in TSC lesions such as kidney angiomyolipomas. The genes for TSC have been identified [28, 29] and several recent studies have demonstrated a role for tuberin and hamartin (gene products of TSC2 and TSC1 genes respectively) in a highly conserved signal transduction pathway involving PI3 kinase, Akt, mTOR and S6 kinase [30, 31]. This has been demonstrated in a variety of organisms including humans, rats, mice and drosophila. This pathway is involved in the regulation of nutrient uptake, cell growth and protein translation; a schematic illustration of this pathway is illustrated in Figure 1. The particular abnormality seen in hamartin and/or tuberin deficient cells is the hyperphosphorylation of the downstream targets S6 kinase and S6 (ribosomal subunit). This suggests that the hamartin/tuberin complex is an inhibitor of mTOR and this finding has been confirmed in a variety of organisms in several independent labs [1, 3, 5, 6, 32, 33]. Because these studies demonstrate that the abnormality in cells lacking tuberin or hamartin is the lack of inhibition of mTOR, it is possible that restoring the inhibition of mTOR within this pathway could be a useful strategy for treating TSC.

Rapamycin is an FDA approved immunosuppressive agent that is known to inhibit mTOR [34, 35]. Because of this biochemical similarity in the function of rapamycin and the tuberin/hamartin complex, there is great interest in investigating the potential therapeutic benefit of rapamycin (or other mTOR inhibitors) in TSC related tumors. There is strong evidence in preclinical rodent models of TSC that pharmacologic inhibition of mTOR kinase may be a useful anti-tumor approach in TSC. There is one recent report demonstrating that a short course of rapamycin not only leads to normalization of phosphorylated S6, but also stimulates apoptosis within kidney tumors in the Eker rat model of TSC [4]. We have also observed that treatment of a transgenic mouse model for TSC with a rapamycin analog decreases the severity of kidney and liver tumors in these animals and we observed similar results in a nude mouse model for Tsc2null tumors [36]. It is important to note that tumors from rodent models for TSC have similar abnormalities in mTOR signaling (hyperphosphorylated S6 kinase and S6) as kidney angiomyolipomas from TSC patients [37] and lung tissue from LAM patients [2]. Early evidence that this targeted therapeutic approach may be effective in patients with LAM and TSC is demonstrated by a recent case report in which a 27 year old patient with relapsed LAM in a transplanted lung responded to rapamycin that was being used for immunosuppression [38]. In addition, there were two cases of partial responses of kidney angiomyolipomas from two ongoing single institution trials [39, 40].

Because rapamycin is an approved drug, there is significant information on the safety of this drug in humans and there is significant practical experience using this drug in the organ transplant population [34, 41-44]. There is now some limited experience using rapamycin in TSC and/or LAM patients that shows that it is well tolerated in this population [39, 40].

Figure 1. Schematic diagram of the mTOR signaling pathway. **A**, When hamartin (TSC1 gene product) and tuberin (TSC2 gene product) are normal, they form a complex, TSC2 functions as a GAP for Rheb favoring the Rheb-GDP form, and mTOR kinase activity is low. **B**, When TSC1 or TSC2 is defective, the Rheb-GTP form is favored, mTOR kinase activity is high, and abnormal hyperphosphorylated downstream targets can be detected (such as pS6k, pS6, p4E-BP1). **C**, Disregulated mTOR signaling is normalized in the presence of mTOR kinase inhibitors (CCI-779 or rapamycin).

**2.0 OBJECTIVES**

**2.1:** Determine the efficacy of rapamycin for the treatment of kidney angiomyolipomas in patients with tuberous sclerosis (TSC) or lymphangioleiomyomatosis (LAM) in a multicenter phase II trial.

**2.2:** Determine the toxicity of using rapamycin in this patient population.

**2.3:** Collect data on long term follow up of kidney angiomyolipomas for 12 months after treatment on this study is discontinued.

**2.4**: Observe changes that occur to other TSC lesions (tubers, subependymal giant cell astrocytomas, facial angiofibromas, kidney cysts) and pulmonary disease in LAM patients. Changes in renal and pulmonary function will also be monitored. These observations will provide data for future clinical trials for this patient population.

**2.5**: Collect blood DNA, serum and urine samples periodically during this study for genotype analysis and biomarker studies.

**3.0 ELIGIBILITY**

**3.1 Eligibility Criteria**

3.1.1 Age 18-65 years old, not pregnant or breast-feeding

- - 1. Kidney ANGIOMYOLIPOMA 2 cm or greater (on baseline MRI), no evidence

of impending bleed

3.1.3 ECOG Performance status 0 or 1

3.1.4 No evidence of severe LAM (not oxygen dependent, good performance status)

- - 1. Informed consent, including consent for submission of blood, urine and tissue

samples as described in the appendix.

3.1.6 Adequate renal and liver function (Cr < 4.1, SGOT, SGPT, TBili, Alk Phos all<2x normal)

3.1.7 HCT>27%

- - 1. WBC and platelets within normal limits
    2. Diagnosis of TSC or LAM (diagnosis of TSC using revised diagnostic criteria [45], diagnosis of LAM made by chest CT scan and reviewed by a pulmonologist).
    3. Fertility/Reproductive issues: Patients must be neither pregnant nor plan to become pregnant or conceive a child while on study. The effects of rapamycin on the developing fetus at the doses used in this study are unknown. For this reason, women of child-bearing potential and men must agree to use adequate contraception prior to study entry and for the duration of rapamycin treatment. Because of the possibility of drug interactions and the potential affect of female hormones on the growth of kidney angiomyolipomas, oral contraceptives are not recommended in women enrolled on this study so an effective barrier method of contraception must be used.

### 3.2 Exclusion criteria

3.2.1 Unstable seizures (recent changes in pattern or anti-epileptics)

3.2.3 Bleeding or impending bleed from kidney angiomyolipoma(s)

3.2.3 Severe LAM (dependent on supplemental oxygen or limited performance status)

3.2.4 Evidence for accelerating renal dysfunction or acute renal failure

3.2.5 Renal Cell Ca (suspected or known)

3.2.6 Active infection

3.2.7 Patients will be excluded if they have been treated with any investigational

agent in the 30 days prior to study entry

3.2.8 Patients may not be treated with other investigational agents while on study 3.2.9 Prior history of coronary artery disease

- - 1. Vascular embolization for treatment of kidney angiomyolipoma(s) within 6 months

3.2.11 Patients who must take diltiazem, ketoconazole or rifampin chronically will be excluded because of known drug interactions. Both diltiazem and ketoconazole are strong inhibitors of CYP3A4 and are known to increase rapamycin levels. Rifampin is a known CYP3A4 and P-glycoprotein inducer and is known to significantly reduce rapamycin levels.

**4.0 PATIENT ENTRY**

At the time of enrollment, patients will be registered by contacting the DFCI Quality Assurance Center for Clinical Trials (QACT) at 454 Brookline Ave in Boston (phone-617-632-3761, fax-617-632-2295) during business hours Monday-Friday before treatment begins. The QACT will ask for the following information:

-Name, telephone and pager number of investigator or study coordinator registering patient.

-Protocol name and number

-Date treatment begins

-Patient ID name, address, date of birth, Social Security number, and diagnosis

-Patient ID number

-Treating physician and institution

-Confirmation of eligibility

-Copies of Consent Form signature pages (separate consent forms for treatment and specimen collection)

-Verify that treatment and specimen collection Consent Forms are signed

**5.0 TREATMENT PROGRAM**

**5.1 Introduction**

This protocol is a single arm phase II efficacy study with a two-stage design. All patients will receive rapamycin. This study is designed to determine the response rate of kidney angiomyolipomas to rapamycin. We have selected a two-stage design because although the in vitro and animal model data is very promising and is a targeted therapy based on the molecular defect, there is no published data on using this drug in TSC and/or LAM patients. Although there is one case report in a LAM patient of response with no significant toxicity [38], the two stage design allows us to enroll 13 patients initially. If there is evidence for response and no severe toxicity, an additional 22 patients will be enrolled.

**5.2 Prerequisites**

5.2.1 Timing - All pretreatment baseline blood tests will be performed within 30 days of initiating treatment on this protocol. The baseline kidney MRI imaging documenting measurable disease will be performed within 14 days of initiating treatment.

5.2.2 Lab Parameters –

Stable renal function (Cr<4.1), no evidence of acute renal failure

Adequate liver function (SGOT, SGPT, TBili, Alk Phos all<2x normal

Normal WBC and platelet count

HCT>27%

5.2.3 Pretreatment diagnostic Tests-Baseline kidney MRI according to protocol methods documenting at least 1 kidney angiomyolipoma >2cm and measuring all lesions according to protocol methods.

5.2.4 Baseline lab work- (all should be completed within 60 days of initiating treatment)

Fasting cholesterol profile

Triglycerides

Chest X-Ray

Urine Analysis

Pulmonary Function Testing in females

High-resolution chest CT in females

Brain MRI

EKG

EEG on all patients with abnormal brain scans, history of seizures, or son anti- epileptic medication

5.2.5 Pretreatment Research lab work

Blood sample (10 cc) for DNA extraction

Blood sample (10 cc) for serum analysis of biomarkers

Urine sample (10 cc) for DNA extraction

Urine sample (10 cc) for analysis of biomarkers

See protocol section 7.2 for additional details on collection and processing of blood and urine samples for correlative research studies.

5.2.6 History and Physical exam

Document skin exam (including grade of facial angiofibromas)

Detailed neurologic exam

**5.3 Additional Requirements**

5.3.1 Rapamycin levels will be checked for monitoring purposes periodically during the 12-month treatment period. Details on timing of rapamycin level monitoring can be found in protocol sections 5.5 and 7.0. If the rapamycin dose is adjusted or changes are made in medications that are CYP3A4 inhibitors or inducers (see protocol appendix), rapamycin levels will be checked weekly until an appropriate trough level is achieved. All rapamycin levels will be sent to the reference lab at Children’s Hospital (Boston, MA) to ensure consistency of these measurements at the different clinical sites. Details regarding logistics of rapamycin monitoring can be found in section 7.1.

5.3.2 Fasting cholesterol and triglyceride profile (week 1, then every 8-12 weeks until rapamycin discontinued (see section 7.0). Treatment with dietary modification and a lipid lowering agent will be initiated for LDL > 200.

- - 1. CBC and chemistry 12 panel for toxicity monitoring (1 week, 2 weeks,

then every 8-12 weeks until rapamycin discontinued. Chemistry 12 panel includes electrolytes, BUN/creatinine, bilirubin (total and direct), SGOT, SGPT. Urine analysis for toxicity monitoring will be done every 8-12 weeks. Urine B-HCG will be done in females of reproductive potential every 8-12 weeks until rapamycin is discontinued. See section 7.0 for schedule of tests in table format.

5.3.4 Kidney MRI at 16 weeks, 32 weeks and 52 weeks during treatment. For long term follow up, kidney MRIs will also be obtained at 18 months and 24

months.

5.3.5 Skin and Neurologic exam will be documented at 16 weeks, 32 weeks, 52 weeks, 18 months and 24 months.

5.3.6 If clinically indicated, follow up brain MRI will be obtained at 12 months

5.3.7 If baseline PFTs or high resolution chest CT are abnormal at the start of treatment, they will be obtained again at 12 months or if clinically indicated.

- - 1. Research blood work (10 cc) will be obtained at 16 weeks, 32 weeks, and

52 weeks for serum biomarker studies.

5.3.9 Research Urine samples will also be obtained at 16 weeks, 32 weeks, and

52 weeks for biomarker studies.

- 1. **Drug Formulation and Procurement**

5.4.1-Rapamycin (Sirolimus, Rapamune)

Mode of action: Rapamycin is a macrocylic lactone produced by Streptomyces hygroscopicus. It is an immunosuppressive agent. It inhibits T lymphocyte activation and proliferation that occurs in response to antigenic and cytokine (Interleukins 2,4, and 15) stimulation by a mechanism that is distinct from that of the other immunosuppressants. Rapamycin also inhibits the activation of mTOR (mammalian Target of Rapamycin), a key regulatory kinase.

How supplied: Rapamycin is available as a white, triangular-shaped tablet containing 1 mg per tablet, as a yellow/beige triangular-shaped tablet containing 2 mg per tablet, and also as an oral solution containing 1mg/ml.

Preparation and Administration: See section 5.5.1

Storage and Stability: Tablets-protect from light and store at controlled room temperature (20ºC to 25ºC).

Oral Solution-protect from light and refrigerate at 2ºC-8ºC. Once the bottle is opened, the contents should be used within one month.

Route of adminstration: oral

Availability: Rapamycin (sirolimus) is commercially available (Wyeth Pharmaceuticals) and will be stored according to the manufacturers recommendation. The overall PI will seek a free supply of sirolimus for this study

Treatment with rapamycin will be administered on an outpatient basis. Although there is more than minimal risk in this study, there are substantial preclinical data suggesting a potential benefit. Because rapamycin is an approved drug, there is substantial safety data from completed phase I-III trials and the overall experience is that rapamycin is well tolerated by most patients. Expected adverse events and dose modification are described in Section 6.

**5.5 Drug Administration**

This study uses rapamycin, which is commercially available and will be administered according to standard guidelines and instructions found in the package inserts with modifications according to recent studies in the organ transplant population.

Patients will be started on the tablet form. The oral suspension will be available to patients who are unable to tolerate the tablet form. An updated Rapamune package insert is in the protocol appendix.

- - 1. Rapamycin treatment will be initiated with a loading dose of 6 mg PO on the day that the patient is enrolled followed by a dose of 2 mg PO QD. Dosing will be adjusted to maintain a target level of 3-9 ng/ml. Dosing adjustments will be made as described in section 5.6.2. Patients will be maintained at a trough level of 3-9 ng/ml for the first 16 weeks of the study. Rapamycin levels will be checked weekly until a stable target trough level is achieved. Rapamycin levels will also be checked at 8 weeks and 16 weeks. See section 7.0 for schedule of rapamycin level monitoring and protocol section 7.1 for details regarding the logistics of rapamycin level monitoring.
    2. The dose of rapamycin will be increased to a target level of 9-15 ng/ml after 16 weeks unless there is evidence for a PR or CR by kidney MRI. This will be done by increasing the dose by 25-50% increments until a stable trough level of 9-15 ng/ml is achieved. Rapamycin levels will be checked weekly until the higher target trough level is achieved. The rapamycin dose will not be increased in patients with a PR or CR at 16 weeks. In these patients, rapamycin levels will be maintained at the lower target level of 3-9 ng/ml for weeks 16-52 of the study. Rapamycin levels will be checked every 8-12 weeks (at 24 weeks, 32 weeks, 40 weeks, and 52 weeks) in all patients until the 12-month treatment period has been completed (see section 7.0 for schedule of rapamycin monitoring and other monitoring details in table format).
    3. The dose of rapamycin will be replaced if the patient vomits within 15 minutes of taking a dose. Premedication with antiemetics is acceptable if there is recurrent vomiting associated with taking rapamycin dose.
    4. A missed dose of rapamycin can be taken up to 12 hours late. For doses up to 6 hours late, no change is needed in the timing of the dose on the following day. If a dose is 6-12 hours late, the dose on the following day should be delayed for 3-6 hours.

5.5.5 Concomitant Therapy with CYP3A4 inhibitors or inducers

Rapamycin is extensively metabolized by the CYP3A4 isoenzyme, thus drugs which are inhibitors or inducers of this enzyme should be avoided if possible or used with caution. The following drugs are contraindicated: diltiazem, ketoconazole and rifampin. Patients who are on these chronically will be excluded because of known drug interactions. Both diltiazem and ketoconazole are strong inhibitors of CYP3A4 and are known to increase rapamycin levels. Rifampin is a known CYP3A4 and P-glycoprotein inducer and is known to significantly reduce rapamycin levels. Because grapefruit juice is a CYP3A4 inhibitor, it may increase levels. Therefore patients will be instructed to avoid grapefruit and grapefruit juice during rapamycin treatment. The appendix contains a list of additional drugs that are known CYP3A4 inhibitors or inducers. These medicines will be used with caution. Since this population often has epilepsy, we will pay particular attention to potential interactions with anti-epileptic medications. Carbamazepine and phenobarbital are examples of anti-epileptic agents that are known CYP3A4 inducers. Because of known drug interactions, rapamycin will be held in patients who require a course of the following macrolide antibiotics: erythromycin, clarithromycin and telithromycin.

- - 1. Rapamycin is known to impair wound healing. Therefore, rapamycin treatment will be held for 2 weeks in patients who require surgery.
    2. If patients miss rapamycin doses because of surgery or treatment with macrolide antibiotics (erythromycin, clarithromycin and telithromycin), additional days of rapamycin treatment will be added so total duration of rapamycin treatment is 12 months (365 days).

**5.6 Adverse Events**

5.6.1 Anticipated Toxicities and Management

- - - 1. Rapamycin

Phase III clinical trials in organ transplant patients indicate that the primary toxicities are hypertriglyceridemia, hypercholesterolemia, thrombocytopenia, anemia, and leukopenia. Other toxicities include hypokalemia, elevated LDH, arthralgia, epistaxis, edema, and infections.

Similar to other immunosuppresant agents, rapamycin may increase the risk of opportunistic infections and lymphoproliferative disorders but these toxicities have been seen in the transplant population that generally is treated with other immunosuppresant agents together with rapamycin. There have been also several rare cases of pneumonitis reported that could be related to rapamycin treatment. As stated above in section 5.5.3, rapamycin is metabolized by CYP3A4 so other drugs that are CYP3A4 inducers or inhibitors may affect rapamycin blood levels. There is now preliminary data in TSC and LAM patients demonstrating that rapamycin is well tolerated. The most common toxicities observed so far in TSC and LAM patients are mouth ulcers (in 7/27 patients) and hypercholesterolemia (in 4/27 patients) [39].

5.6.2 Dose Modifications

5.6.2.1 Rapamycin

The dose of rapamycin will be held and reduced if the trough level exceeds 15 ng/ml at any dose level. For patients with rapamycin trough levels higher than the target range, the dose will be reduced according to trough level as follows:

9-15 ng/ml Reduce dose by 25-50%

16-24 ng/ml Hold dose for 24 hours then resume at 50% of prior dose

25-33 ng/ml Hold dose for 48 hours then resume at 25% of prior dose

over 33 ng/ml Hold dose for 72 hours then resume at 10% of prior dose

If trough levels are low at the 2 mg PO QD dose, the dose will be increased by 25-50% increments until the target dose level is achieved.

**5.7 Therapy Duration**

**5.7.1 Objective responses**- Patients will be evaluated for response to therapy at 16 weeks, 32 weeks and 52 weeks. Response criteria will be determined based on the standardized criteria used to assess response of solid tumors treatment (RECIST criteria; see protocol appendix or http:/cancer.gov/bip/RECIST.htm) [7] and applied to this protocol as summarized below. Only those patients with an overall complete or partial response will be considered a responder.

**Measurable disease** - the presence of at least one measurable lesion.

**Measurable lesions** - lesions that can be accurately measured in at least one dimension with longest diameter >20 mm using conventional techniques.

**Non-measurable lesions** - all other lesions, including small lesions (longest diameter <20 mm).

* All measurements should be taken and recorded in metric notation, using a ruler or calipers. All baseline evaluations should be performed as closely as possible to the beginning of treatment and never more than 4 weeks before the beginning of the treatment.

* The same method of assessment and the same technique should be used to characterize each identified and reported lesion at baseline and during follow-up.

**Baseline documentation of “Target” and “Non-Target” lesions**

* All measurable lesions up to a maximum of five lesions per kidney and 10 lesions in total, should be identified as ***target lesions*** and recorded and measured at baseline.

* Target lesions should be selected on the basis of their size (lesions with the longest diameter) and their suitability for accurate repeated measurements (either by imaging techniques or clinically).

* A sum of the longest diameter (LD) for *all target lesions* will be calculated and reported as the baseline sum LD. The baseline sum LD will be used as reference by which to characterize the objective tumor.

* All other lesions should be identified as ***non-target lesions*** and should also be recorded at baseline. Measurements of these lesions are not required, but the presence or absence of each should be noted throughout follow-up.

**5.7.2 Response Criteria**

**Evaluation of target lesions**

* Complete Response (CR): Disappearance of all target lesions

* Partial Response (PR): At least a 30% decrease in the sum of the LD of target lesions, taking as reference the baseline sum LD

* Progressive Disease (PD): At least a 20% increase in the sum of the LD of target lesions, taking as reference the smallest sum LD recorded since the treatment started or the appearance of one or more new lesions

* Stable Disease (SD): Neither sufficient shrinkage to qualify for PR nor sufficient increase to qualify for PD, taking as reference the smallest sum LD since the treatment started

**Evaluation of non-target lesions**

* Complete Response (CR): Disappearance of all non-target lesions

* Incomplete Response/ Stable Disease (SD): Persistence of one or more non-target lesion(s) and/or maintenance of tumor marker level above the normal limits

* Progressive Disease (PD): Appearance of one or more new lesions and/or unequivocal progression of existing non-target lesions (1)

**Overall response (from Therasse et al., 2000)**

| Target lesions | Nontarget lesions | New lesions | Overall Response |
| --- | --- | --- | --- |
| CR | CR | No | CR |
| CR | Incomplete response/SD | No | PR |
| PR | Non-PD | No | PR |
| SD | Non-PD | No | SD |
| PD | Any | Yes or No | PD |
| Any | PD | Yes or No | PD |
| Any | Any | Yes | PD |

**5.7.3 Therapy Duration**- the anticipated duration of therapy is 12 months. Treatment will be stopped early only if unacceptable toxicity develops or if the patient requests to withdraw from the study.

**6.0 ADVERSE EVENT REPORTING**

According to FDA and institutional criteria, serious adverse events will be reported for all side effects that are both serious (life-threatening or fatal) and unexpected. These events will be reported by phone within 24 hours to the overall PI and the members of the Data and Safety Monitoring Committee. The overall PI will immediately notify all co-investigators and clinical site principal investigators of such events. A written report of such events will follow within 10 working days. All such events will be reported to the DFCI Human Protection Committee by the overall PI. The conduct of the study will comply with all FDA safety reporting requirements and the safety reporting requirements of all participating institutions.

1. **REQUIRED DATA**

|  | On study | wk1 | wk 2 | wk 8 | wk 16 | wk 17 | wk 24 | wk 32 | wk 40 | wk 52 | 18 mos | 24 mos |
| --- | --- | --- | --- | --- | --- | --- | --- | --- | --- | --- | --- | --- |
|  |  |  |  |  |  |  |  |  |  |  |  |  |
| Informed consent (pt or guardian) | X |  |  |  |  |  |  |  |  |  |  |  |
| History and physical | X |  |  | X | X |  | X | X | X | X | X | X |
| documentation of skin exam ^a^ | X |  |  |  | X |  |  | X |  | X | X | X |
| Renal MRI (size and # angiomyolipomas  &cysts) | X |  |  |  | X |  |  | X |  | X | X | X |
| rapamycin level (trough)^b^ |  | X | X ^c^ | X | X | X ^d^ | X | X | X | X |  |  |
| Chem 12 | X | X | X ^e^ | X | X | X ^f^ | X | X | X | X |  |  |
| CBC | X | X | X ^e^ | X | X | X ^f^ | X | X | X | X |  |  |
| Fasting cholesterol profile | X | X |  | X | X | X | X | X | X | X |  |  |
| Triglycerides | X | X |  | X | X | X | X | X | X | X |  |  |
| Baseline EEG ^g^ | X ^g^ |  |  |  |  |  |  |  |  |  |  |  |
| CXR | X |  |  |  |  |  |  |  |  |  |  |  |
| Urine Analysis | X |  |  | X | X |  | X | X | X | X | X | X |
| Urine B-HCG ^h^ | X |  |  | X | X |  | X | X | X | X |  |  |
| high res. Chest CT (females only) | X |  |  |  |  |  |  |  |  | X |  |  |
| recent brain MRI (within 3 months) | X |  |  |  |  |  |  |  |  | X |  |  |
| PFTs (females only) | X |  |  |  |  |  |  |  |  | X |  |  |
| EKG | X |  |  |  |  |  |  |  |  |  |  |  |
| Research Blood for DNA sample ^i^ | X |  |  |  |  |  |  |  |  |  |  |  |
| Research Blood for serum-bank ^j^ | X |  |  |  | X |  |  | X |  | X |  | X |
| Research Urine, bank | X |  |  |  | X |  |  | X |  | X |  | X |
| Review eligibility checklist | X |  |  |  |  |  |  |  |  |  |  |  |

^a^ Including photograph of face for patients with facial angiofibromas

^b^ If there are changes in medications metabolized by CYP3A4, rapamycin levels will be checked weekly until appropriate stable trough level is achieved

^c^ Rapamycin levels will be checked weekly as dose is adjusted until stable trough level of 3-9 ng/ml is achieved (see additional details in section 7.1)

^d^ For patients with SD or PD, rapamycin levels will be checked weekly as dose is adjusted until stable trough level of 9-15 ng/ml is achieved

^e^ Chem 12 and CBC weekly until rapamycin level of 3-9 ng/ml achieved

^f^ Chem 12 and CBC weekly until rapamycin level of 9-15 ng/ml achieved (for patients with SD or PD at 16 weeks)

^g^ EEG in patients with a history of seizures or abnormal brain MRI at baseline and during rapamycin treatment as clinically indicated

^h^ Urine B-HCG only in females with reproductive potential

^i^ For TSC1 and TSC2 mutation analysis and genotype studies (additional details in section 7.2 and appendix)

^j^ For biomarker studies (additional details in section 7.2 and appendix)

- 1. **Rapamcyin level monitoring**

Because rapamycin levels can vary from lab to lab, we will use a single lab for measuring

all rapamycin levels. Whole blood will be collected in 2 ml EDTA tubes (purple cap). For sample tracking purposes, all blood samples for rapamycin levels will be shipped by express mail to

Dr. Dabora’s lab:

Attn: Sandra Dabora, Paul Sudentas or Laifong Lee

Division of Hematology, Brigham and Women’s Hospital

One Blackfan Circle, CHRB 6^th^ Floor

Boston, MA 02115

Phone: 617-355-9033 or 617-355-9004

Upon arrival, samples will be hand delivered by Sandra Dabora, Paul Sudentas or Laifong Lee to the lab of Dr. Nader Rifai at the following address at Children’s Hospital where rapamycin levels will be measured.

Nader Rifai Ph.D., Lab Director

Lab Medicine, FA 755

Children's Hospital Boston

300 Longwood Ave,

Boston MA 02115

Phone: 617-355-6733

All samples arriving at Children’s Hospital prior to 11 AM will be run the same day and those arriving after 11AM will be run the next day. Including time for shipping, the anticipated turn around time for rapamycin levels will be within 2 days for samples drawn Monday-Wednesday and within 4 days for samples drawn on Thursdays-Fridays. Results will be reported on the day they are available to the clinical site PI by e-mail and fax.

**7.2 COLLECTION OF BLOOD, URINE AND TISSUE SAMPLES FOR RESEARCH STUDIES**

- - 1. **Genetic and biomarker analyses**

Several blood and urine samples will be collected for correlative genetic and biomarker

studies. Tissue samples will be collected only if patients require surgery for a TSC or LAM related lesion. Potential genetic studies on blood, urine and tissue samples may include

TSC mutation analysis, interferon-gamma (IFN-g) genotyping, genotyping polymorphisms in candidate modifier genes, genotyping polymorphisms in genes relevant to drug metabolism. Biomarker studies may include the analysis of vascular endothelial growth factor (VEGF) levels and other possible secreted biomarkers. There are additional details regarding statistical issues for correlative studies in section 9.3.

**7.2.2 Blood sample collection**

Blood samples for research will be collected at the time of enrollment on. At the time of enrollment, two 10 ml tubes of blood will be drawn into EDTA containing tubes. An additional 10 ml tube of blood will be collected (also in EDTA containing tubes) four additional times (at weeks 16, 32, 52 and at 24 months). Samples will be shipped by overnight mail to:

Dr. Sandra Dabora

Hematology Division

Brigham and Women’s Hospital

One Blackfan Circle, CHNRB 6^th^ Floor

Boston, MA 02115

Upon arrival in Dr. Dabora’s lab, plasma will be separated from the cellular components by centrifugation. The plasma will be allocated into plastic freezing tubes, catalogued, and frozen at –70 °C for biomarker analyses. DNA will be extracted from the second tube for genotype analysis relevant to TSC and LAM, including modifier genes and genes relevant to drug metabolism.

**7.2.3 Urine sample collection**

Patients will be also be asked to give 5 urine specimens for research studies (at enrollment, week 16, week 32, week 52, and at 24 months). Samples will be shipped by overnight mail to Dr. Dabora’s Lab (address above).

Upon arrival in Dr. Dabora’s lab, urine will be allocated into plastic freezing tubes, catalogued, and frozen at –70 °C for VEGF analyses and future studies.

**7.2.4 Tissue sample collection**

If surgical resection of any lesions relevant to TSC and/or LAM occurs while patient is enrolled, frozen and/or fixed tissue will be obtained for biomarker analyses. If the lesion is sufficiently large, both frozen and fixed tissue will be obtained.

Additional details regarding collection of specimens can be found in the appendix.

**8.0 MODALITY REVIEW**

Response to therapy will be measured by kidney MRI imaging. To ensure consistency in tumor measurements from scan to scan in individual patients and across the six participating clinical sites, for analysis of the final results, all kidney MRIs will be reviewed by the Dana-Farber radiology department.

1. **STATISTICAL PLAN**

**9.1 Primary Objectives**

The primary endpoint is the proportion of patients exhibiting objective response according to RECIST criteria (see appendix or http:/cancer.gov/bip/RECIST.htm) [7]. For each arm, a true but unobserved underlying response rate of 20% would be considered worthy of further study, while a response rate of 5% would be considered not promising.

A two-stage design will be employed. First, 13 eligible patients will be randomized. If there are no responses among the 13 eligible patients, the study will be discontinued and the regimen declared unpromising. If there is at least 1 response among the 13 eligible patients, then an additional 22 eligible patients will be enrolled. If 4 or more responses are observed among 35 total eligible patients on a given arm, the regimen will be considered worthy of further study. Alternatively, if 3 or fewer responses are observed, the regimen will be declared unpromising. A study with this design has 51% probability of stopping early if the true response rate is 5%, 8.4% probability of declaring the treatment effective at the end of the study if the response rate is 5%, and 90% probability of declaring the treatment effective if the response rate is 20%. If 4 responses are observed, the 90% exact two-stage binomial confidence interval on the response rate will be (4.2, 25.6%).

Toxicity is another important endpoint of the study. NCI Common Toxicity Criteria will be used to assess toxicity (see appendix or http://ctep.cancer.gov/reporting/ctc.html). With 35 treated patients, the maximum width of the 90% confidence interval on the rate of severe toxicities will be no wider than 30%. The probability of observing at least 1 severe toxicity with a true rate of 5% is approximately 83%. If the true rate of severe toxicity is 1%, the probability of observing at least 1 patient with severe (grade 3 or 4 toxicity) is 29.7% and the probability of observing 2 or more patients with severe toxicity is 4.8%.

**9.2 Secondary objectives - Response of other disease manifestations to rapamycin**

TSC is a multisystem disorder so there is great interest in determining whether manifestations of these diseases other than kidney angiomyolipomas respond to rapamycin. LAM is a progressive pulmonary disorder so we are interested in evaluating pulmonary endpoints in these patients.

**9.2.1 Neurologic**

To assess neurologic manifestations of TSC, a brain MRI and detailed neurologic exam will be obtained at baseline and again at 52 weeks. The neurologic exam will include a functional assessment of cognitive status, such as the Mini Mental Status. Qualitative changes of brain lesions by MRI will be assessed by site PI's. The proportion of patients with change in cognitive status and the proportion with changes in brain lesions will be reported, along with a 95% confidence interval.

**9.2.2 Skin**

A detailed skin examination will be performed on all patients at baseline and again at 52 weeks. Using the TSC Clinical Features Grading Form (see appendix), investigators will assess the status of hypomelanotic macules, shagreen patch, forehead plaque, and facial angiofibromas. Investigators will also photograph facial angiomyolipomas and other skin lesions at baseline and again at 16 weeks, 32 weeks, and 52 weeks. Facial angiofibromas will be graded according to Dabora et al (2001) [46]:

Grade 0: None

Grade 1: macular (flat) lesions

Grade 2: papular lesions < 3 mm diameter

Grade 3: papular lesions > 3 mm diameter and/or extending below mouth

The grade at baseline and at each time point will be tabulated. Changes from baseline to best grade post-baseline and from baseline to 52 weeks will be evaluated for each patient. The hypothesis of no change will be examined using the Wilcoxon signed rank test.

**9.2.3 Renal cysts and renal function**

Renal cysts will be evaluated qualitatively by kidney MRI at baseline, 16 weeks, 32 weeks, and 52 weeks. The proportion of patients with improvement by week 52 will be reported, along with a 95% confidence interval. Levels of serum creatinine and BUN will be assessed over time at the same intervals. Based on the observed distribution of values, either a linear or non-linear model will be fit for each patient. If a linear model is adequate to provide good fit, the null hypothesis that there is no change over time will be tested by evaluating slopes.

**9.2.4 Pulmonary**

Because pulmonary disease associated with TSC and LAM affects only women, spiral CT and pulmonary function tests will be used in women only to assess the impact of rapamycin therapy. Differences from baseline in FEV1, FVC, and DLCO will be examined using the Wilcoxon signed rank test. The proportion of patients with qualitative change in the number or size of pulmonary lesions by spiral CT will be reported, along with a 95% confidence interval.

**9.3 Secondary objectives - genotype and biomarker analyses**

VEGF levels will be assessed using ELISA at 16 weeks, 32 weeks, and 52 weeks. The feasibility of capturing and analyzing this information is the primary endpoint of interest. We will consider this correlative analysis to be feasible if VEGF levels can be successfully characterized in at least half of the patients at each time point. The proportion with assessable VEGF levels will be described along with 95% confidence intervals.

Differences in the pattern of change between responders and non-responders will also be examined using mixed effects model with a time-by-response interaction term. Statistical power will be limited for this analysis and will depend on the proportion of patients classified as responders. As an illustration of statistical power that will exist, we consider a simpler model that uses only baseline and 52-week values. For each patient, a change score will be calculated. The difference in change scores between responders and non-responders will be examined using a t-test. Use of this test assumes that change scores are normally distributed. In a study by Heymach et al., (2004), change in urine VEGF from baseline was measured after 2 cycles of chemotherapy among 12 sarcoma patients [47]. The mean change was 41.6 pg/ml and the standard deviation of change was 131.0 in this small sample. The within-patient correlation between measurements was 0.56. Assuming baseline levels of 70 pg/ml and standard deviation of change= 50 pg/ml in both groups, 51% response rate (18 responders, 17 non-responders) and an intra-patient correlation of 0.5, the study will have reasonable power (80%) to detect a difference in change between 60 pg/ml in responders and 10 pg/ml in non-responders.

**9.4 Accrual**

We anticipate that after IRB approval is obtained at all 6 sites and contracts for rapamycin distribution are in place for all 6 sites, the expected accrual rate to the study will be 1 - 4 patients per month. Given this rate, we expect the first 13 patients to be enrolled within 3-12 months. If there is at least one response, we anticipate the accrual of the additional 22 patients will occur within months 12-30. We anticipate that by month 42, all enrolled patients will have completed the 12-month course of treatment and the primary endpoint data will be collected. We expect accrual of all 35 patients to the study to be completed in approximately 2.5 years (30 months).

**10.0 REFERENCES**

1. Gao, X., Y. Zhang, P. Arrazola, O. Hino, T. Kobayashi, R.S. Yeung, B. Ru, and D. Pan. Tsc tumour suppressor proteins antagonize amino-acid#150;TOR signalling*.* Nat Cell Biol, 2002. 4(9): p. 699-704.

2. Goncharova, E.A., D.A. Goncharov, A. Eszterhas, D.S. Hunter, M.K. Glassberg, R.S. Yeung, C.L. Walker, D. Noonan, D.J. Kwiatkowski, M.M. Chou, R.A. Panettieri, Jr., and V.P. Krymskaya. Tuberin regulates p70 S6 kinase activation and ribosomal protein S6 phosphorylation. A role for the TSC2 tumor suppressor gene in pulmonary lymphangioleiomyomatosis (LAM)*.* J Biol Chem, 2002. 277(34): p. 30958-67.

3. Inoki, K., Y. Li, T. Zhu, J. Wu, and K.L. Guan. TSC2 is phosphorylated and inhibited by Akt and suppresses mTOR signalling*.* Nat Cell Biol, 2002. 4(9): p. 648-57.

4. Kenerson, H.L., L.D. Aicher, L.D. True, and R.S. Yeung. Activated Mammalian target of rapamycin pathway in the pathogenesis of tuberous sclerosis complex renal tumors*.* Cancer Res, 2002. 62(20): p. 5645-50.

5. Kwiatkowski, D.J., H. Zhang, J.L. Bandura, K.M. Heiberger, M. Glogauer, N. el-Hashemite, and H. Onda. A mouse model of TSC1 reveals sex-dependent lethality from liver hemangiomas, and up-regulation of p70S6 kinase activity in Tsc1 null cells*.* Hum Mol Genet, 2002. 11(5): p. 525-34.

6. Tee, A.R., D.C. Fingar, B.D. Manning, D.J. Kwiatkowski, L.C. Cantley, and J. Blenis. Tuberous Sclerosis Complex-1 and 2 gene products function together to inhibit mammalian target of Rapamycin- (mTOR) mediated downstream signalling. Proc Natl Acad Sci U S A, 2002. 99: p. 13571-6.

7. Therasse, P. New guidelines to evaluate the response to treatment in solid tumors. European Organization for Research and Treatment of Cancer, National Cancer Institute of the United States, National Cancer Institute of Canada. 2000. 92: p. 205-216.

8. Roach, E.S., F.J. DiMario, R.S. Kandt, and H. Northrup. Tuberous sclerosis consensus conference: recommendations for diagnostic evaluation*.* J Child Neurol, 1999. 14(6): p. 401-7.

9. Ewalt, D.H., E. Sheffield, S.P. Sparagana, M.R. Delgado, and E.S. Roach. Renal lesion growth in children with tuberous sclerosis complex*.* J Urol, 1998. 160(1): p. 141-5.

10. Lendvay, T.S. and F.F. Marshall. The TS Complex and its highly variable manifestations*.* J. Urol., 2003. 169: p. 1635-1642.

11. Bissler, J.J., J. Racadio, L.F. Donnelly, and N.D. Johnson. Reduction of postembolization syndrome after ablation of renal angiomyolipoma*.* American Journal of Kidney Diseases [Online], 2002. 39(5): p. 966-71.

12. Bissler, J.J. and J.C. Kingswood. Renal angiomyolipomata*.* Kidney Int, 2004. 66(3): p. 924-34.

13. Sullivan, E.J. Lymphangioleiomyomatosis: a review*.* Chest, 1998. 114(6): p. 1689-703.

14. Astrinidis, A., L. Khare, T. Carsillo, T. Smolarek, K.S. Au, H. Northrup, and E.P. Henske. Mutational analysis of the tuberous sclerosis gene TSC2 in patients with pulmonary lymphangioleiomyomatosis*.* J Med Genet, 2000. 37(1): p. 55-7.

15. Casper, K.A., L.F. Donnelly, B. Chen, and J.J. Bissler. Tuberous sclerosis complex: renal imaging findings*.* Radiology, 2002. 225(2): p. 451-6.

16. Nelson, C.P. and M.G. Sanda. Contemporary diagnosis and management of renal angiomyolipoma*.* J Urol, 2002. 168(4 Pt 1): p. 1315-25.

17. Mouded, I.M., B.M. Tolia, J.E. Bernie, and H.R. Newman. Symptomatic renal angiomyolipoma: report of 8 cases, 2 with spontaneous rupture*.* J Urol, 1978. 119(5): p. 684-8.

18. Takigawa, H., M. Yano, and S. Kagawa. [Management of hemorrhage secondary to renal angiomyolipoma]*.* Hinyokika Kiyo, 1985. 31(10): p. 1767-71.

19. Martinez-Pecino, M., F. Camacho Ortuno, J.L. Madrid Rondon, E. Sanchez Rodriguez, M. Rodriguez Ramos, S. Vazquez Navarrette, J.M. Rodriguez Santamaria, and E. Garcia Romero. [Intraperitoneal hemorrhage after labor as manifestation of renal angiomyolipoma]*.* Arch Esp Urol, 1989. 42(1): p. 25-8.

20. Rapp, N., G. Audibert, P.F. Gerbaud, G. Grosdidier, and M.C. Laxenaire. [Spontaneous retroperitoneal hematoma: a rare cause of hemorrhagic shock]*.* Ann Fr Anesth Reanim, 1994. 13(6): p. 853-6.

21. Lee, J.D., H.C. Chang, S.H. Chu, S. Hsueh, and Y.K. Soong. Massive retroperitoneal hemorrhage from spontaneous rupture of a renal angiomyolipoma during pregnancy. A case report*.* J Reprod Med, 1994. 39(6): p. 477-80.

22. Daskalopoulos, G., I. Karyotis, I. Heretis, P. Anezinis, E. Mavromanolakis, and D. Delakas. Spontaneous perirenal hemorrhage: a 10-year experience at our institution*.* Int Urol Nephrol, 2004. 36(1): p. 15-9.

23. Shiba, M. and H. Takatera. [Retroperitoneal angiomyolipoma with rapidly progressing intracystic hemorrhage and lymph node involvement: a case report]*.* Hinyokika Kiyo, 2003. 49(10): p. 611-3.

24. Benjelloun, M., R. Rabii, M.H. Mezzour, A. Joual, S. Bennani, and M. el Mrini. [Hemorrhagic bilateral renal angiomyolipoma]*.* Prog Urol, 2003. 13(4): p. 683-5.

25. Shepherd, C.W., M.R. Gomez, J.T. Lie, and C.S. Crowson. Causes of death in patients with tuberous sclerosis*.* Mayo Clin Proc, 1991. 66(8): p. 792-6.

26. Clarke, A., E. Hancock, C. Kingswood, and J.P. Osborne. End-stage renal failure in adults with the tuberous sclerosis complex*.* Nephrol Dial Transplant, 1999. 14(4): p. 988-91.

27. Schillinger, F. and R. Montagnac. Chronic renal failure and its treatment in tuberous sclerosis*.* Nephrol Dial Transplant, 1996. 11(3): p. 481-5.

28. Consortium, E.C.T.S. Identification and characterization of the tuberous sclerosis gene on chromosome 16. Cell, 1993. 75(7): p. 1305-15.

29. van Slegtenhorst, M., R. de Hoogt, C. Hermans, M. Nellist, B. Janssen, S. Verhoef, D. Lindhout, A. van den Ouweland, D. Halley, J. Young, M. Burley, S. Jeremiah, K. Woodward, J. Nahmias, M. Fox, R. Ekong, J. Osborne, J. Wolfe, S. Povey, R.G. Snell, J.P. Cheadle, A.C. Jones, M. Tachataki, D. Ravine, and D.J. Kwiatkowski. Identification of the tuberous sclerosis gene TSC1 on chromosome 9q34*.* Science, 1997. 277(5327): p. 805-8.

30. Gao, X. and D. Pan. TSC1 and TSC2 tumor suppressors antagonize insulin signaling in cell growth*.* Genes Dev, 2001. 15(11): p. 1383-92.

31. Potter, C.J., H. Huang, and T. Xu. Drosophila Tsc1 functions with Tsc2 to antagonize insulin signaling in regulating cell growth, cell proliferation, and organ size*.* Cell, 2001. 105(3): p. 357-68.

32. Manning, B.D., A.R. Tee, M.N. Logsdon, J. Blenis, and L.C. Cantley. Identification of the tuberous sclerosis complex-2 tumor suppressor gene product tuberin as a target of the phosphoinositide 3-kinase/akt pathway*.* Mol Cell, 2002. 10(1): p. 151-62.

33. Potter, C.J., L.G. Pedraza, and T. Xu. Akt regulates growth by directly phosphorylating Tsc2*.* Nat Cell Biol, 2002. 4(9): p. 658-65.

34. Garber, K. Rapamycin's resurrection: a new way to target the cancer cell cycle*.* J Natl Cancer Inst, 2001. 93(20): p. 1517-9.

35. Hidalgo, M. and E.K. Rowinsky. The rapamycin-sensitive signal transduction pathway as a target for cancer therapy*.* Oncogene, 2000. 19(56): p. 6680-6.

36. Lee, L., P. Sudentas, B. Donohue, K. Asrican, A. Worku, V. Walker, Y. Sun, K. Schmidt, M.S. Albert, N. El Hashemite, A.S. Lader, H. Onda, H. Zhang, D.J. Kwiatkowski, and S.L. Dabora. Efficacy of a rapamycin anaolog (CCI-779) and IFN-g in tuberous sclerosis mouse models*.* submitted: p. submitted.

37. El-Hashemite, N., H. Zhang, E.P. Henske, and D.J. Kwiatkowski. Mutation in TSC2 and activation of mammalian target of rapamycin signalling pathway in renal angiomyolipoma*.* Lancet, 2003. 361(9366): p. 1348-9.

38. Niedermeyer, J. Rapamycin for recurrent LAM following lung transplantation-a first experience*.* Abstract at LAM foundation research conference, April 2003, 2003.

39. Bissler, J., D. Franz, F.X. McCormack, G. Chuck, J. Leonard, L. Young, and J. Elwing. Rapamycin therapy of angiomyolipomas s in patients with TSC and sporadic LAM*.* p. Unpublished data presented at TSC Conference, Cambridge, UK, Sept. 2004.

40. Wienecke, R., I. Fackler, N. Akdeli, U. Linsenmaier, M. Vogeser, K. Mayer, T. Licht, and M. Kretzler. Effective treatment of TSC with rapamycin*.* p. Unpublished data presented at TSC Conference, Cambridge, UK, Sept. 2004.

41. Brattstrom, C., J. Sawe, B. Jansson, A. Lonnebo, J. Nordin, J.J. Zimmerman, J.T. Burke, and C.G. Groth. Pharmacokinetics and safety of single oral doses of sirolimus (rapamycin) in healthy male volunteers*.* Therapeutic Drug Monitoring, 2000. 22(5): p. 537-44.

42. MacDonald, A.S. and e. al. A worldwide, phase III, randomized, controlled, safety and efficacy study of a sirolimus/cyclosporine regimen for prevention of acute rejection in recipients of primary mismatched renal allografts*.* Transplantation, 2001. 71(2): p. 271-80.

43. Mahalati, K. and B.D. Kahan. Clinical pharmacokinetics of sirolimus*.* Clinical Pharmacokinetics, 2001. 40(8): p. 573-85.

44. Yatscoff, R.W. Pharmacokinetics of rapamycin*.* Transplantation Proceedings, 1996. 28(2): p. 970-3.

45. Roach, E.S., M.R. Gomez, and H. Northrup. Tuberous sclerosis complex consensus conference: revised clinical diagnostic criteria*.* J Child Neurol, 1998. 13(12): p. 624-8.

46. Dabora, S.L., S. Jozwiak, D.N. Franz, P.S. Roberts, A. Nieto, J. Chung, Y.S. Choy, M.P. Reeve, E. Thiele, J.C. Egelhoff, J. Kasprzyk-Obara, D. Domanska-Pakiela, and D.J. Kwiatkowski. Mutational Analysis in a Cohort of 224 Tuberous Sclerosis Patients Indicates Increased Severity of TSC2, Compared with TSC1, Disease in Multiple Organs*.* Am J Hum Genet, 2001. 68(1): p. 64-80.

47. Heymach, J.V., J. Desai, J. Manola, D.W. Davis, D.J. McConkey, D. Harmon, D.P. Ryan, G. Goss, T. Quigley, A.D. Van den Abbeele, S.G. Silverman, S. Connors, J. Folkman, C.D. Fletcher, and G.D. Demetri. Phase II study of the antiangiogenic agent SU5416 in patients with advanced soft tissue sarcomas*.* Clin Cancer Res, 2004. 10(17): p. 5732-40.

**10.1 INTERNET RESOURCES**

RECIST Criteria, <http://cancer.gov/bip/RECIST.htm>

NCI Common Terminology Criteria for Adverse Events v3.0 (CTCAE), <http://ctep.cancer.gov/reporting/ctc.html>

Statistical power calculation program, <http://hedwig.mgh.harvard.edu/sample_size/size.html>

**11.0 REGULATORY REQUIREMENTS**

11.1 Declaration of Helsinki – The overall principal investigator along with co-investigators and clinical site principal investigators will ensure that this study is conducted in full conformity with the current revision of the Declaration of Helsinki and with the US FDA Regulations, whichever affords the greater protection to the patient.

11.2 Informed Consent – In accordance with US FDA Regulations all participating clinical site principal investigators will be responsible for obtaining written informed consent from the patient or patient’s legally authorized guardian/representative after adequate explanation of the aims, methods, anticipated benefits, and potential hazards of the study and before any study medications are administered. The patient (or guardian) will be given a copy of the informed consent documentation that is labeled “PATIENT’S REFERENCE COPY”. The original signed copy of the informed consent will be retained in the Institution’s medical records.

11.3 Patient Confidentiality – All participating investigators will ensure that the patient’s anonymity is maintained.

In compliance with US FDA Regulations regarding the monitoring of clinical trials, the institute shall permit US FDA representatives to review the portion of the patient’s medical record that is directly related to the study. As part of the required content of informed consent, the patient must be informed that his/her records may be reviewed by a representative of the US FDA.

**12.0 LIST OF APPENDIX DOCUMENTS**

TSC clinical features grading form

(Appendix 1)

Cytochrome P450 3A4 substrates, inhibitors and inducers

(Appendix 2)

Response Evaluation Criteria in Solid Tumors (RECIST) Quick Reference

(Appendix 3)

NCI Common Terminology Criteria for Adverse Events v3.0 (CTCAE) Quick Reference

(Appendix 4)

Rapamune Product Information (annotated version, updated on 1/21/04)

(Appendix 5)

Collection of blood, urine and tissue specimens from patients with TSC or LAM

(Appendix 6)
