# Supplementary material for: Multicenter Phase 2 Trial of Sirolimus for Tuberous Sclerosis: Kidney Angiomyolipomas and Other Tumors Regress and VEGF- D Levels Decrease
Source: PLoS One. 2011 Sep 6;6(9):e23379. doi: 10.1371/journal.pone.0023379 (PMC3167813; doi:10.1371/journal.pone.0023379)
Supplement: Supplementary file 20 [file pone.0023379.s005.doc]

Graphs of FVC at baseline and week 52 for indicated LAM subgroups

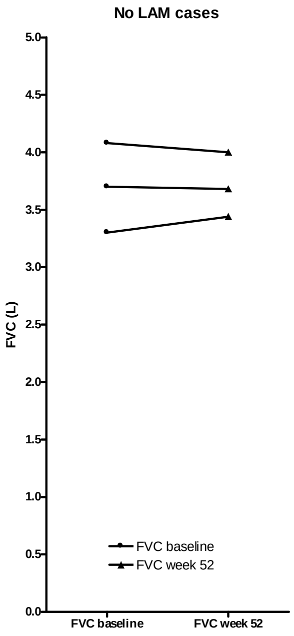

FVC (no LAM)

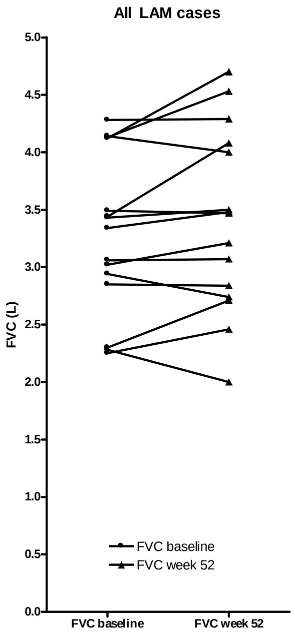

FVC (all LAM cases)

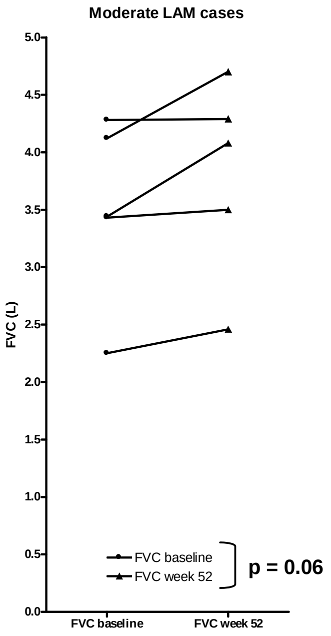

FVC (Moderate LAM)

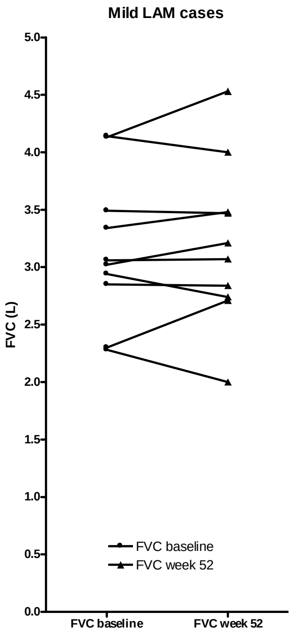

FVC (Mild LAM)
